# Diagnostic kit for rice blight resistance

**DOI:** 10.1038/s41587-019-0268-y

**Published:** 2019-10-28

**Authors:** Joon-Seob Eom, Dangping Luo, Genelou Atienza-Grande, Jungil Yang, Chonghui Ji, Van Thi Luu, José C. Huguet-Tapia, Si Nian Char, Bo Liu, Hanna Nguyen, Sarah Maria Schmidt, Boris Szurek, Casiana Vera Cruz, Frank F. White, Ricardo Oliva, Bing Yang, Wolf B. Frommer

**Affiliations:** 1 Institute for Molecular Physiology and Cluster of Excellence on Plant Sciences (CEPLAS), Heinrich Heine University of Düsseldorf, Düsseldorf, Germany; 2 Max Planck Institute for Plant Breeding Research, Cologne, Germany; 3 Division of Plant Sciences, Bond Life Sciences Center, University of Missouri, Columbia, MO USA; 4 International Rice Research Institute, Metro Manila, Philippines; 5 Department of Plant Pathology, University of Florida, Gainesville, FL USA; 6 Department of Genetics, Development and Cell Biology, Iowa State University, Ames, IA USA; 7 IRD, CIRAD, Université Montpellier, IPME, Montpellier, France; 8 Donald Danforth Plant Science Center, St. Louis, MO USA; 9 Institute of Transformative Bio-Molecules (WPI-ITbM), Nagoya University, Aichi, Japan; 10 Present Address: College of Agriculture and Food Science, University of the Philippines, Los Baños, Philippines

**Keywords:** Pathogenesis, Plant biotechnology, Plant breeding, Infection, Biotechnology

## Abstract

Blight-resistant rice lines are the most effective solution for bacterial blight, caused by *Xanthomonas oryzae* pv. *oryzae* (*Xoo*). Key resistance mechanisms involve *SWEET* genes as susceptibility factors. Bacterial transcription activator-like (TAL) effectors bind to effector-binding elements (EBEs) in *SWEET* gene promoters and induce *SWEET* genes. EBE variants that cannot be recognized by TAL effectors abrogate induction, causing resistance. Here we describe a diagnostic kit to enable analysis of bacterial blight in the field and identification of suitable resistant lines. Specifically, we include a *SWEET* promoter database, RT–PCR primers for detecting *SWEET* induction, engineered reporter rice lines to visualize SWEET protein accumulation and knock-out rice lines to identify virulence mechanisms in bacterial isolates. We also developed CRISPR–Cas9 genome-edited Kitaake rice to evaluate the efficacy of EBE mutations in resistance, software to predict the optimal resistance gene set for a specific geographic region, and two resistant ‘mega’ rice lines that will empower farmers to plant lines that are most likely to resist rice blight.

## Abstract (web summary)

Strategic deployment of blight-resistant rice lines is enabled by a molecular diagnostic kit.

## Main

Rice is the most important food crop in the world, but biotic and abiotic stresses can reduce yields. *Xoo* causes the rice disease bacterial blight, which results in substantial yield losses across Asia^1^. Bacterial blight has been classed as the most serious bacterial disease of rice^2^, with the highest social impact. Epidemics severely affect smallholders: about 70% of farms in India are ~ 0.39 ha (ref. ^3^), roughly similar to 80% of farms in sub-Saharan Africa ( < 2 ha). Bacterial blight is a major problem in India that has increased in severity year on year since 2000 (ref. ^4^). Increased severity has been attributed in part to climate change (increased rainfall and higher cyclone frequency)^4^. Modeling indicates that climate change might result in an increased effect of *Xoo*-mediated disease in Africa, where rice production is rising, and researchers have suggested that losses due to rice blight would eventually exceed those caused by rice blast^5^. Moreover, the United States is concerned about the potential for introduction of Asian or African bacterial blight strains; currently, only low-virulence strains that lack TAL effector genes are present in the United States^6^. *Xoo* is on the US Select Agent list as a potential bioterrorism agent^7^.

Genetic resistance to disease reduces the need for pesticides^8^. The best known plant resistance (*R*) genes encode proteins that interact with specific pathogen effectors and endow plants with dominant resistance to pathogens. More than 40 *R* genes for bacterial blight have been identified; a few of these have been cloned, and several have been associated with modular TAL effectors^8^. Complete genome sequences of *Xoo* enable identification of TAL effectors, information that can then be used together with the TAL effector recognition code^9,10^ to identify the respective TAL EBEs throughout the rice genome. In some cases, however, resistance is recessive and caused by effectors that target susceptibility factors, as in the case of host sucrose transporter (*SWEET*) genes. *Xoo* produces TAL effectors that ectopically induce the *SWEET* genes that make rice susceptible to infection and disease^1,12^.

**Fig. 1:**
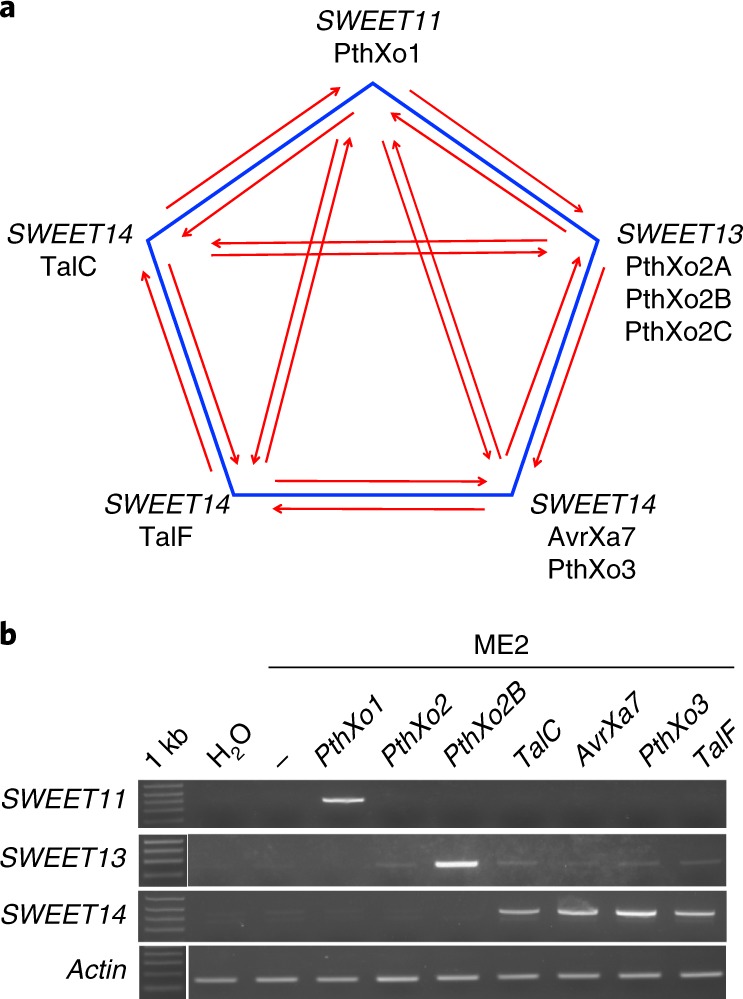
*SWEET11*–*SWEET13*–*SWEET14* EBE pentagon and PCR detection of *SWEET* induction. **a**, Arrows indicate which TAL effectors can overcome a particular resistance mechanism by activating any of the other *SWEET* genes or by activating the same *SWEET* gene via targeting another EBE in the same promoter. For example, *xa13*-based resistance (resulting from a variant in the *SWEET11* promoter that is not recognized by PthXo1) can be overcome by other TAL effectors (e.g., PthXo2 and PthXo3) that target the EBEs in another *SWEET* gene promoter or, in the case of *SWEET14*, target different EBEs in the same promoter. **b**, Example of *SWEET* gene induction as detected by RT–PCR using the SWEET^up^ primer set. RT–PCR products are shown for the *SWEET11*, *SWEET13* and *SWEET14* genes in Kitaake infected by *Xoo* strain ME2 lacking a *SWEET*-targeting TAL effector and ME2 transformed with plasmids encoding PthXo1 (targeting *SWEET11*), PthXo2 or PthXo2B (both targeting *SWEET13*) and PthXo3, TalC, TalF or AvrXa7 (all targeting *SWEET14*). *Actin* served as a control. Leaves were infected using leaf-clipping assays; scissors dipped in water served as an additional negative control. The experiment was repeated twice independently with similar results.

DNA polymorphisms in EBEs can prevent TAL effectors from binding to target promoters, and the respective rice lines with altered EBEs in a *SWEET* promoter are resistant to bacterial blight^11,12^. The first identified *SWEET* resistance variant was *xa13*, a naturally occurring promoter variant in *SWEET11* (refs. ^13,14^). *xa13* resistance is recessive and is used in rice breeding programs, as the *xa13* promoter variants do not negatively affect yield^4,15^. The TAL effector PthXo1, which is present in several *Xoo* strains (PXO99 and PXO71), binds an EBE in the *SWEET11* promoter^11^. The resistant rice line IRBB13 (*xa13*) carries a 38-bp deletion and a 252-bp insertion in the *SWEET11* promoter, which abrogate binding of the EBE by PthXo1. Other resistant varieties carrying *xa13* include Chinsurah Boro2, Tepa1, Aus274, AC-19-1-1, Long Grain (35023), Kalimekri77-5, Long Grain (64950) and BJ1 (ref. ^13^). *xa13* resistance can be overcome by *Xoo* strains that produce alternative TAL effectors that bind to the promoter of a different *SWEET* paralog. For example, *SWEET14* promoter elements can be bound by PthXo3 or AvrXa7 (ref. ^16^; Fig. 1a). Naturally occurring recessive resistance has also been identified for *SWEET13* (*xa25*)^17,18^ and *SWEET14* (*xa41*)^19^. Altogether, six EBEs in three *SWEET* genes that are targeted by naturally occurring TAL effectors (PthXo1 targeting *SWEET11*; PthXo2 and variants targeting *SWEET13*; PthXo3, AvrXa7, TalC and TalF targeting *SWEET14*) have been characterized. The TAL effectors target three of the five clade III *SWEET* genes; the other two *SWEET* genes can function as susceptibility (*S*) genes when artificially induced, but no *Xoo* strains targeting these genes have been identified^20^. *SWEET* genes in other clades do not function as *S* genes^20^.

The pool of naturally occurring EBE variants is small, meaning that extensive breeding efforts are needed to identify and introduce such resistance variants into elite rice varieties. TALEN- or CRISPR-based genome editing has been used to engineer EBE variants of *SWEET11* and *SWEET14* in the *japonica* variety Kitaake^18,19,21^. Additionally, five EBEs have been targeted, alone or in combination, in Kitaake, IR64 and Ciherang-Sub1 (refs. ^18,21–24^), generating resistance to a collection of 94 *Xoo* strains of Asian origin^24^. These *R*-gene-containing lines can now be deployed, individually or in combination, for broad resistance. However, *Xoo* will likely evolve TAL effector variants that can bind to other promoter sequences to enable plant infection.

To enable the strategic deployment of *R* genes to block novel virulent strains of *Xoo* when they emerge, we developed the SWEET^R^ kit 1.0, a multicomponent tool set that integrates pathogen diagnostics, genetic resistance and customizable resistance line deployment to overcome bacterial blight.

## Results

### Content of the SWEET^R^ kit 1.0

The SWEET^R^ kit 1.0 (Supplementary Table 1) contains a *SWEET* promoter database (SWEETpDB), SWEET^up^ primers for detecting mRNA accumulation for each *SWEET* gene, three SWEET^acc^-rice tester lines that report spatial SWEET protein accumulation during infection using translational reporter fusions^26^, single- and combined-knockout SWEET^ko^ mutants to identify which *SWEET* genes are required for susceptibility to individual *Xoo* strains, SWEETp^R^ tester lines in the *R*-gene-free Kitaake background to evaluate whether particular EBE variants (or combinations thereof) are sufficient for resistance against an *Xoo* isolate, SWEET PathoTracer, a decision tool^26^ based on disease diagnostic surveys and population information to develop the most effective and most durable resistance for a specific region, and a total of 32 transgene-free EBE-edited lines of two mega varieties: IR64 and Ciherang-Sub1 (ref. ^24^).

### *SWEET* promoter database

To predict resistance against, or susceptibility to, any *Xoo* strain with TAL effector(s) that target *SWEET* promoters, the EBE sequences in *SWEET* promoters must be characterized. We analyzed EBE variations for *SWEET11*, *SWEET13* and *SWEET14* in 4,726 accessions (in the first 400 bp of the *SWEET* promoters)^27,28^ as well as 5 rice lines grown in India, Southeast Asia and Africa, and identified 15 sequence variants. One A/G variant in the EBE for PthXo1 occurred at a frequency of 0.2% (Supplementary Fig. 1 and Supplementary Table 2)^13,29^. Seven variations were found in the PthXo2 EBE at frequencies between 1.3% and 20.8% (Supplementary Fig. 2 and Supplementary Table 2)^29^. Notably, five variations were found at the TATA box of *SWEET13* (TATATAAA, TATTTAAA, TATATATA, TATATAA and TATATAAAA), which overlap with the EBE. The TATATAAA variant is known to occur in *japonica* varieties resistant to *Xoo* strains that harbor the TAL effector PthXo2 (ref. ^18^). In the PthXo3/AvrXa7 EBE, an insertion of one adenosine was found at a frequency of 7.7% (Supplementary Fig. 3 and Supplementary Table 2)^29^. Lines CX371 and CX372 (or NERICA1 and NERICA2) carried a G/T variation in the TalC EBE and an 18-bp deletion spanning the PthXo3/AvrXa7 and TalF EBEs. These variations could lead to broad-spectrum resistance against *Xoo* strains harboring TalC, PthXo3/AvrXa7 and TalF. To confirm the information mined from genomic sequences, 2 of the 4,726 accessions for each variation type and 5 lines grown in India, Southeast Asia and Africa (MTU1010, Samba Mahsuri, Komboka, BRRI Dhan28 and BRRI Dhan 29) (Supplementary Table 3) were chosen for validation. The first 400 bp of the three *SWEET* promoters were sequenced and included in a representative promoter sequence database (sequences in Supplementary Table 4).

### SWEET^up^ primers to detect *SWEET* mRNA levels

To enable rapid assessment of *SWEET* mRNA accumulation upon *Xoo* infection, we synthesized specific primer pairs for *SWEET11*, *SWEET13* and *SWEET14* (named SWEET^up^) and established robust RT–PCR protocols (Supplementary Table 5 and Supplementary Note 1). *SWEET* mRNA levels were detected in uninfected leaves (Supplementary Fig. 4) and in infected leaves in a strain-specific manner (Fig. 1b). Quantitative RT–PCR (qRT–PCR) showed that *SWEET13* mRNA levels were highest among the five clade III *SWEET* genes in uninfected leaves, with lower levels of *SWEET14*. *SWEET13* may have a role in phloem loading, as shown for its maize homologs^30^. *SWEET11* mRNA levels were very low in leaves, consistent with its roles in seed filling^26^. Validated primer pairs are available in the kit for testing new *Xoo* isolates.

### SWEET^acc^-rice tester lines for SWEET protein accumulation

**Fig. 2:**
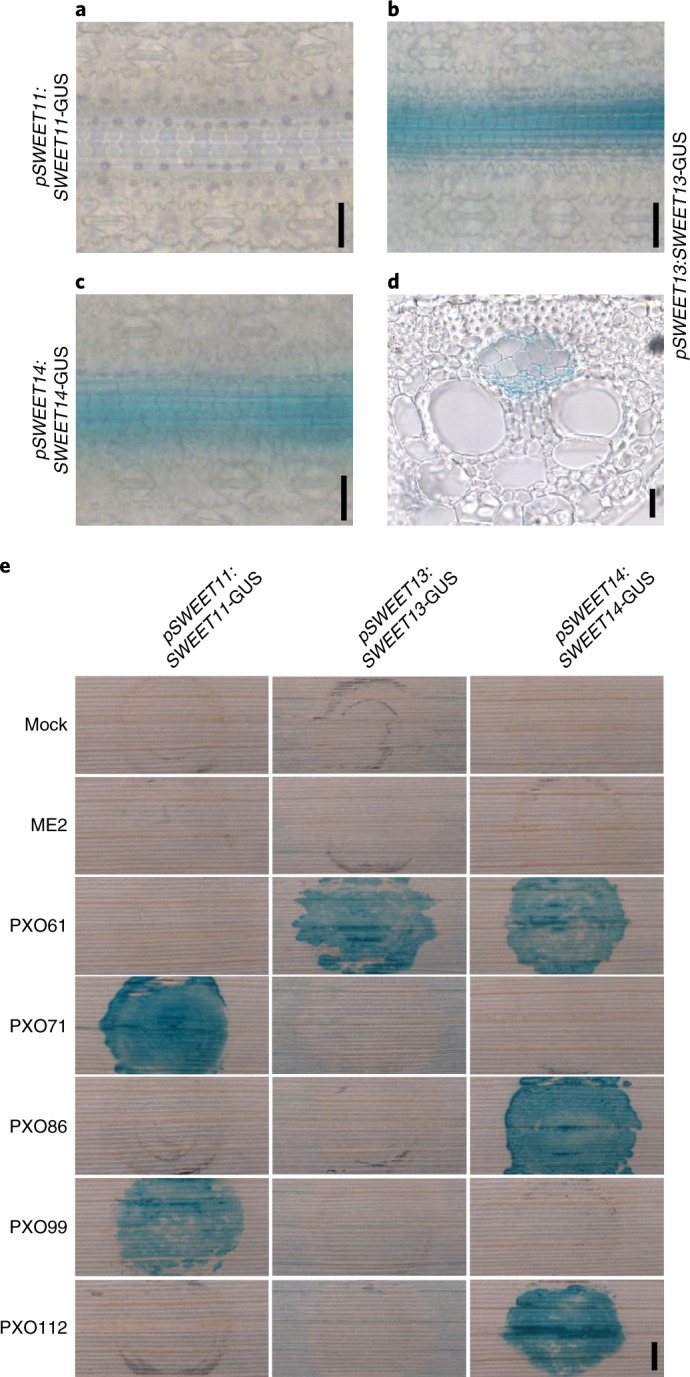
SWEET protein accumulation in uninfected and infected transgenic rice leaves. **a–c**, GUS activity in flag leaf blades of rice for *pSWEET11*:*SWEET11-GUS* (event 10) (**a**), *pSWEET13*:*SWEET13-GUS* (event 22) (**b**) and *pSWEET14*:*SWEET14-GUS* (event 3) (**c**). **d**, A cross-section of the leaf blade from *pSWEET13*:*SWEET13-GUS* in **b**. **e**, SWEET protein accumulation upon infection with *Xoo* strains (*pSWEET11*:*SWEET11-GUS* event 10, *pSWEET13*:*SWEET13-GUS* event 15 and *pSWEET14*:*SWEET14-GUS* event 3). Scale bars: 20 µm (**a–d**); 1 mm (**e**). The experiment was repeated at least three times independently with similar results.

First, we engineered transcriptional reporter lines to visualize *SWEET* RNA accumulation by histochemical β-glucuronidase (GUS) staining. However, reporter lines for all three promoters had nonspecific reporter activity (Supplementary Fig. 5). Previously, similar observations were reported for *AtSWEET11* and *AtSWEET12* in *Arabidopsis*; only translational SWEET reporters had cell-specific reporter activity^12^. To monitor SWEET protein accumulation, translational promoter reporter lines were engineered. The constructs included a 2-kb fragment from the *SWEET* promoter, the coding region of the *SWEET* gene including introns and a *GUSPlus* reporter (Supplementary Figs. 6–8). Consistent with the absolute mRNA levels measured by qRT–PCR, *SWEET13* and *SWEET14* translational promoter reporter activities were detected in uninfected leaves, whereas *SWEET11* translational promoter fusion lines showed no detectable GUS activity (Fig. 2a–d). *SWEET13* and *SWEET14* translational reporter lines showed vein-specific protein accumulation, consistent with the roles of *SWEET13* and *SWEET14* in phloem loading (Fig. 2b–d). Three reporter lines for *SWEET11*, *SWEET13* and *SWEET14*, named SWEET^acc^-rice tester lines, were each infected with five *Xoo* strains (PXO61, PXO71, PXO86, PXO99 and PXO112), which are known to induce specific *SWEET* genes. The *Xoo* strain ME2, lacking TAL effectors for *SWEET* induction^14^, was used as a control and did not trigger SWEET^acc^ reporter activity. Induction of *SWEET11* was detected upon infection with PXO71 and PXO99 (both of which harbor PthXo1) but not with the other strains. *SWEET13* was induced only upon infection with PXO61 (harboring PthXo2B), whereas *SWEET14* was induced upon infection with PXO61 (PthXo3), PXO86 (AvrXa7) and PXO112 (PthXo3; Fig. 2e). Infection with ME2 strains harboring PthXo1 (targeting *SWEET11*), PthXo2B (targeting *SWEET13*) or PthXo3, TalC, AvrXa7 and TalF (targeting *SWEET14*) further confirmed specific *SWEET* isoform induction by this set of reporter lines (Supplementary Fig. 9).

### SWEET^ko^ knock-out lines to detect *Xoo SWEET* targets

**Fig. 3:**
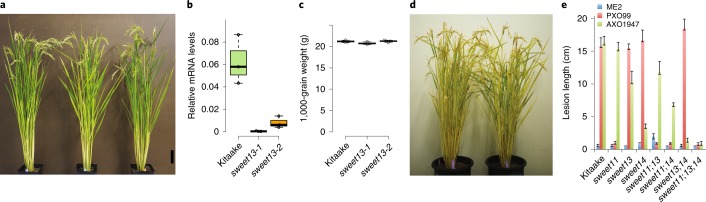
SWEET^ko^ knockout mutants as diagnostic tools. **a**, Phenotypes of *sweet13-1* and *sweet13-2* knockout mutants relative to Kitaake controls at the mature stage. Scale bar, 10 cm. **b**, Relative mRNA levels of *SWEET13* in flag leaf blades. Samples were harvested at 12:00 (mean ± s.e.m., *n* = 3 biological replicates with mRNA levels normalized to rice *Ubiquitin1* levels; repeated independently three times with similar results). Center lines show medians; box limits indicate the 25th and 75th percentiles as determined by R software; and whiskers extend 1.5 times the interquartile range from the 25th and 75th percentiles. **c**, One-thousand-grain weight of greenhouse-grown Kitaake, *sweet13-1* and *sweet13-2* (mean ± s.e.m.; *n* = 4 biological replicates). The experiment was repeated at least three independent times with similar results. Center lines show medians; box limits indicate the 25th and 75th percentiles as determined by R software; and whiskers extend 1.5 times the interquartile range from the 25th and 75th percentiles. No significant differences (*P* = 0.051 for *sweet13-1* and *P* = 0.758 for *sweet13-2*) were identified by Student’s *t*-test. **d**, Phenotypes of wild type and the *sweet13*;*sweet14* double knockout grown in the greenhouse. No significant differences were identified. **e**, Length of lesions at 14 days after inoculation (DAI) caused by ME2 (negative control), PXO99 (positive control) and AXO1947 on single-, double- and triple-knockout (*sweet11*, *sweet13* and *sweet14*) mutants relative to Kitaake wild type (mean ± s.e.m.; *n* = 10 inoculated leaves). The experiment was independently repeated twice with similar results. The difference observed for AXO1947 virulence between *sweet14* and *sweet11;14* in a single experiment was not significant when compared over a larger number of experiments (Supplementary Fig. 14).

Tools that could identify the *SWEET* genes targeted by a specific *Xoo* strain, without previous knowledge of the targeted EBE, would inform efforts to engineer resistance. This is especially important because variant *Xoo* strains can target either different promoter elements or other clade III *SWEET* paralogs. Moreover, it is conceivable that promoter-edited lines or variants could impair yield, if the promoter variations affect normal gene function in uninfected plants. Knowledge of the specific defects is thus important to judge possible negative effects of such mutations on plant performance. *SWEET*-mutant lines could also provide insight into the role(s) of *SWEET* genes in resistance and yield. To diagnose which *SWEET* was targeted by any *Xoo* strain and to predict the yield effects of alterations in *SWEET* genes, knockout mutants were created for four of the five described clade III *SWEET* genes (*SWEET11*, *SWEET13*, *SWEET14* and *SWEET15*) using CRISPR–Cas9 (Supplementary Fig. 10 and ref. ^26^). In all cases, lines containing frameshift mutations in the sequence corresponding to transmembrane domain I (TM I) were identified (Supplementary Table 6). Frameshifts that result in early termination should create nonfunctional transporters. For example, premature termination in the last transmembrane domain, TM VII, of OsSWEET11 results in defective transporters^12^. *sweet13* mutant lines had reduced *SWEET13* mRNA levels, which often occur concurrently with early termination (Fig. 3b). Knockout mutants for *SWEET11* and *SWEET15* were reported to have defects in seed filling^26^. Although *SWEET11* and *SWEET15* have important roles in seed filling in rice, widely used promoter variants, such as the resistance-conferring *xa13* variant (*SWEET11*), do not seem to affect yield^4,15^.

Because maize *ZmSWEET13* paralogs have roles in phloem loading^30^, the role of rice *SWEET13* was investigated. *SWEET13* is the most highly expressed *SWEET* gene in rice leaves, and the encoded protein localizes to the plasma membrane and, in common with SWEET14, transports sucrose (Supplementary Figs. 4 and 11)^12,18^. Analysis of GUS reporter fusions showed that SWEET13 accumulates in the phloem (Fig. 2b,d). SWEET14 also accumulated in the phloem but had substantially lower mRNA levels in leaves than SWEET13 (Fig. 2c; transcript levels not shown). Nevertheless, CRISPR–Cas9 *sweet13* and *sweet14* knockout mutant lines did not show detectable growth or yield defects under greenhouse conditions (Fig. 3a,c and Supplementary Fig. 12), nor were obvious differences observed in a single-season field experiment (based on visual inspection during the growth period and after harvest). To identify potential compensatory activity from other *SWEET* genes in the knockout mutants, the expression levels of other sucrose-transporting *SWEET* genes were analyzed. Only the weakly expressed *SWEET14* and *SWEET15*, and none of the other clade III *SWEET* genes, showed substantial increases in mRNA accumulation in the leaf blade of *sweet13* knockout lines (Supplementary Fig. 13). To test whether upregulation of *SWEET14* could compensate for the loss of *SWEET13* and thereby restore apoplasmic phloem loading, *sweet14* single-knockout and *sweet13*;*sweet14* double-knockout lines were generated. Double mutants did not show obvious growth differences relative to controls in the greenhouse (Fig. 3d). Because mutant lines had no clear defects in plant growth or yield, EBE-edited lines in which the normal promoter function of *SWEET13* and *SWEET14* is affected are not hypothesized to have yield penalties. Further, our data indicate that apoplasmic phloem loading in rice, in contrast to maize and *Arabidopsis*, either is not crucial to plant performance or does not entirely depend on *SWEET* function^12,30^.

Knockout lines can serve as diagnostic tools for testing whether *Xoo* strains require specific *SWEET* genes. The knockout mutants proved to be valuable tools for characterizing the virulence of a collection of 95 different *Xoo* strains with diverse geographic origins^24^. In this analysis, we observed that an African strain, AXO1947, which contains the effector TalC and can induce *SWEET14*, but does not induce *SWEET13*, was still able to infect a Kitaake mutant line edited in the TalC EBE present in the *SWEET14* promoter^24^. Although technically weakly virulent on lines carrying TalC EBE variants, AXO1947 showed substantially reduced virulence in the quintuple-mutant promoter lines, which are likely sufficiently resistant in field conditions^24^. The quintuple-mutant line was moderately resistant, with lesion lengths of 5–7 cm upon infection by AXO1947, as compared to 18–25 cm in controls. A systematic screen for resistance using *sweet13* and *sweet14* single-knockout and *sweet13*;*sweet14* double-knockout mutants showed that AXO1947 lost some virulence in *sweet14*-knockout lines but was unable to infect *sweet13*;*sweet14* double mutants (Fig. 3e and Supplementary Fig. 14). These data demonstrate the utility of knockout lines for testing resistance (Fig. 3e, Table 1 and Supplementary Table 7). Co-dependence of strain AXO1947 on both *SWEET13* and *SWEET14* function is under investigation. Further characterization is needed, as dependence on *SWEET13* is not understood, given that *SWEET13* induction by AXO1947 was not detected. Notably, whereas in leaves basal *SWEET11* mRNA levels are low and induction is easily detectable, *SWEET13* and *SWEET14* are expressed in leaves; thus, a further increase in expression in a few cells in the xylem is difficult to detect against this background. SWEET^acc^ and SWEET^ko^ rice tester lines are thus better suited to detect *SWEET* dependence.

**Table 1:** Resistance of *sweet13;sweet14* double–knock-out mutants to Asian and African *Xoo* strains as determined by lesion length (in centimeters) from clipping assays

Strain	Origin	Kitaake	*sweet13;sweet14*
ME2	Lab	**0.5** **±** **0**	**0.6** **±** **0.1**
ME2:*PthXo2B*	Lab	19.2 ± 0.4	**0.7** **±** **0.2**
ME2:*TalC*	Lab	8.2 ± 0.7	**0.6** **±** **0.1**
PXO86	PHL	12.4 ± 0.8	**1.3** **±** **0.2**
PXO61	PHL	12.3 ± 0.9	**1.3** **±** **0.4**
PXO404	PHL	13.6 ± 1.1	**1.2** **±** **0.2**
PXO364	PHL	16.3 ± 0.7	**0.9** **±** **0.3**
PXO421	PHL	16.9 ± 0.2	**0.9** **±** **0.2**
PXO513	PHL	12.5 ± 1.5	**0.8** **±** **0.1**
KXO85	Korea	13.7 ± 1.7	**1.7** **±** **0.2**
JW89011	Korea	16.2 ± 1.4	**2.1** **±** **0.3**
AXO1947	Africa	13.1 ± 0.1	**1.6** **±** **0.3**
CFBP1948	Africa	12.5 ± 2.3	**1.8** **±** **0.6**
CFBP1949	Africa	15.2 ± 2.1	**2.3** **±** **0.8**
CFBP1951	Africa	11.7 ± 0.9	**2.1** **±** **0.4**
CFBP1952	Africa	12.0 ± 1.5	**1.2** **±** **0.3**
CFBP7319	Africa	14.7 ± 2.6	**2.0** **±** **0.2**
CFBP7320	Africa	14.3 ± 1.6	**2.1** **±** **0.2**
BAI3	Africa	15.5 ± 2.8	**2.4** **±** **0.3**
CFBP7322	Africa	14.8 ± 1.7	**1.2** **±** **0.2**
CFBP7323	Africa	17.8 ± 1.8	**2.2** **±** **0.2**
CFBP7324	Africa	16.7 ± 0.9	**1.9** **±** **0.1**
MAI1	Africa	15.8 ± 1.1	**1.1** **±** **0.3**
CFBP7337	Africa	16.7 ± 1.3	**2.4** **±** **0.6**
CFBP7340	Africa	15.8 ± 2.7	**2.4** **±** **0.3**
CFBP8172	Africa	11.7 ± 0.6	**2.3** **±** **0.3**
Dar16	Africa	14.7 ± 1.8	**2.1** **±** **0.4**
T19	Africa	16.8 ± 2.4	**2.1** **±** **0.5**
Ug11	Africa	15.7 ± 1.3	**1.4** **±** **0.2**

Bold font indicates resistance. Lesion lengths (mean ± s.e.m.; *n* = 10) were derived from ten leaves at 14 DAI. The disease assay was repeated twice independently with similar results. PHL, the Philippines.

### SWEETp^R^, genome-edited EBE tester lines for *Xoo* genotyping

Rice varieties have different numbers and types of *R* genes. The only known *R* gene for bacterial blight in the *japonica* rice variety Kitaake is a cryptic resistance gene similar to the recessive *xa25*, which interacts with the major TAL effector PthXo2 (ref. ^18^). Thus, Kitaake is a useful reference line for testing *Xoo* compatibility. In a parallel study^24^, a series of EBE variants of *SWEET11*, *SWEET13* and *SWEET14* in Kitaake were engineered by genome editing, and resistance/susceptibility to *Xoo* strains was validated^24^. These 20 genome-edited Kitaake tester lines (named SWEETp^R^)^24^ are available for genotyping *Xoo* isolates and function similarly to *R*-gene line panels for race characterization^31^ (e.g., Kitaake line 11.1-45 was resistant to strains containing the TAL effectors PthXo1 and AvrXa7, and line 12.2-12 was resistant to strains containing PthXo2B, PthXo3 and AvrXa7 (ref. ^24^)) (Supplementary Tables 8 and 9).

### SWEET PathoTracer visualization

**Fig. 4:**
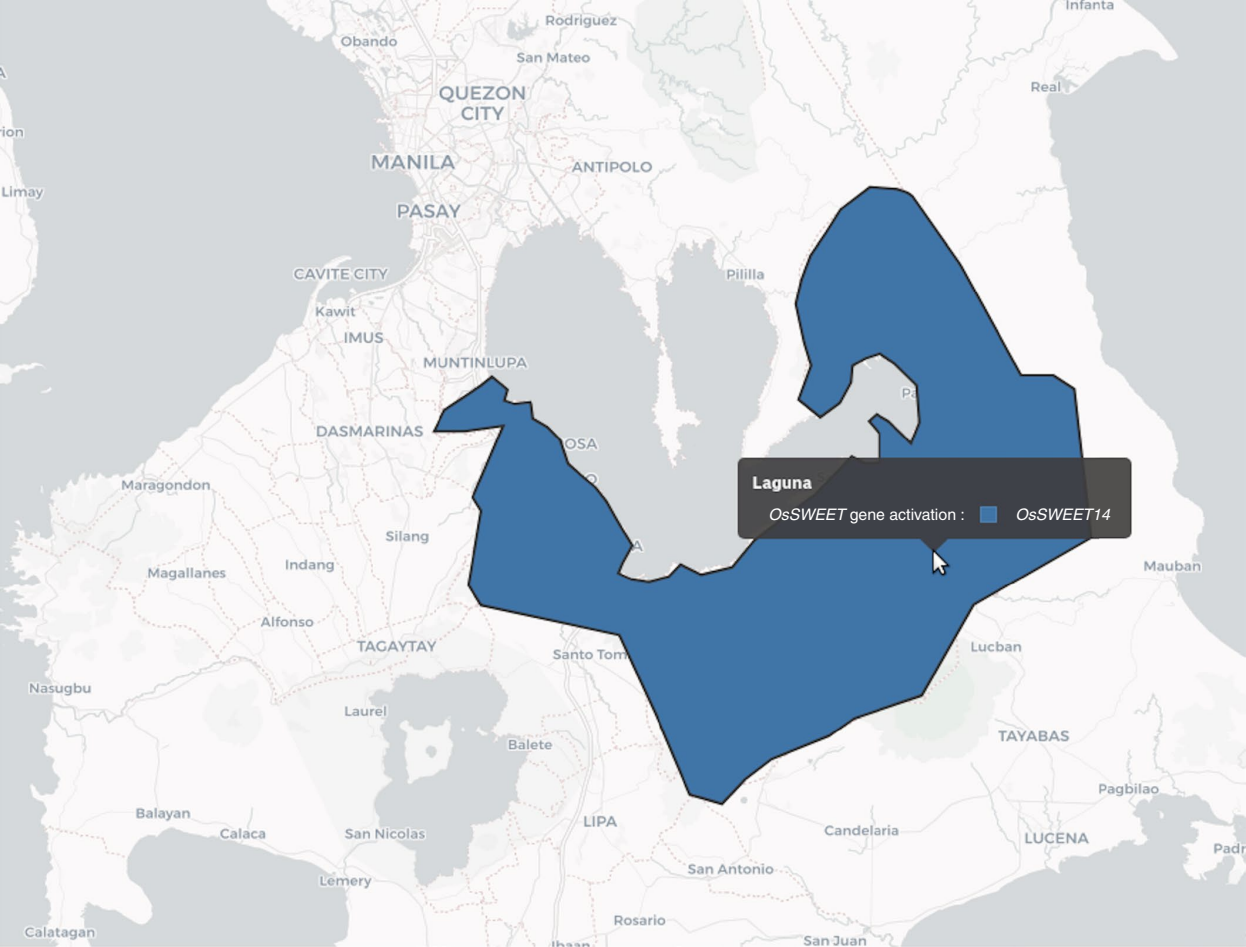
PathoTracer visualization showing prevalence of *Xoo* strains with putative *SWEET14* induction in the Philippines. PathoTracer (http://webapps.irri.org/pathotracer/index.html) is an online repository that integrates genotypic and phenotypic pathogen data with the resistance profiles of rice accessions to support strategic deployment of varieties in the region. Tester- strain-based prediction of SWEET targets is provided here for SWEET14, using *Xoo* populations collected from 1972 to 2012 in Laguna, a disease-endemic area in the Philippines (*n* = 1,294 isolates). A screenshot of the full interface with the same map is shown in Supplementary Fig. 15.

Geographic Information System (GIS)-based platforms that incorporate pathogen monitoring and resistance profiles of rice varieties are useful for management of local disease outbreaks^26^. We integrated the near-isogenic IR64 and Ciherang-Sub1 lines into the PathoTracer platform (http://webapps.irri.org/pathotracer/index.html). PathoTracer displays the predicted involvement of *SWEET11*, *SWEET13* and *SWEET14* on the basis of the *Xoo* population that is present in geographic regions and suggests effective edited variants for planting in the next season. For example, a dataset containing analyses about the ability of *Xoo* strains to infect the *R-*gene near-isogenic IRBB lines^32^ was compared to the proportion of endemic strains from an area of the Philippines that might activate *SWEET14* (Fig. 4 and Supplementary Fig. 15). On the basis of this information, 47% of the strains in the *Xoo* population are predicted to be controlled by one or more of the *SWEET14* EBE variants. By planting the recommended varieties, farmers can minimize the risk of infection and the resulting yield losses in the next season.

### Genome-edited *Xoo*-resistant mega variety lines

**Fig. 5:**
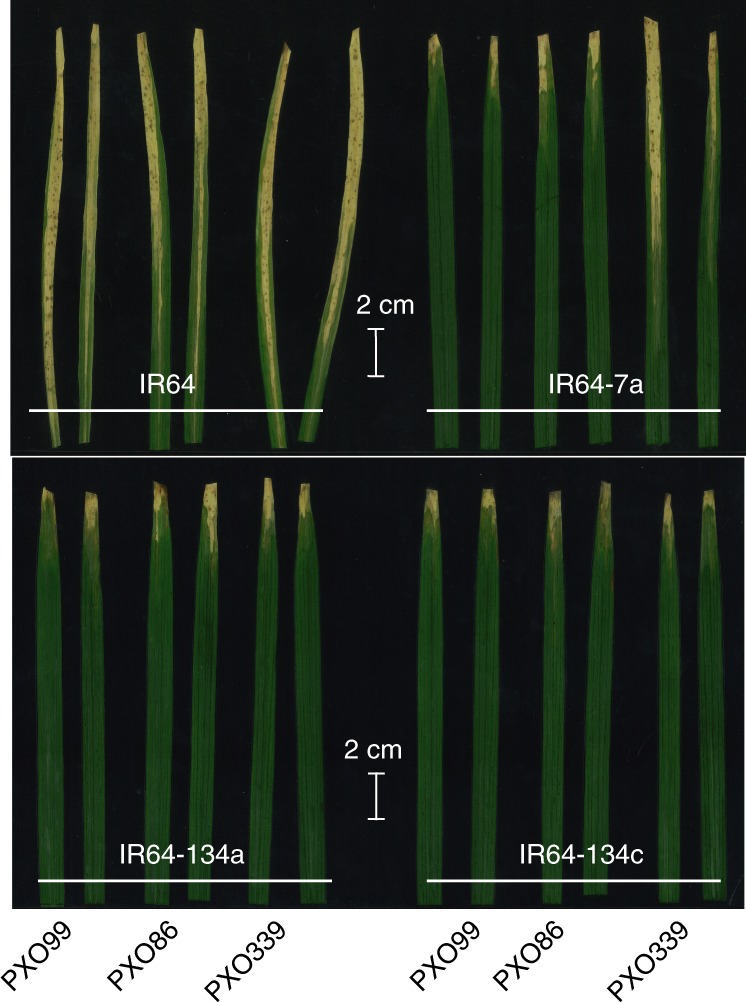
Resistance of genome-edited rice lines to different *Xoo* strains. Reactions of IR64 *SWEET*-promoter-edited lines to three representative *Xoo* strains (data from ref. ^24^). Lesion lengths were measured at 14 DAI with strains PXO99A, PXO339 and PXO86. Infections were carried out at the maximum tillering stage by inoculating 3–6 leaf samples using a leaf clipping. Four replicate experiments with two plants each were performed per strain (four replicates per strain, two plants per replicate (*n* = 8)) and scored for 3–6 inoculated leaf samples per plant. The experiment was repeated three times independently.

Mega rice varieties are defined as varieties planted on more than 1 million hectares. Although genome-edited bacterial-blight-resistant SWEETp^R^ Kitaake lines can be used by breeders, SWEET^R^ mega variety lines would reduce the need for further breeding efforts. This is of particular relevance because breeding efforts are more extensive in this context, owing to the recessive nature of *SWEET*-based resistance. CRISPR–Cas9 was used to edit five of the six EBE sites in the three *SWEET* promoters in widely used *indica* rice mega variety IR64 and in Ciherang-Sub1, a new flooding-tolerant elite line^24,33,34^. We generated edited lines with alterations in single or multiple EBEs. Together, 32 Cas9-free lines were produced, encompassing 35 single variations in the three *SWEET* promoters. Agronomic assessment and pathogenicity trials validated resistance against single or multiple *Xoo* strains (Fig. 5 and Supplementary Table 10)^24^.

## Discussion

Genetically narrow germplasm and extensive mono-cropping are two hallmarks of modern agriculture that favor disease and its spread^35^. Genome editing could provide efficient tools for rapid engineering of pathogen resistance in cropping lines. However, detection of pathogen strains, their virulence factors and cropping line susceptibilities will be crucial if we are to effectively reduce the impact of plant diseases. Here we present a diagnostic kit to enable breeders, crop management teams and farmers to reduce the effect of bacterial blight on rice yields worldwide.

Genome editing can create large pools of *R*-gene variants, providing new ways to implement and manage long-term genetic resistance. Diseases that use TAL effectors are excellent candidates for reducing yield loss by engineering resistant plant lines, because TAL effector binding depends on highly conserved and short cognate EBE sequences present in host *S*-gene promoters. One crucial finding in the fight against bacterial blight is that *Xoo* strains use different TAL effectors (eight effectors have been characterized to date) to directly target five EBEs in three *SWEET* gene promoters^24^. Pathogen-mediated induction of one of three *SWEET* genes is sufficient to cause disease. Extant *Xoo* strains harbor just eight *SWEET*-targeting TAL effectors, indicating that effectors with novel EBE recognition motifs may not evolve quickly. Therefore, genome editing could provide durable as well as broad bacterial blight resistance.

The success of genome-edited rice lines resistant to bacterial blight will be threatened by the emergence of pathogenic strains that have adapted to recessive resistance. The resistance endowed by *SWEET* promoter variants that prevent TAL effector binding can be overcome by the emergence of pathogenic *Xoo* strains. For example, resistance based on the natural promoter variant *xa13* can be overcome by *Xoo* strains that produce alternative TAL effectors, such as PthXo3 or AvrXa7, to induce either the same or different *SWEET* genes^16^. *SWEET* promoter variants will likely need to be deployed in combination with other *R* genes, and *Xoo* strain emergence may, therefore, depend on multiple genetic alterations.

The underlying key to durable resistance may be preventing bacteria from multiplying in monocultures^36^. Genome editing enabled us to establish a portfolio of recessive resistance variants with different promoter sequences that are available as *R-*gene variants to use when a novel pathogen emerges. The portfolio allows us to swap between *R-*gene variants. Tracking pathogens, using diagnostics and GIS, will likely also help. Because the edited lines are isogenic, they can be deployed as mixtures to reduce pathogen spread^37,38^, using mosaic crop plotting in which different *R* genes are deployed in adjacent fields or *R* genes are rotated over different seasons^38^. Here we provide a diagnostic tool set to accompany a suite of resistant rice lines^24^.

**Fig. 6:**
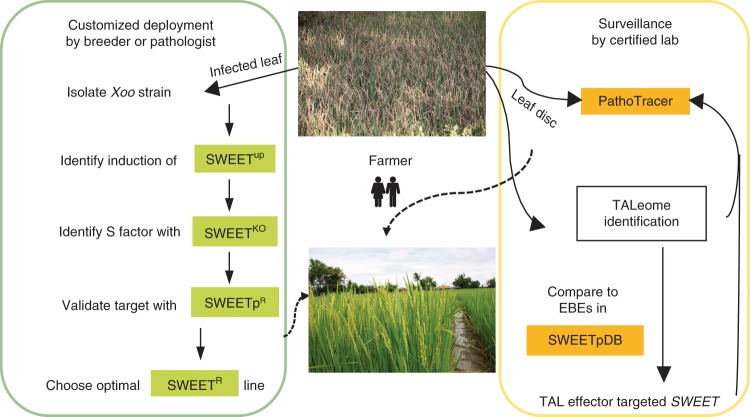
Customized deployment of SWEET^R^ lines with the help of the SWEET^R^ kit 1.0. Farmers with *Xoo*-infected rice fields will send samples to local breeders or pathologists, who will isolate respective *Xoo* strains. Pathologists will then identify induced and critically important *SWEET* genes using SWEET^up^ primers for mRNA accumulation and *SWEET* knock-out (SWEET^KO^) mutants. After validation with *SWEET* EBE-edited Kitaake lines (SWEETp^R^), pathologists will identify the optimal SWEET^R^ line, which is then provided to local breeders. In parallel, labs will isolate *Xoo* DNA from infected leaves, identify TAL effectors (the TALeome) and predict *SWEET* targets using SWEETpDB. PathoTracer provides additional region-specific recommendations for deployment of SWEET^R^ variants.

The SWEET^R^ kit comprises a SWEETpDB and SWEET^up^ primers to detect which *SWEET* is targeted by an *Xoo* isolate. If suitable expertise is not available locally, leaf samples can be preserved and analyzed in central test labs^39^. We also include four SWEET^acc^-rice tester lines containing full-gene translational reporters that detect a particular isolate that induces a *SWEET* gene^20,25^; this set includes a line for analysis of *SWEET15* induction, although so far no naturally occurring *Xoo* strains induce this *SWEET*. The kit further includes single- and combined-knockout SWEET^ko^ mutants to identify which *SWEET* genes are required for susceptibility and SWEETp^R^ tester lines in Kitaake. We added a component to a web-based decision tool^26^ named PathoTracer, which uses population information, to aid development of the most effective and most durable *R*-gene combination for a specific region through breeding. We also include 31 transgene-free EBE-edited lines in the two mega varieties IR64 and Ciherang-Sub1 (Supplementary Table 10)^24^. Figure 6 provides a roadmap for using the kit for customized deployment of the SWEET^R^ lines.

Recently, a new Gibson cloning technology for TAL effectors was developed that enables determination of the full TAL effector complement of a new isolate followed by bioinformatic target prediction, a tool that will be integrated into the kit^40^.

In the future, we will use our diagnostic kit to track newly emerging *Xoo* strains, use them to challenge *R*-gene variants and engineer promoter variants if TAL effectors evolve. Although only three of five clade III *SWEET* gene*s* have been targeted^24^, we have also developed *sweet15* knockout mutants that have no obvious growth defects in greenhouse and field conditions^24^. We plan to improve our kit by adding *sweet* knockout lines to cover all clade III *SWEET* genes. There is one report of an *Xoo* strain that does not induce *SWEET* genes^41^, but it is challenging to prove a lack of induction, as exemplified here for AXO1947.

*SWEET*-based resistance also occurs for bacterial blights of cotton and cassava, so our approach of providing a disease diagnosis and management kit together with a suite of genome-edited lines may prove useful for other pathogens that decimate crops such as cassava and cotton^42,43^.

## Methods

### Promoter variation analysis

Rice varieties having nucleotide variations in six EBEs (for the TAL effectors PthXo1, PthXo2, PthXo3, TalC, AvrXa7 and TalF) were found using the ‘Search for Variations in a Region’ and ‘Search for Genotype With Variation ID’ functions in RiceVarMap v.2 (http://ricevarmap.ncpgr.cn/v2/)^27^. Two varieties were selected for each variation type as representative. Sequences of the first 400 bp of *SWEET11*, *SWEET13* and *SWEET14* promoters of the selected varieties were subtracted from the 3K database (http://snp-seek.irri.org/). Alignment was performed using ClustalW2.1 in Geneious 11.1.5 (https://www.geneious.com).

#### Genotyping of rice plants

Rice genomic DNA was extracted using CTAB (http://gsl.irri.org/services/dna-extraction-king-fisher/met). PCR was performed using ExTaq DNA polymerase (Clontech) with a melting temperature of 56 °C for *SWEET11*, *SWEET13* and *SWEET14* (primers listed in Supplementary Table 5). The PCR amplicons from the mutant alleles were validated by Sanger sequencing. Chromatograms were analyzed and aligned using Sequencher (https://www.genecodes.com/).

#### RNA isolation and transcript analyses

Total RNA was isolated using Spectrum Plant Total RNA kits (Sigma) or TRIzol (Invitrogen), and first-strand cDNA was synthesized using a QuantiTect Reverse Transcription kit (Qiagen). qRT–PCR was performed using a LightCycler 480 (Roche), with the 2^−ΔCt^ method for relative quantification^44^. Primers for *SWEET11*, *SWEET13*, *SWEET14* and *UBI1* are listed in Supplementary Table 5.

### DNA constructs and plant transformation

#### Generation of GUS reporter constructs

The method for constructing *pSWEET11*:g*SWEET11*-GUSplus was described^26^. In short, the promoter and coding region were fused in frame to the GUSplus coding sequence with the NOS terminator, and the resulting constructs were PCR amplified and inserted into pC1300intC (GenBank AF294978.1)^45^. For tissue specificity analysis, a 4,354-bp genomic clone of *SWEET13* containing 1,919 bp of the 5′ region upstream of the translational start codon (ATG) and 2,435 bp of the entire coding region without a stop codon and a 4,365-bp genomic clone of *SWEET14* containing 2,176 bp of the 5′ upstream region and 2,189 bp of the entire coding region without a stop codon were amplified by PCR using Kitaake genomic DNA as a template (primers are listed in Supplementary Table 5). The PCR amplicons were subcloned into pJET2.1/blunt (Thermo Fisher), and resulting inserts were confirmed by DNA sequencing. Sequences are shown in Supplementary Table 11. The cloned fragments digested with XbaI and KpnI for *SWEET13* or AvrII and XmaI for *SWEET14* were subsequently inserted in front of the GUSplus coding sequence of a promoterless GUSplus coding vector^26^ restricted with XbaI and KpnI for *SWEET13* and XbaI and XmaI for *SWEET14*. The resulting *pSWEET13*:g*SWEET13*-GUSplus and *pSWEET14*:g*SWEET14*-GUSplus constructs were used to transform *Oryza sativa* L. ssp. *japonica* Kitaake. Nine independent events were obtained for *pSWEET13*:g*SWEET13*-GUSplus and *pSWEET14*:g*SWEET14*-GUSplus. Whereas GUS activity levels were different in the independent lines, the GUS patterns were similar.

Kitaake was also used for CRISPR–Cas9- and TALEN-mediated genome editing of *SWEET11*, *SWEET13* and *SWEET14*. The methods for the CRISPR–Cas9-induced mutant (*sweet11-1*) and the TALEN-induced mutant (*sweet11-2*) were described previously^18,25^. The knockout mutants *sweet13-1*, *sweet13-2*, *sweet14-1* and *sweet14-2* were obtained with a CRISPR–Cas9 construct targeting coding sequences 5′-GCCTGTCCCTGCAGCATCCCTGG-3′ of *SWEET13* and 5′-GCATGTCTCTTCAGCATCCCTGG-3′ of *SWEET14* (where underlining indicates the position of the protospacer-adjacent motif (PAM)) common to the first exon as previously described^18^. Double mutants (*sweet13-2*;*sweet14-1*) were created by crossing. *SWEET13* RNA levels were analyzed in the *sweet13* mutant and shown to be reduced (Fig. 3b).

#### Plant materials and growth conditions

Kitaake wild-type and mutant plants were grown either in field conditions (20 individual plants per genotype, paddy field in summer 2016, Carnegie) or in greenhouses under long-day conditions of 14-h day/10-h night at 28–30 °C and 50% relative humidity, with a light intensity of 500–1,000 µmol m^–2^ s^–1^. Independent transformants for the GUS fusions were characterized (transcriptional GUS fusions: *SWEET11*, *n* = 8; *SWEET13*, *n* = 8; *SWEET14*, *n* = 15; translational GUS fusions: *SWEET11*, *n* = 18; *SWEET13*, *n* = 9; *SWEET14*, *n* = 29). All independent transformants showed similar tissue specificity by histochemical staining. Analyses were performed for a minimum of six plants per infection of each *Xoo* strain. Three independent experiments were performed for specific induction in a growth chamber (12-h day at 28 °C/12-h night at 25 °C, 80% relative humidity). IR64 lines were grown in a small-scale field environment in a screenhouse (28 ± 7 °C during the day/23 ± 4 °C at night; 80–85% relative humidity) at IRRI.

#### Histochemical GUS analyses

Samples were collected in cold 90% acetone for fixation, vacuum infiltrated for 10 min and incubated for 30 min at room temperature. Leaf samples were vacuum infiltrated in GUS washing buffer (staining solution without 5-bromo-4-chloro-3-indole-b-glucuronide (X-Gluc)) on ice for 10 min. The solution was changed to GUS staining solution (50 mM sodium phosphate pH 7.0, 10 mM EDTA, 20% (vol/vol) methanol, 0.1% (vol/vol) Triton X-100, 1 mM potassium ferrocyanide, 1 mM potassium ferricyanide and 2 mM X-Gluc dissolved in DMSO). Samples were incubated at 37 °C. After 2 h of incubation, samples were cleared in an ethanol series (20%, 35% and 50%) at room temperature for 30 min. Samples from *Xoo-*inoculated leaves were incubated in 70% ethanol to remove the chlorophyll. Specimens were observed with a SteREO Discovery.V12 stereoscope (Zeiss). For paraffin sections, samples were fixed using FAA for 30 min (50% (vol/vol) ethanol, 3.7% (vol/vol) formaldehyde and 5% (vol/vol) acetic acid). Dehydration was performed with an ethanol series (70%, 80%, 90% and 100%, 30 min each) and 100% *tert*-butanol. Samples were transferred and embedded in Histosec pastilles (Millipore). Sections (10 μm) were obtained with a rotary microtome (Jung RM 2025). Specimens were observed with an Eclipse e600 microscope (Nikon). GUS histochemistry experiments were performed at least two times with 12 individual plants, with similar results.

#### *Xoo* strains and infection protocols

The *Xoo* strains collected from different geographic regions were reported^24^. Plasmid-containing *Xoo* strains were obtained through electroporation of competent cells^46^ with respective pHM1-derived plasmids (e.g., pHM1/ZWpthXo1 for the *pthXo1* gene)^47^. For infection experiments, bacterial inocula were prepared by growing bacterial cells on tryptone sucrose plates with appropriate antibiotics. Cells were scraped from the plates and resuspended in sterile distilled water at OD_600_ ~ 0.5. (i) Leaf clipping^48^: the two youngest fully expanded leaves of 4- to 5-week-old rice plants were clipped about 1–2 cm from the tip with scissor blades that were immersed in bacteria immediately before clipping. Five plants were used for inoculation of each strain (ten leaves in total). Lesion length (distance from the cut to the leading edge of (gray) symptoms) was measured for each inoculated leaf at 12–14 DAI. The mean lesion length of ten leaves was used for each treatment. The Tukey test for analysis after ANOVA was used for statistical analyses. Leaf tissues were mounted in laminating film and photographed under white light. (ii) Syringe infiltration: bacterial suspensions were infiltrated into leaves from the bottom by pressing the opening of a needleless syringe to the leaf. Leaf fragments with inoculated spots were cut off 48 h after inoculation for RNA extraction and GUS staining analysis.

### Statistical analysis

Data were plotted using BoxPlotR (http://shiny.chemgrid.org/boxplotr/) or are presented as mean ± s.e.m as specified in respective tables or figures. One-sided ANOVA was conducted on measurements. The Tukey honestly significant difference test was used after ANOVA pairwise tests for significance, which was set at *P* t-test was used. Exact *P* values, the statistical test used and sample number *n* can be found in figure legends or graphs.

### Reporting Summary

Further information on research design is available in the Nature Research Reporting Summary linked to this article.

## Online content

Any methods, additional references, Nature Research reporting summaries, source data, statements of code and data availability and associated accession codes are available at 10.1038/s41587-019-0268-y.

## Supplementary Information

### Integrated supplementary information

Supplementary Figure 1 Alignment of the *SWEET11* promoter sequences from selected rice varieties. Rice varieties having nucleotide variations in the PthXo1 EBE were identified using RiceVarMap v.2 (http://ricevarmap.ncpgr.cn/v2/). Two varieties were selected for each variation type as representative. Sequences of the first 400 bp of *SWEET11* promoters of the selected varieties were extracted from the 3K database (http://snp-seek.irri.org/). Alignment was done using ClustalW (v 2.1) in Geneious 11.1.5 (https://www.geneious.com*)*. One A/G variation was found in the PthXo1 EBE. Variation was observed with a frequency of 0.2% in 4,726 rice varieties.

Supplementary Figure 2 Alignment of the *SWEET13* promoter sequences from selected rice varieties. Rice varieties having nucleotide variations in the PthXo2 EBE were identified using RiceVarMap v.2 (http://ricevarmap.ncpgr.cn/v2/). Two varieties were selected for each variation type as representative. Sequences of the first 400 bp of *SWEET13* promoters of the selected varieties were extracted from the 3K database (http://snp-seek.irri.org/). Alignment was done using ClustalW in Geneious 11.1.5 (https://www.geneious.com*)*. Nine variations were found in the PthXo2 EBE with frequencies ranging from 1.3% to 20.8%.

Supplementary Figure 3 Alignment of the *SWEET14* promoter sequences from selected rice varieties. Rice varieties having nucleotide variations in the PthXo3, TalC, AvrXa7 and TalF EBEs were identified using RiceVarMap v.2 (http://ricevarmap.ncpgr.cn/v2/). Two varieties were selected for each variation type as representative. Sequences of the first 400 bp of *SWEET14* promoters of the selected varieties were extracted from the 3K database (http://snp-seek.irri.org/). Alignment was done using ClustalW in Geneious 11.1.5 (https://www.geneious.com*)*. In the PthXo3/AvrXa7 EBEs, there was one A insertion with a frequency of 7.7%. CX371 and CX372 had one G/T variation in the TalC EBE and an 18-bp deletion in the PthXo3/AvrXa7 and TalF EBEs.

Supplementary Figure 4 *SWEET* mRNA levels in uninfected rice leaves. **a**, Relative mRNA levels (qRT-PCR) of *SWEET11*, *SWEET13*, *SWEET14* and *SWEET15* in different regions of rice flag leaves. Samples were harvested at 12 pm (mean ± s.e.m., *n* = 3 leaf samples from siblings grown in parallel) with expression normalized to rice *Ubiquitin1* levels. This experiment was repeated independently three times with similar results). **b**, Tissue-specific expression pattern of *SWEET13* from public microarray data (http://ricexpro.dna.affrc.go.jp).

Supplementary Figure 5 Transcriptional fusion reporter lines for SWEET11, 13 and 14. GUS staining patterns of SWEET11, 13 and 14 transcriptional GUS fusion lines. Transcriptional GUS fusion lines show a non-specific expression pattern in leaf tissues. **a**, SWEET11 transcriptional GUS fusion lines. **b**, SWEET13 transcriptional GUS fusion lines. **c**, SWEET14 transcriptional GUS fusion lines. Scale bar, 1 mm. This experiment was repeated independently at least three times (*n* = 3 leaf samples from siblings grown in parallel) with similar results.

Supplementary Figure 6 Map of the SWEET11 translational reporter fusion constructs.

Supplementary Figure 7 Map of the SWEET13 translational reporter fusion constructs.

Supplementary Figure 8 Map of the SWEET14 translational reporter fusion constructs.

Supplementary Figure 9 SWEET protein accumulation in rice leaves infected with *Xoo* strains expressing a specific TALe. SWEET protein accumulation upon infection with *Xoo-*containing specific TAL effectors. Translational GUS fusion lines were infected with an ME2 strain harboring a specific effector. SWEET11 was induced upon inoculation with ME2 expressing the PthXo1 effector. SWEET13 was induced by ME2 with the PthXo2B effector. SWEET14 was induced by ME2 with PthXo3, AvrXa7, TalC or TalF. This experiment was repeated independently twice with similar results.

Supplementary Figure 10 CRISPR-Cas9 editing of *SWEET13* and *SWEET14* for knock-out lines and predicted truncated form of transporters. Mutagenesis of *SWEET13* and *SWEET14* using CRISPR/Cas9 genome editing. The guide RNA-targeting site is marked with an underline, and the PAM is marked in green. **a**, Mutagenesis scheme of *SWEET13* and *SWEET14*. The dashed line denotes a deleted nucleotide in *sweet13-1* (10 nt), *sweet13-2* (4 nt) and *sweet14-1* (1 nt), respectively. 1nt insertion in *sweet14-2* is marked in blue. Both deletion and frameshift of amino acids occurred in the 1^st^ exon and caused early termination. **b**, Predicted amino acid sequence of *sweet13-1, sweet13-2*, *sweet14-1* and *sweet14-2*, respectively. In *sweet13-1* and *sweet13-2*, frameshifts occured at the position of codons 8 and 7 of the original open reading frame, respectively, leading to polypeptides with altered sequence and length due to premature stop codons. **c**, If we assume that the second ATG (codon 58 in wild-type *SWEET13*) was used for protein production, only truncated proteins could be formed. In both mutants, the mutations will lead to loss of the first two transmembrane spanning domains, most likely leading to non-functional transporters. **d**, Predicted topology of the truncated *SWEET14* protein in the *sweet14-1* and *sweet14-2* mutants in case codon 23 would serve a start codon. In both mutants, the mutations will lead to loss of the first transmembrane-spanning domain, most likely leading to non-functional transporters. Typically, premature stop codons affect RNA stability. Moreover, typically only the first ATG is used; thus, it is likely that all four lines completely lost the transport functions for the respective *SWEETs*.

Supplementary Figure 11 Sucrose transport activity and subcellular localization of SWEET13 from rice. **a**, Sucrose transport activity by SWEET13 in HEK293T cells co-expressing the FLIPsuc90μ∆1V sucrose sensor. Cells expressing the sensor without SWEET13 were used as negative controls. SWEET14 served as a positive control (mean ± s.e.m.). **b**, Confocal Z-stack of *Agrobacterium*-infiltrated *N. benthamiana* epidermal leaf cells. ZmSWEET13a-eGFP fluorescence indicated localization at the plasma membrane. The eGFP signal (green) was merged with fluorescence derived from chloroplasts (667–773 nm) (blue). Both experiments were repeated at least three times independently. Scale bar, 50 µm.

Supplementary Figure 12 Molecular and phenotypic characterization of alleles of *sweet13* mutants. **a**, Number of seeds per panicle of greenhouse-grown wild-type *sweet13-1* and *-2* lines grown side by side (*n* = 4). No significant differences (*P* = 0.131 for *sweet13-1* and *P* = 0.054 for *sweet13-*2) were observed with Student’s *t*-test. **b**, Total soluble sugars in wild-type and *sweet13-1* and *-2* flag leaves. Both mutants showed similar sugar concentrations compared to wild type. Samples were harvested at dusk (8 pm; *n* = 4 leaf samples from siblings grown in parallel and repeated independently at least three times, with similar results). Data were plotted using BoxPlotR (http://shiny.chemgrid.org/boxplotr/). Center lines show medians; box limits indicate the 25th and 75th percentiles as determined by *R* software; and data points were plotted as open circles. No significant differences were observed with Student’s *t*-test (*sweet13-1*: *P* = 0.318 for sucrose, *P* = 0.170 for glucose and *P* = 0.242 for fructose; *sweet13-2*: *P* = 0.824 for sucrose, *P* = 0.573 for glucose and *P* = 0.882 for fructose).

Supplementary Figure 13 mRNA levels of clade III *SWEETs* in *sweet13* mutants. **a–c**, Relative mRNA levels (2^–ΔΔCt^) of *SWEET11*, *SWEET14* and *SWEET15* in the *sweet13-1* mutant (wild-type control: Kitaake). *SWEET14* was the only *SWEET* clade III that showed significant upregulation in the mutant (mean ± s.e.m., *n* = 2 leaf samples from siblings grown in parallel, with mRNA levels normalized to the rice *Ubiquitin1* levels, and repeated independently three times, with similar results). **d**, *SWEET14* mRNA levels in the region around the laminar joint of the flag leaf (~1 cm of flag leaf blade base region plus laminar joint plus ~1 cm of flag leaf sheath top region) of the second allele *sweet13-2* (in blue; wild-type control in white: Kitaake; center lines show the medians; box limits indicate the 25th and 75th percentiles as determined by *R* software; whiskers extend 1.5 times the interquartile range from the 25th and 75th percentiles; outliers are represented by dots; and data points are plotted as open circles; *n* = 4 sample points).

Supplementary Figure 14 SWEET^ko^ knock-out mutants as diagnostic tools. Lesion length caused by ME2 (negative control, black circles, grey box outline), PXO99 (positive control, blue circles, blue box outline) and the African strain AXO1947 (red circles, faint blue boxes) on single, double and triple (*sweet11, sweet13* and *sweet14)* knock-out mutants relative to Kitaake wild type. Lesion lengths measured at 14 DAI were plotted using BoxPlotR (http://shiny.chemgrid.org/boxplotr/). Center lines show medians; box limits indicate the 25th and 75th percentiles as determined by *R* software; whiskers extend 1.5 times the interquartile range from the 25th and 75th percentiles; and data points are plotted as open circles. Number of tests *n* is indicated below each box on the *x*-axis (numbers between 8 and 23). The same lower letters above the graph bars indicate no significant different at *P* < 0.05 by one-way ANOVA.

Supplementary Figure 15 PathoTracer visualization under the SWEET^R^ kit 1.0 showing prevalence of *Xoo* strains with putative *SWEET14* induction in the Philippines. PathoTracer (http://webapps.irri.org/pathotracer/site2/) is an online repository that integrates genotypic and phenotypic pathogen data with resistance profiles of rice accessions to support the strategic deployment of varieties in the region. Highlighted in this figure is the population of *Xoo* collected from 1972 to 2012 in Laguna, a BB-disease endemic area in the Philippines (*n* = 1,294 isolates). A screenshot of the same map is shown in Fig. 6. The righthand side panel displays detailed information on the population structure of *Xoo* in that region in relation to putative induction of *SWEET14* (*n* = 1,294 isolates) and any recommended varieties.

### Supplementary Information

Supplementary Information Supplementary Figs. 1–15, Supplementary Tables 1–11 and Supplementary Notes 1–3

Reporting Summary

## Data Availability

Materials will be made available for nonprofit research under a material transfer agreement (Supplementary Notes 2 and 3). We aim at obtaining freedom to operate for use by low-income farmers and will work with breeders to make the materials available to subsistence farmers. For commercial applications, accessibility will be negotiated from appropriate patent holders, and profits will be used to support dissemination to subsistence farmers. Edited IR64- and Ciherang-Sub1-based materials can be obtained from R.O.; edited Kitaake lines can be obtained from B.Y.; and translational reporter lines can be obtained from W.B.F. Distribution of *Xoo* strains may be restricted because of regulations of *Xanthomonas oryzae* as a Select Agent by the US government, because of the Nagoya protocol, or because some strains were donated from other groups and thus these groups should be contacted directly (for details, see ref. ^24^).
