# Supplementary figures and images for: Diagnostic kit for rice blight resistance
Source: Nat Biotechnol. 2019 Oct 28;37(11):1372–9. doi: 10.1038/s41587-019-0268-y (PMC6831515; doi:10.1038/s41587-019-0268-y)

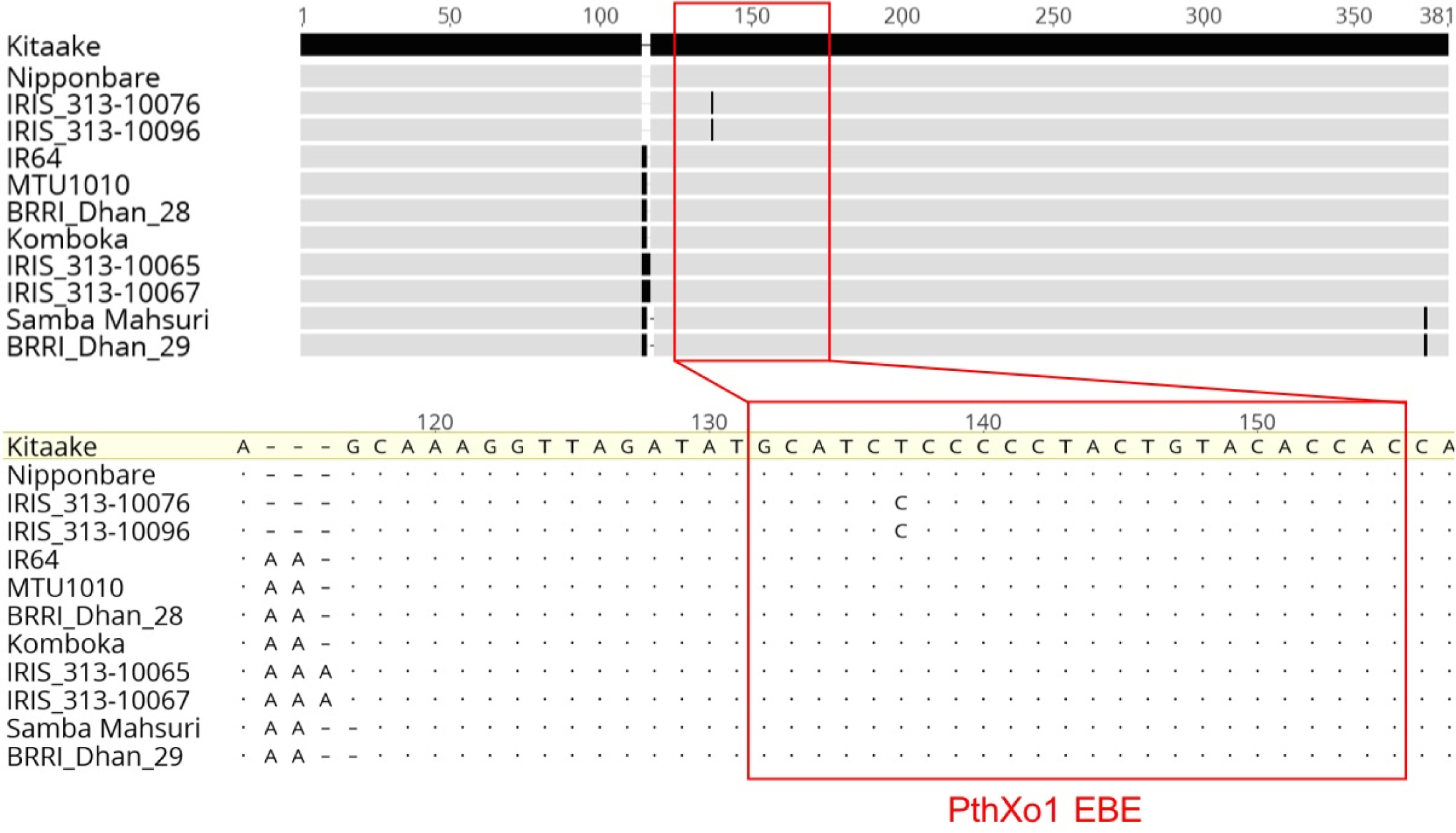

Supplement: Alignment of the SWEET11 promoter sequences from selected rice varieties. — Rice varieties having nucleotide variations in the PthXo1 EBE were identified using RiceVarMap v.2 (http://ricevarmap.ncpgr.cn/v2/). Two varieties were selected for each variation type as representative. Sequences of the first 400 bp of SWEET11 promoters of the selected varieties were extracted from the 3K database (http://snp-seek.irri.org/). Alignment was done using ClustalW (v 2.1) in Geneious 11.1.5 (https://www.geneious.com). One A/G variation was found in the PthXo1 EBE. Variation was observed with a frequency of 0.2% in 4,726 rice varieties. [file 41587_2019_268_Fig7_ESM.jpg]

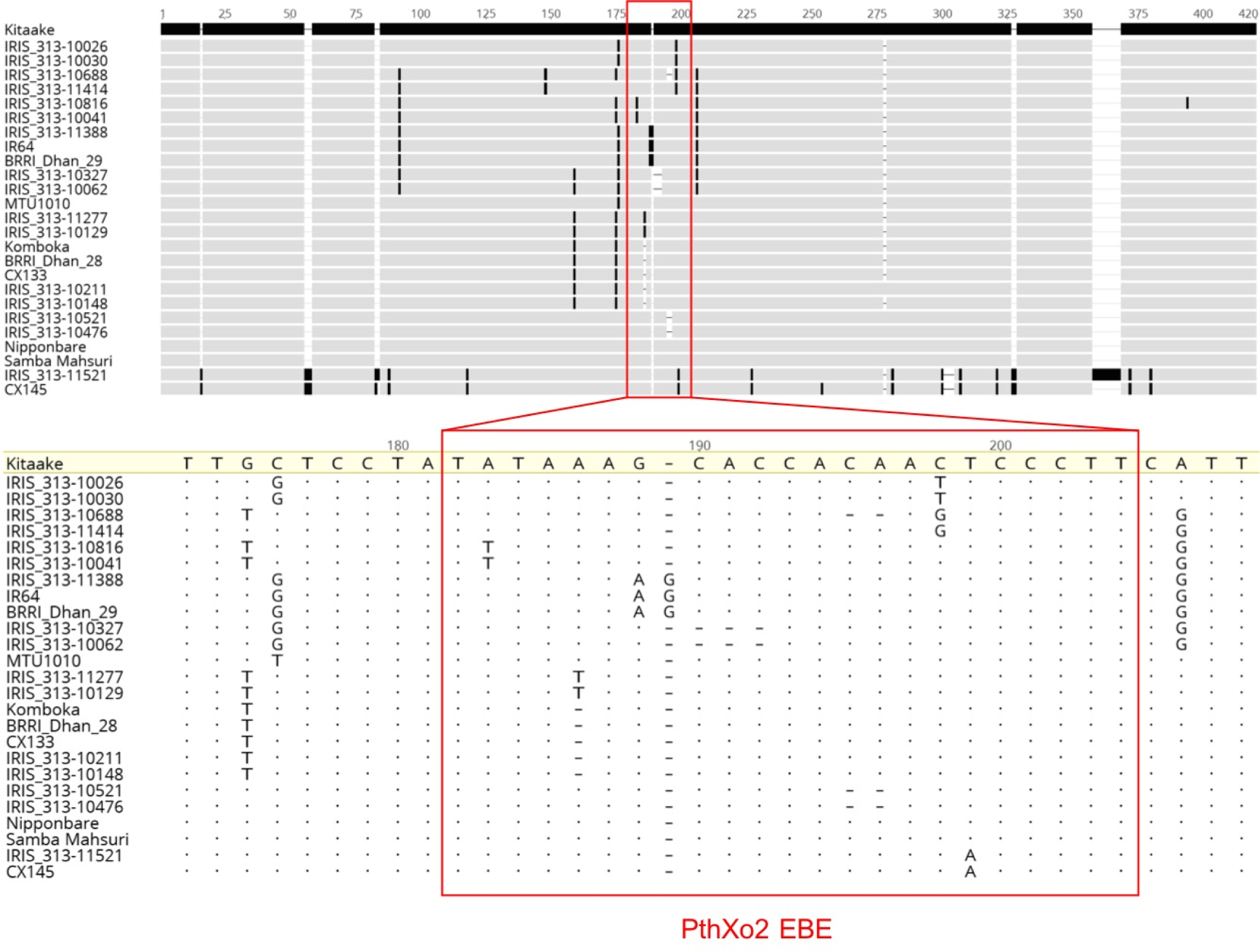

Supplement: Alignment of the SWEET13 promoter sequences from selected rice varieties. — Rice varieties having nucleotide variations in the PthXo2 EBE were identified using RiceVarMap v.2 (http://ricevarmap.ncpgr.cn/v2/). Two varieties were selected for each variation type as representative. Sequences of the first 400 bp of SWEET13 promoters of the selected varieties were extracted from the 3K database (http://snp-seek.irri.org/). Alignment was done using ClustalW in Geneious 11.1.5 (https://www.geneious.com). Nine variations were found in the PthXo2 EBE with frequencies ranging from 1.3% to 20.8%. [file 41587_2019_268_Fig8_ESM.jpg]

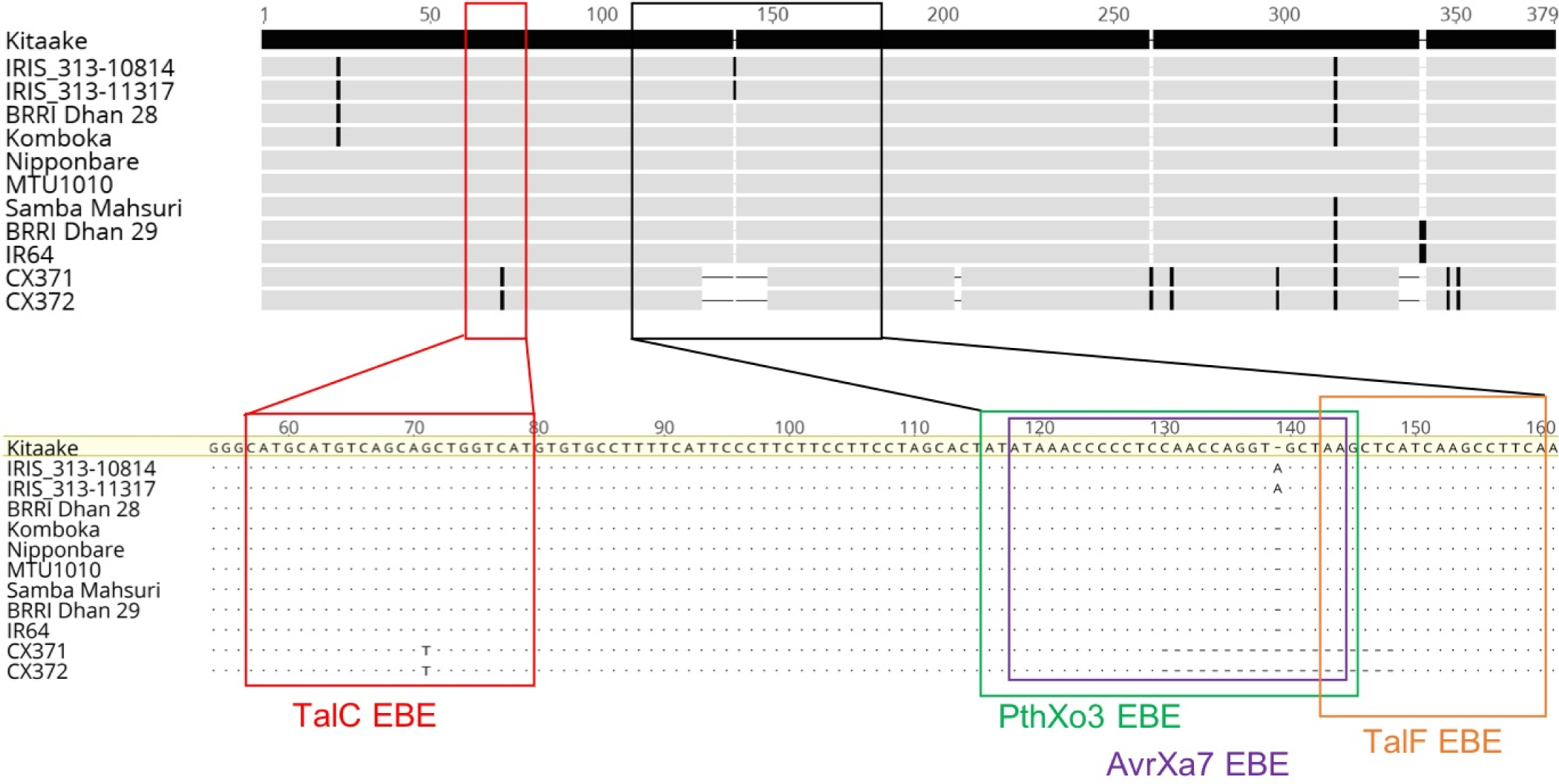

Supplement: Alignment of the SWEET14 promoter sequences from selected rice varieties. — Rice varieties having nucleotide variations in the PthXo3, TalC, AvrXa7 and TalF EBEs were identified using RiceVarMap v.2 (http://ricevarmap.ncpgr.cn/v2/). Two varieties were selected for each variation type as representative. Sequences of the first 400 bp of SWEET14 promoters of the selected varieties were extracted from the 3K database (http://snp-seek.irri.org/). Alignment was done using ClustalW in Geneious 11.1.5 (https://www.geneious.com). In the PthXo3/AvrXa7 EBEs, there was one A insertion with a frequency of 7.7%. CX371 and CX372 had one G/T variation in the TalC EBE and an 18-bp deletion in the PthXo3/AvrXa7 and TalF EBEs. [file 41587_2019_268_Fig9_ESM.jpg]

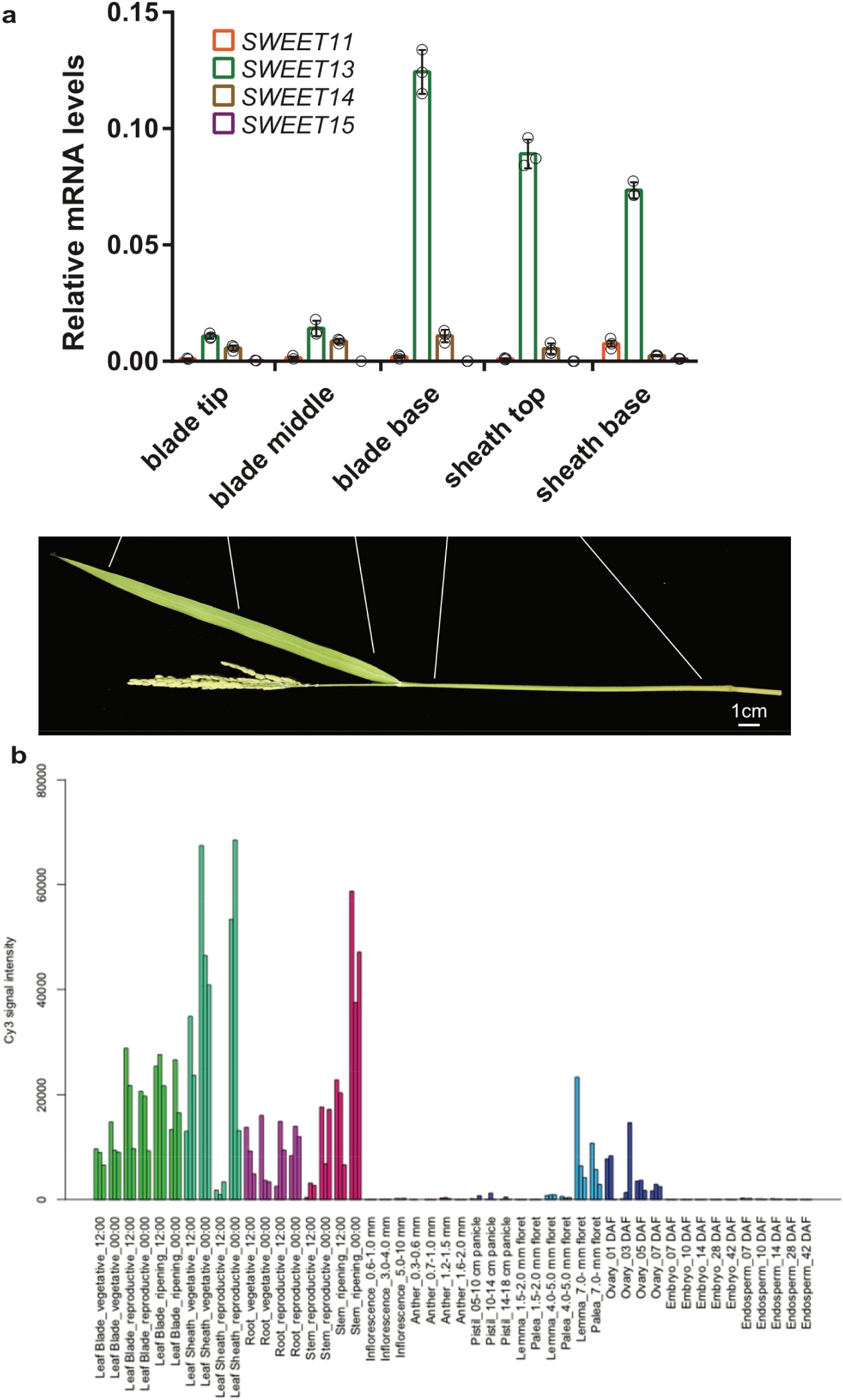

Supplement: SWEET mRNA levels in uninfected rice leaves. — a, Relative mRNA levels (qRT-PCR) of SWEET11, SWEET13, SWEET14 and SWEET15 in different regions of rice flag leaves. Samples were harvested at 12 pm (mean ± s.e.m., n = 3 leaf samples from siblings grown in parallel) with expression normalized to rice Ubiquitin1 levels. This experiment was repeated independently three times with similar results). b, Tissue-specific expression pattern of SWEET13 from public microarray data (http://ricexpro.dna.affrc.go.jp). [file 41587_2019_268_Fig10_ESM.jpg]

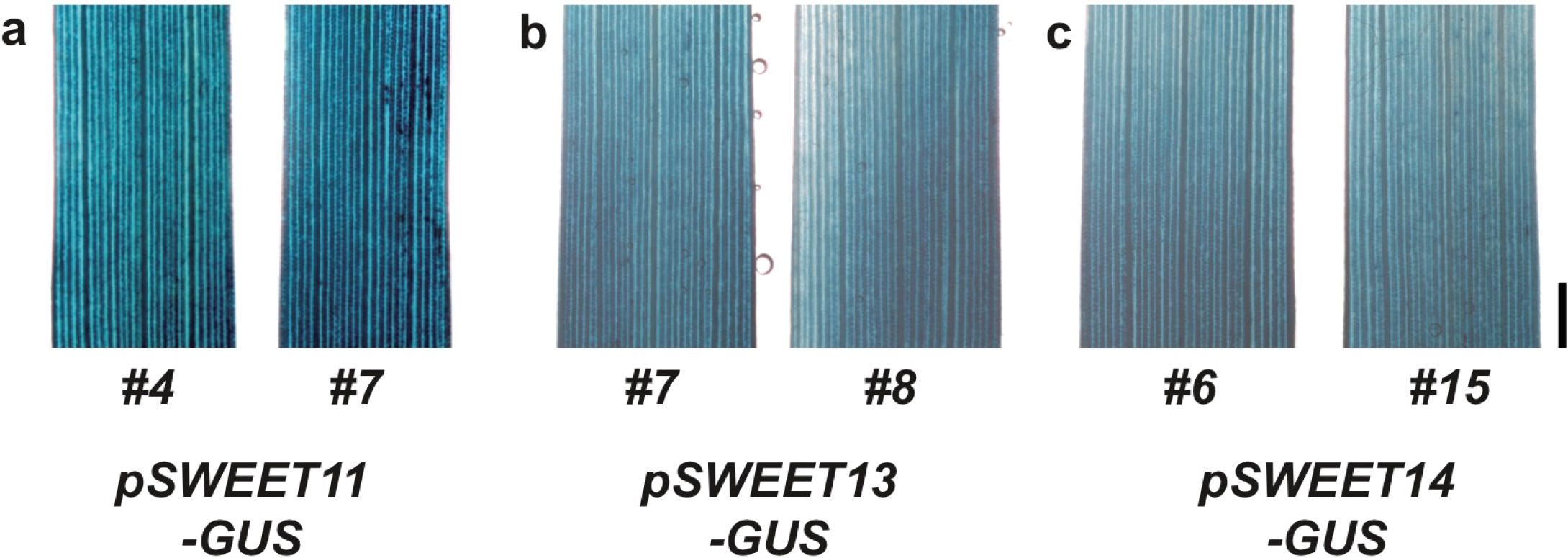

Supplement: Transcriptional fusion reporter lines for SWEET11, 13 and 14. — GUS staining patterns of SWEET11, 13 and 14 transcriptional GUS fusion lines. Transcriptional GUS fusion lines show a non-specific expression pattern in leaf tissues. a, SWEET11 transcriptional GUS fusion lines. b, SWEET13 transcriptional GUS fusion lines. c, SWEET14 transcriptional GUS fusion lines. Scale bar, 1 mm. This experiment was repeated independently at least three times (n = 3 leaf samples from siblings grown in parallel) with similar results. [file 41587_2019_268_Fig11_ESM.jpg]

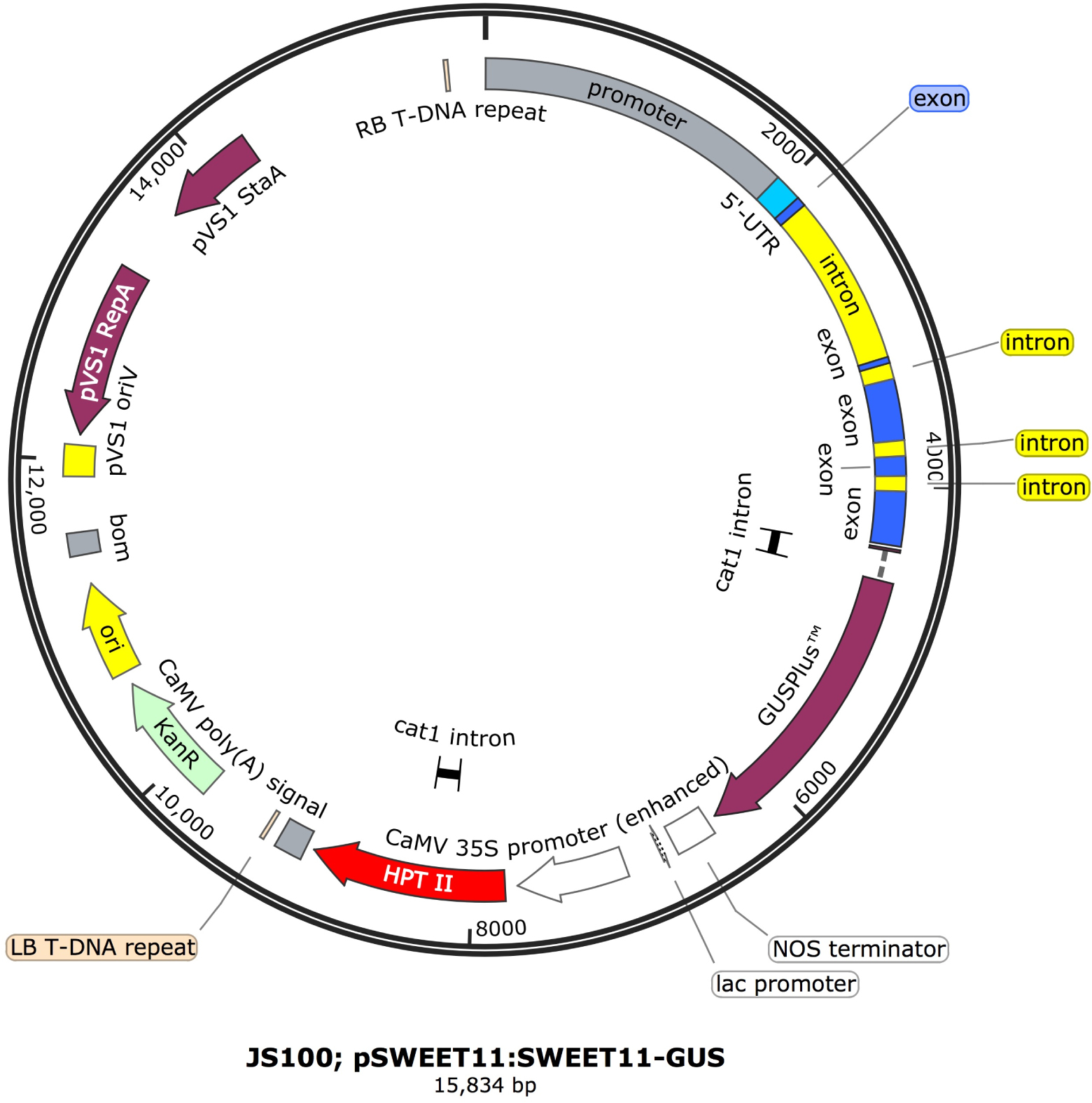

Supplement: Supplementary file 6 — Map of the SWEET11 translational reporter fusion constructs. [file 41587_2019_268_Fig12_ESM.jpg]

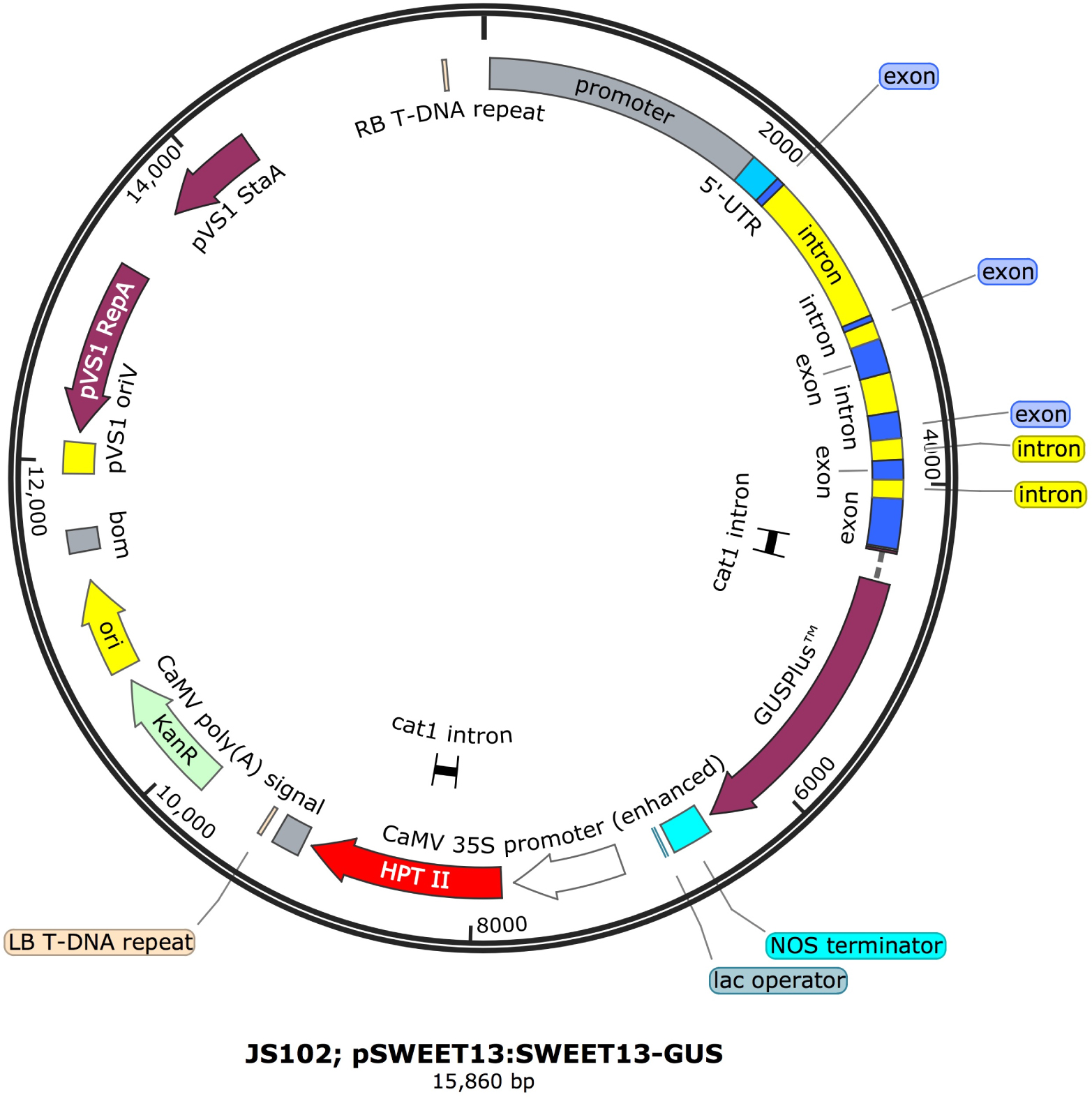

Supplement: Supplementary file 7 — Map of the SWEET13 translational reporter fusion constructs. [file 41587_2019_268_Fig13_ESM.jpg]

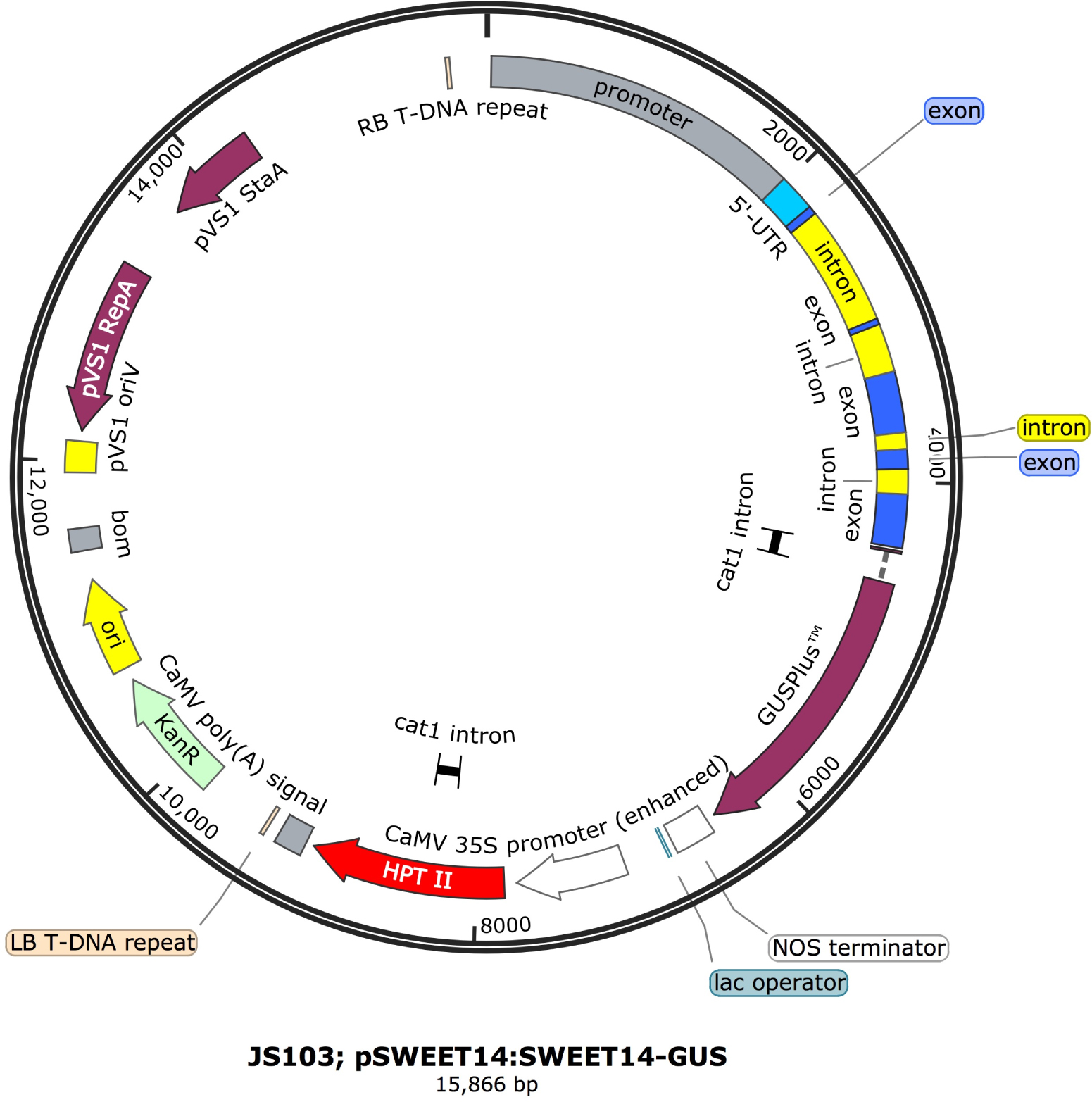

Supplement: Supplementary file 8 — Map of the SWEET14 translational reporter fusion constructs. [file 41587_2019_268_Fig14_ESM.jpg]

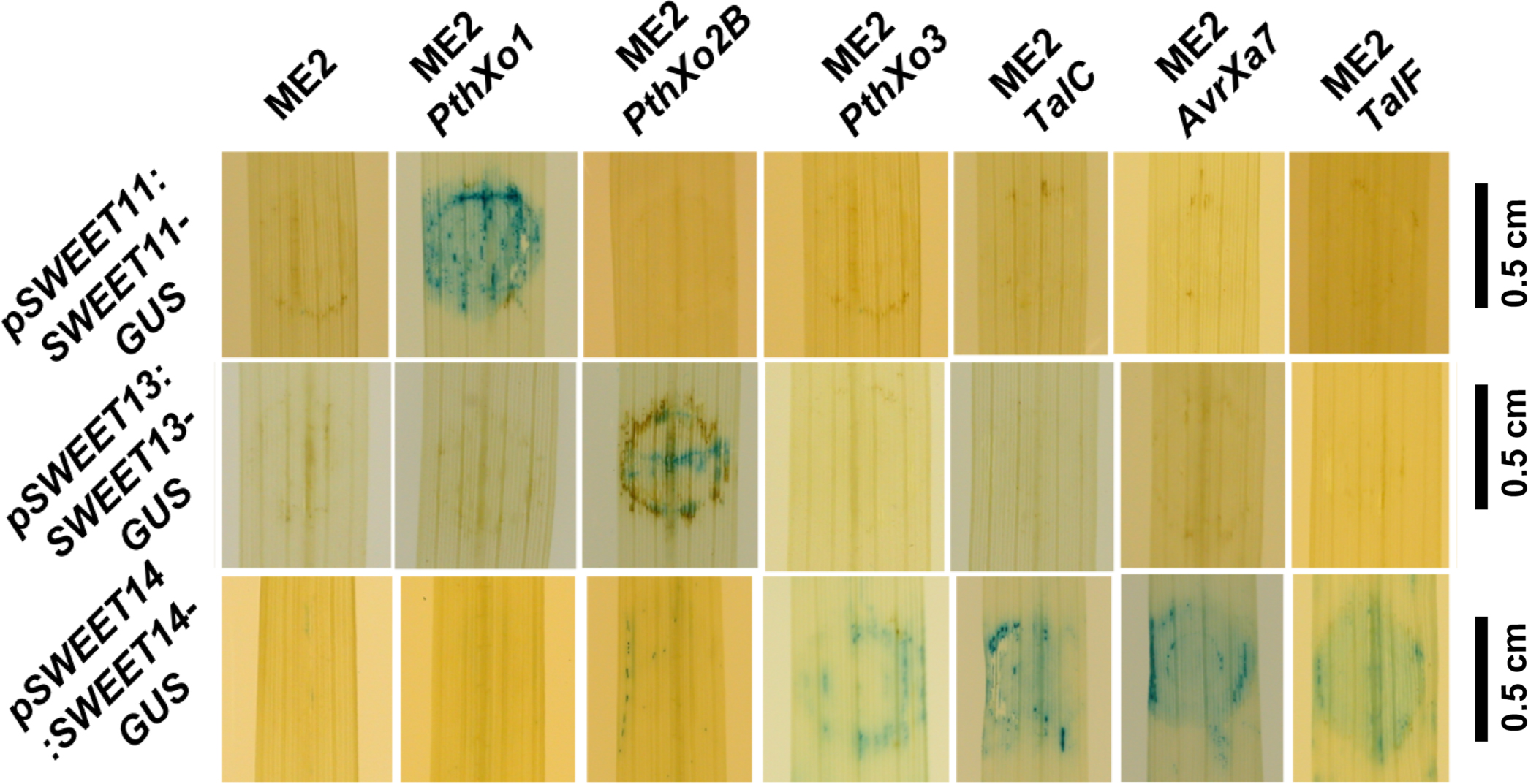

Supplement: SWEET protein accumulation in rice leaves infected with Xoo strains expressing a specific TALe. — SWEET protein accumulation upon infection with Xoo-containing specific TAL effectors. Translational GUS fusion lines were infected with an ME2 strain harboring a specific effector. SWEET11 was induced upon inoculation with ME2 expressing the PthXo1 effector. SWEET13 was induced by ME2 with the PthXo2B effector. SWEET14 was induced by ME2 with PthXo3, AvrXa7, TalC or TalF. This experiment was repeated independently twice with similar results. [file 41587_2019_268_Fig15_ESM.jpg]

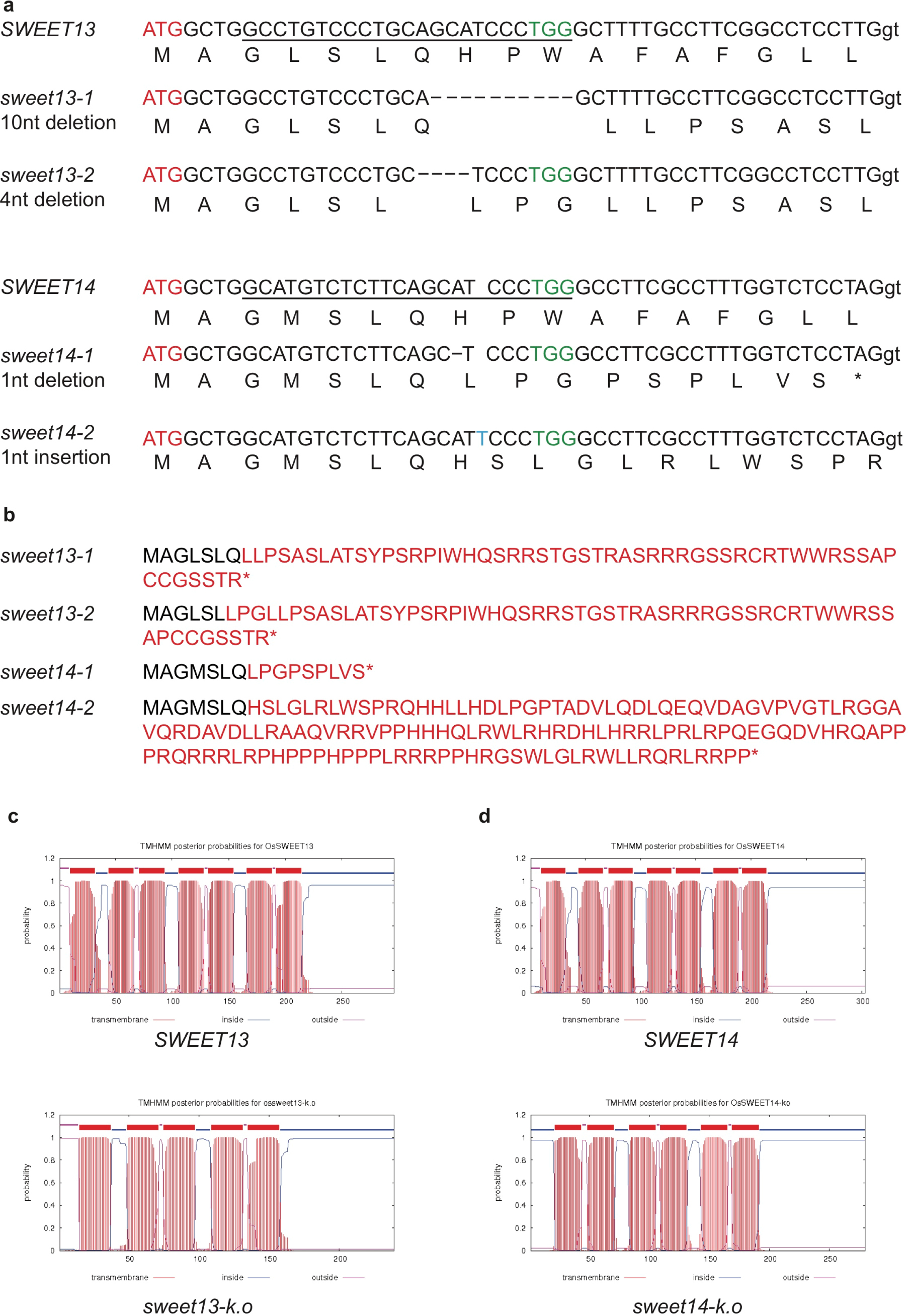

Supplement: CRISPR-Cas9 editing of SWEET13 and SWEET14 for knock-out lines and predicted truncated form of transporters. — Mutagenesis of SWEET13 and SWEET14 using CRISPR/Cas9 genome editing. The guide RNA-targeting site is marked with an underline, and the PAM is marked in green. a, Mutagenesis scheme of SWEET13 and SWEET14. The dashed line denotes a deleted nucleotide in sweet13-1 (10 nt), sweet13-2 (4 nt) and sweet14-1 (1 nt), respectively. 1nt insertion in sweet14-2 is marked in blue. Both deletion and frameshift of amino acids occurred in the 1st exon and caused early termination. b, Predicted amino acid sequence of sweet13-1, sweet13-2, sweet14-1 and sweet14-2, respectively. In sweet13-1 and sweet13-2, frameshifts occured at the position of codons 8 and 7 of the original open reading frame, respectively, leading to polypeptides with altered sequence and length due to premature stop codons. c, If we assume that the second ATG (codon 58 in wild-type SWEET13) was used for protein production, only truncated proteins could be formed. In both mutants, the mutations will lead to loss of the first two transmembrane spanning domains, most likely leading to non-functional transporters. d, Predicted topology of the truncated SWEET14 protein in the sweet14-1 and sweet14-2 mutants in case codon 23 would serve a start codon. In both mutants, the mutations will lead to loss of the first transmembrane-spanning domain, most likely leading to non-functional transporters. Typically, premature stop codons affect RNA stability. Moreover, typically only the first ATG is used; thus, it is likely that all four lines completely lost the transport functions for the respective SWEETs. [file 41587_2019_268_Fig16_ESM.jpg]

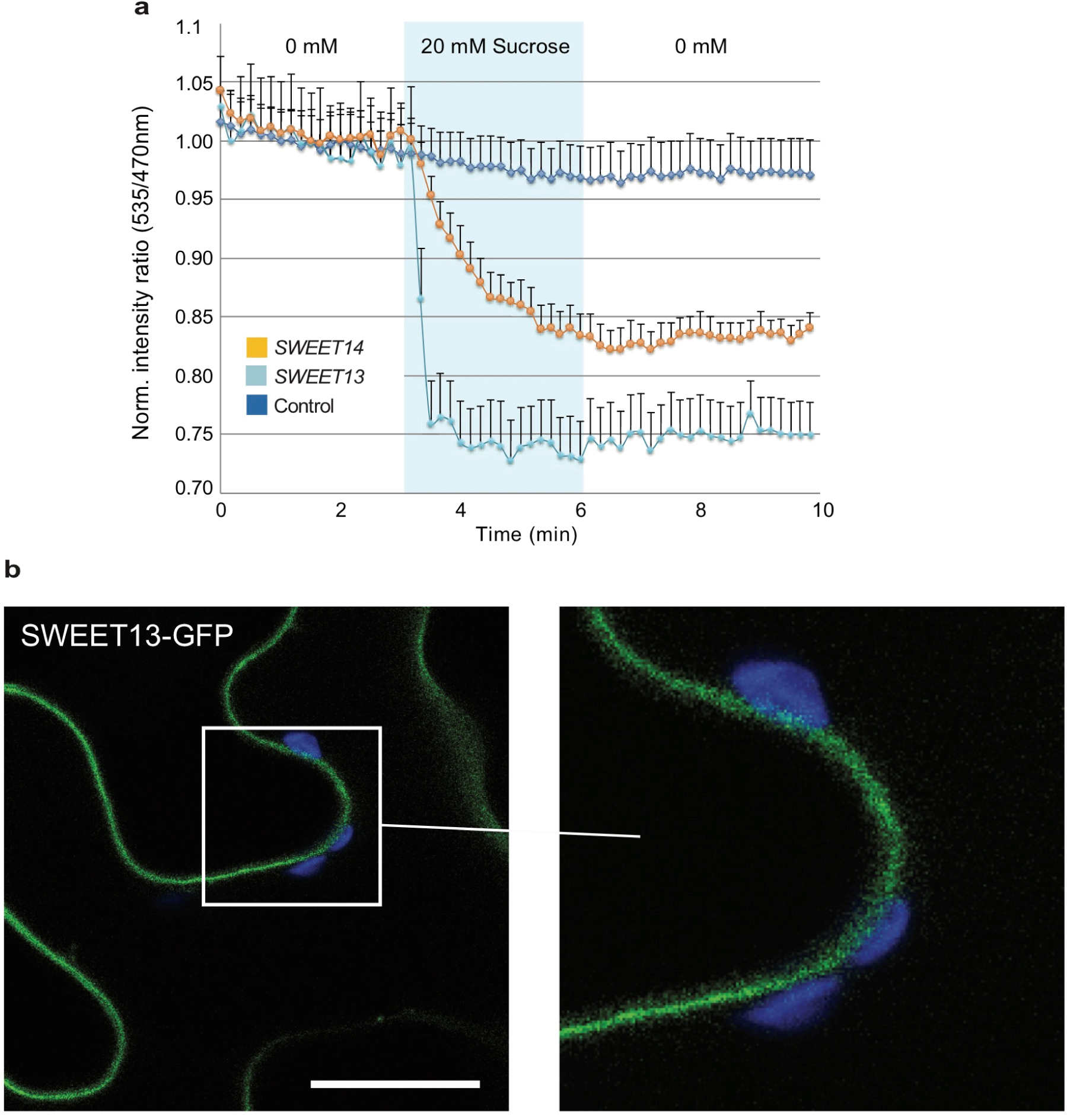

Supplement: Sucrose transport activity and subcellular localization of SWEET13 from rice. — a, Sucrose transport activity by SWEET13 in HEK293T cells co-expressing the FLIPsuc90μ∆1V sucrose sensor. Cells expressing the sensor without SWEET13 were used as negative controls. SWEET14 served as a positive control (mean ± s.e.m.). b, Confocal Z-stack of Agrobacterium-infiltrated N. benthamiana epidermal leaf cells. ZmSWEET13a-eGFP fluorescence indicated localization at the plasma membrane. The eGFP signal (green) was merged with fluorescence derived from chloroplasts (667–773 nm) (blue). Both experiments were repeated at least three times independently. Scale bar, 50 µm. [file 41587_2019_268_Fig17_ESM.jpg]

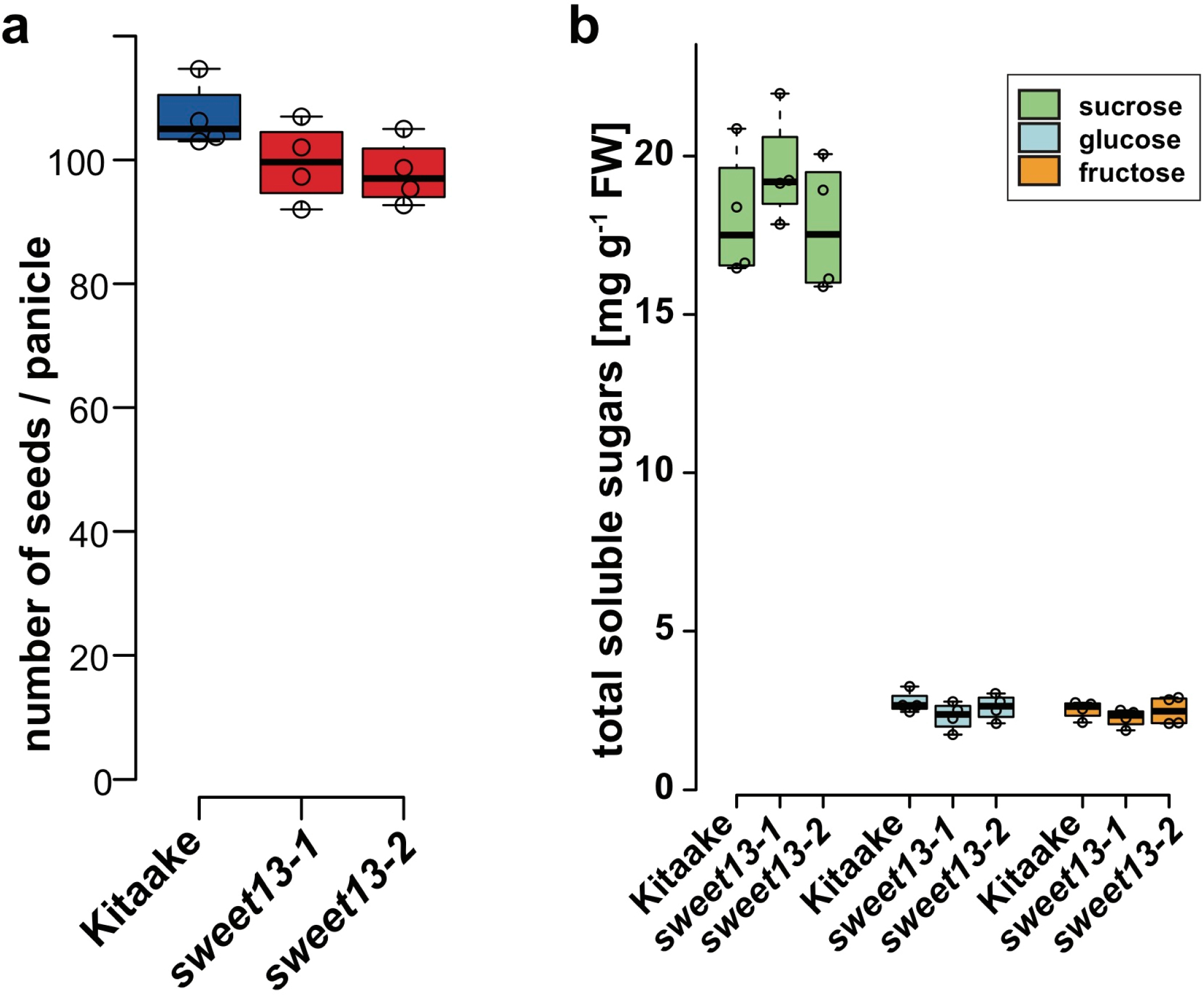

Supplement: Molecular and phenotypic characterization of alleles of sweet13 mutants. — a, Number of seeds per panicle of greenhouse-grown wild-type sweet13-1 and -2 lines grown side by side (n = 4). No significant differences (P = 0.131 for sweet13-1 and P = 0.054 for sweet13-2) were observed with Student’s t-test. b, Total soluble sugars in wild-type and sweet13-1 and -2 flag leaves. Both mutants showed similar sugar concentrations compared to wild type. Samples were harvested at dusk (8 pm; n = 4 leaf samples from siblings grown in parallel and repeated independently at least three times, with similar results). Data were plotted using BoxPlotR (http://shiny.chemgrid.org/boxplotr/). Center lines show medians; box limits indicate the 25th and 75th percentiles as determined by R software; and data points were plotted as open circles. No significant differences were observed with Student’s t-test (sweet13-1: P = 0.318 for sucrose, P = 0.170 for glucose and P = 0.242 for fructose; sweet13-2: P = 0.824 for sucrose, P = 0.573 for glucose and P = 0.882 for fructose). [file 41587_2019_268_Fig18_ESM.jpg]

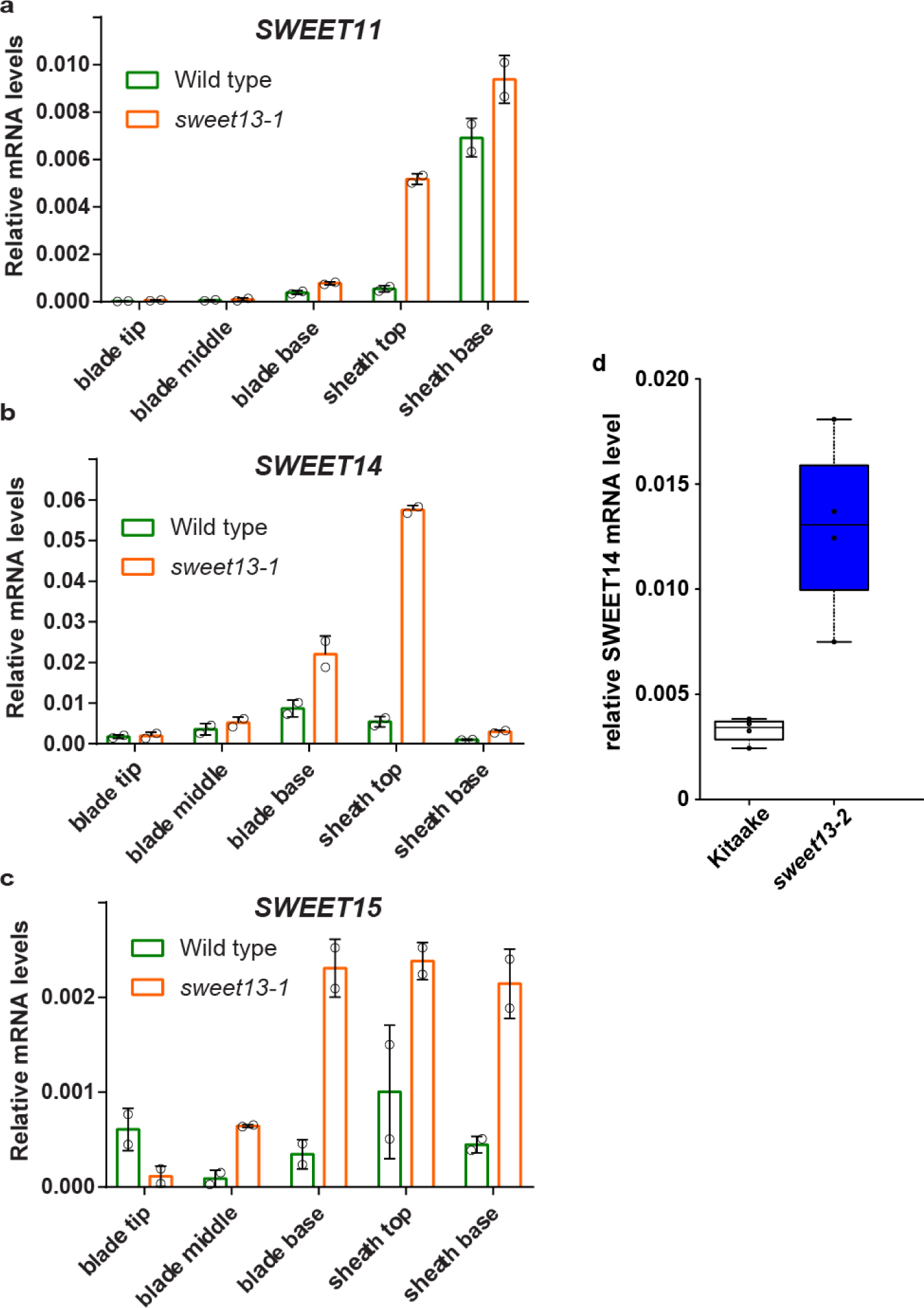

Supplement: mRNA levels of clade III SWEETs in sweet13 mutants. — a–c, Relative mRNA levels (2–ΔΔCt) of SWEET11, SWEET14 and SWEET15 in the sweet13-1 mutant (wild-type control: Kitaake). SWEET14 was the only SWEET clade III that showed significant upregulation in the mutant (mean ± s.e.m., n = 2 leaf samples from siblings grown in parallel, with mRNA levels normalized to the rice Ubiquitin1 levels, and repeated independently three times, with similar results). d, SWEET14 mRNA levels in the region around the laminar joint of the flag leaf (~1 cm of flag leaf blade base region plus laminar joint plus ~1 cm of flag leaf sheath top region) of the second allele sweet13-2 (in blue; wild-type control in white: Kitaake; center lines show the medians; box limits indicate the 25th and 75th percentiles as determined by R software; whiskers extend 1.5 times the interquartile range from the 25th and 75th percentiles; outliers are represented by dots; and data points are plotted as open circles; n = 4 sample points). [file 41587_2019_268_Fig19_ESM.jpg]

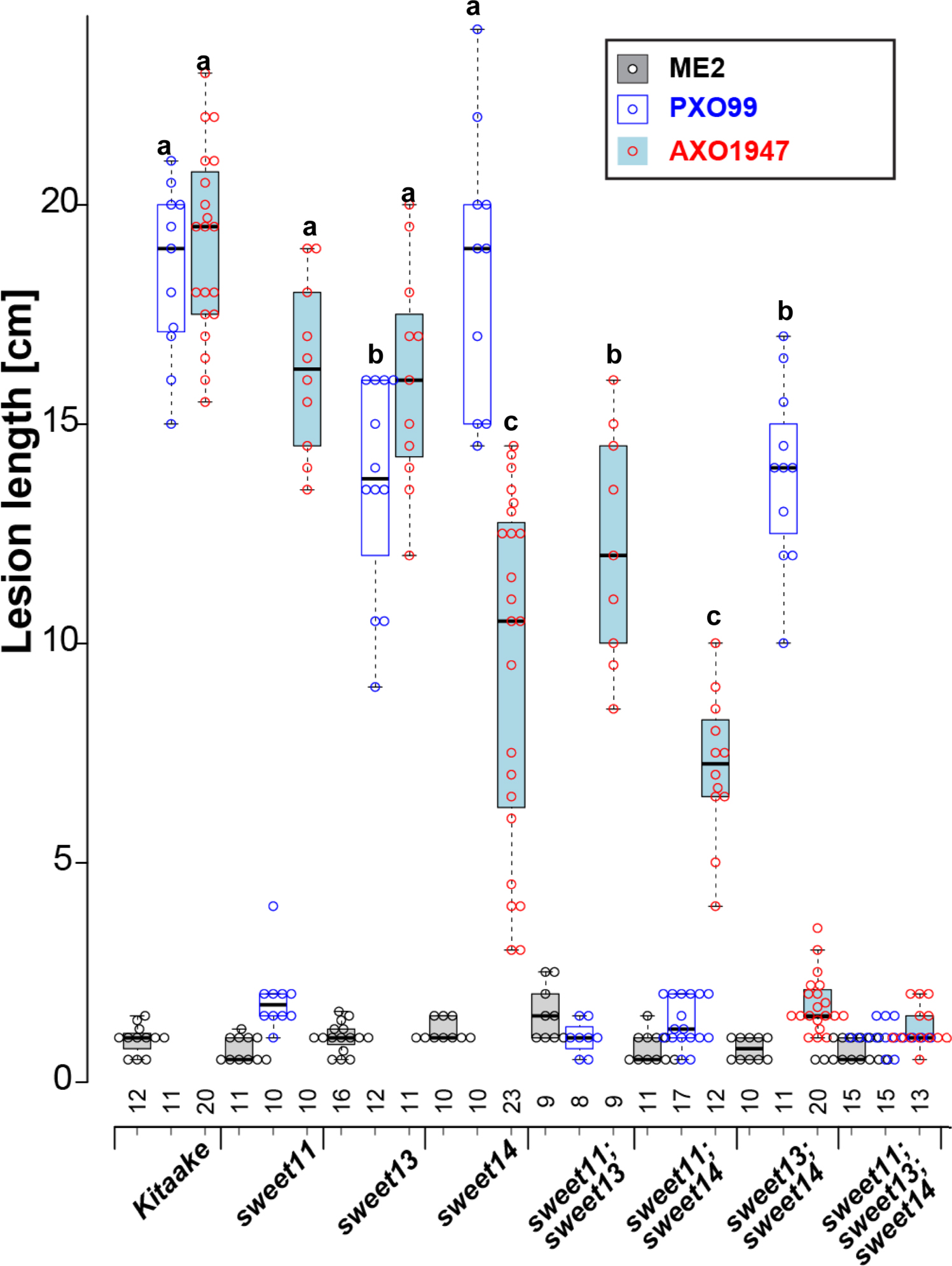

Supplement: SWEETko knock-out mutants as diagnostic tools. — Lesion length caused by ME2 (negative control, black circles, grey box outline), PXO99 (positive control, blue circles, blue box outline) and the African strain AXO1947 (red circles, faint blue boxes) on single, double and triple (sweet11, sweet13 and sweet14) knock-out mutants relative to Kitaake wild type. Lesion lengths measured at 14 DAI were plotted using BoxPlotR (http://shiny.chemgrid.org/boxplotr/). Center lines show medians; box limits indicate the 25th and 75th percentiles as determined by R software; whiskers extend 1.5 times the interquartile range from the 25th and 75th percentiles; and data points are plotted as open circles. Number of tests n is indicated below each box on the x-axis (numbers between 8 and 23). The same lower letters above the graph bars indicate no significant different at P < 0.05 by one-way ANOVA. [file 41587_2019_268_Fig20_ESM.jpg]

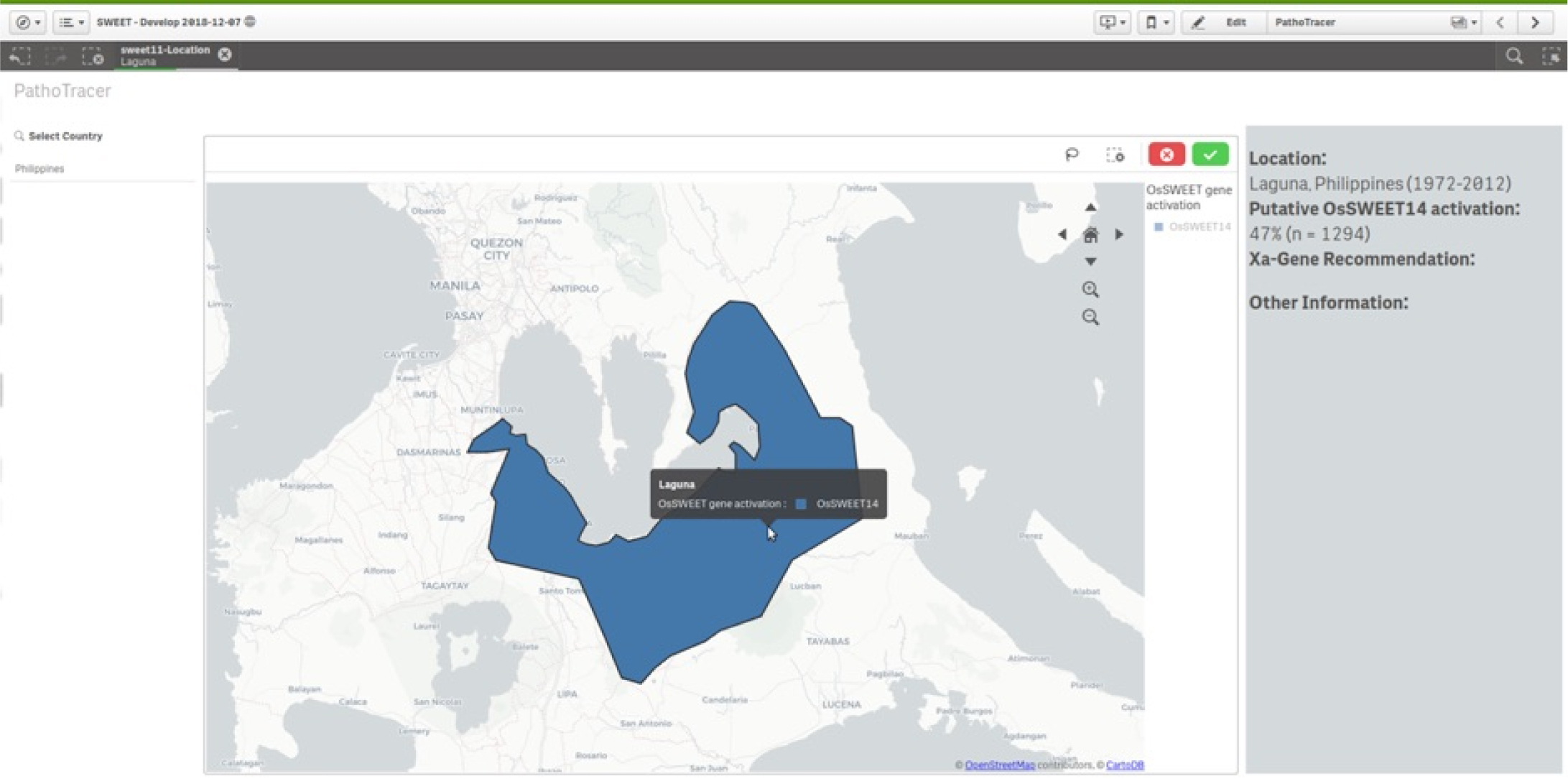

Supplement: PathoTracer visualization under the SWEETR kit 1.0 showing prevalence of Xoo strains with putative SWEET14 induction in the Philippines. — PathoTracer (http://webapps.irri.org/pathotracer/site2/) is an online repository that integrates genotypic and phenotypic pathogen data with resistance profiles of rice accessions to support the strategic deployment of varieties in the region. Highlighted in this figure is the population of Xoo collected from 1972 to 2012 in Laguna, a BB-disease endemic area in the Philippines (n = 1,294 isolates). A screenshot of the same map is shown in Fig. 6. The righthand side panel displays detailed information on the population structure of Xoo in that region in relation to putative induction of SWEET14 (n = 1,294 isolates) and any recommended varieties. [file 41587_2019_268_Fig21_ESM.jpg]
