# Supplementary material for: Diagnostic kit for rice blight resistance
Source: Nat Biotechnol. 2019 Oct 28;37(11):1372–9. doi: 10.1038/s41587-019-0268-y (PMC6831515; doi:10.1038/s41587-019-0268-y)
Supplement: Supplementary file 16 — Supplementary Figs. 1–15, Supplementary Tables 1–11 and Supplementary Notes 1–3 [file 41587_2019_268_MOESM1_ESM.pdf]

nature biotechnology

SUPPLEMENTARY INFORMATION

https://doi.org/10.1038/s41587-019-0268-y

In the format provided by the authors and unedited.

Diagnostic kit for rice blight resistance

Joon-Seob Eom[1,2,11], Dangping Luo[3,11], Genelou Atienza-Grande[4,10], Jungil Yang[1,2], Chonghui Ji[3], Van Thi Luu[1,2], José C. Huguet-Tapia[5], Si Nian Char[6], Bo Liu[3], Hanna Nguyen[4], Sarah Maria Schmidt[1,2], Boris Szurek[7], Casiana Vera Cruz[4], Frank F. White[5], Ricardo Oliva[4], Bing Yang[3,8]* and Wolf B. Frommer[1,2,9]*

[1]Institute for Molecular Physiology and Cluster of Excellence on Plant Sciences (CEPLAS), Heinrich Heine University of Düsseldorf, Düsseldorf, Germany. [2]Max Planck Institute for Plant Breeding Research, Cologne, Germany. [3]Division of Plant Sciences, Bond Life Sciences Center, University of Missouri, Columbia, MO, USA. [4]International Rice Research Institute, Metro Manila, Philippines. [5]Department of Plant Pathology, University of Florida, Gainesville, FL, USA. [6]Department of Genetics, Development and Cell Biology, Iowa State University, Ames, IA, USA. [7]IRD, CIRAD, Université Montpellier, IPME, Montpellier, France. [8]Donald Danforth Plant Science Center, St. Louis, MO, USA. [9]Institute of Transformative Bio-Molecules (WPI-ITbM), Nagoya University, Aichi, Japan. [10]Present address: College of Agriculture and Food Science, University of the Philippines, Los Baños, Philippines. [11]These authors contributed equally: Joon-Seob Eom, Dangping Luo. *e-mail: yangbi@missouri.edu; frommew@hhu.de

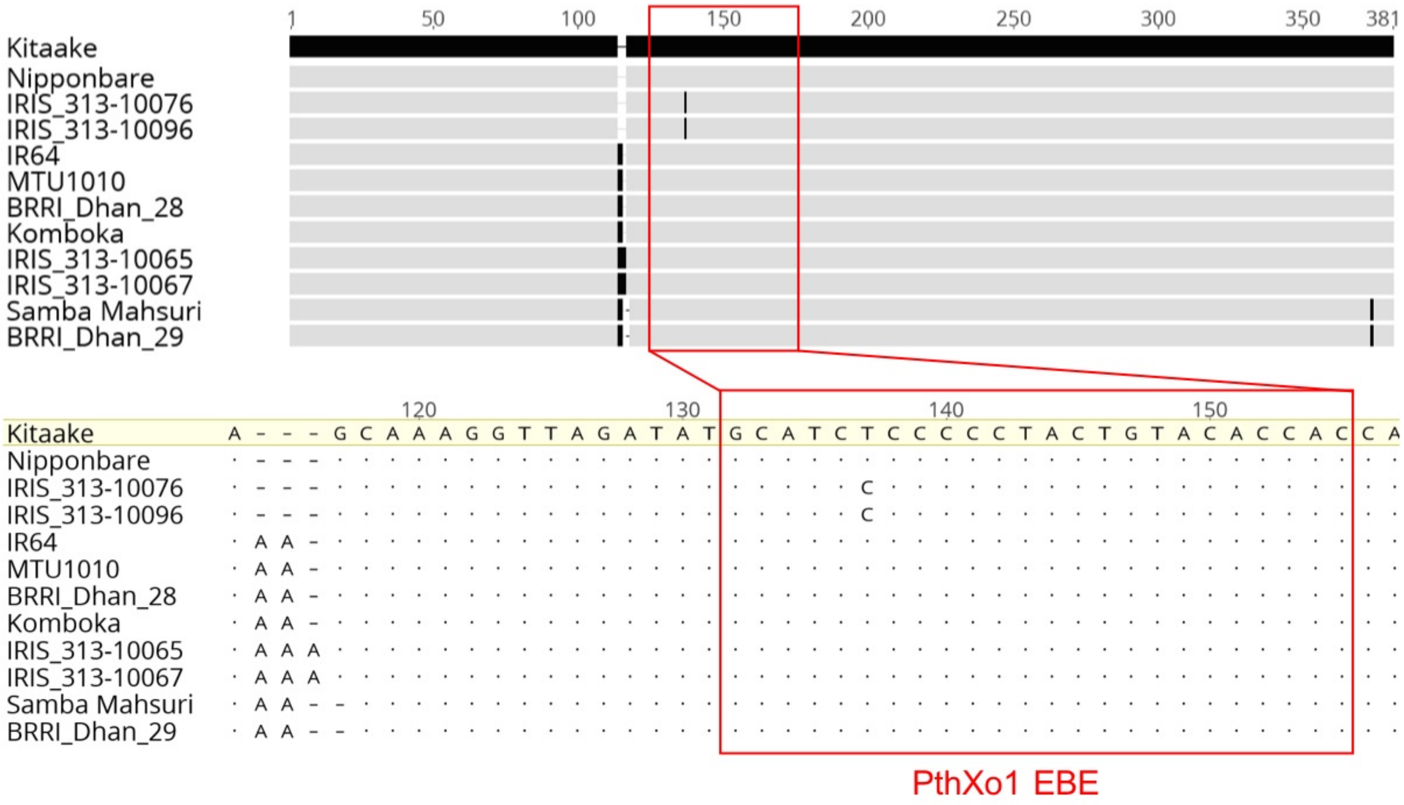

Supplementary Figure 1

Alignment of the *SWEET11* promoter sequences from selected rice varieties.

Rice varieties having nucleotide variations in the PthXo1 EBE were identified using RiceVarMap v.2 (http://ricevarmap.ncpgr.cn/v2/). Two varieties were selected for each variation types as representative. Sequences of the first 400 bp of *SWEET11* promoters of the selected varieties were extracted from the 3K database (http://snp-seek.irri.org/). Alignment was done using ClustalW (v 2.1) in Geneious 11.1.5 (https://www.geneious.com). One A/G variation was found in the PthXo1 EBE. Variation observed with a frequency of 0.2% in 4726 rice varieties.

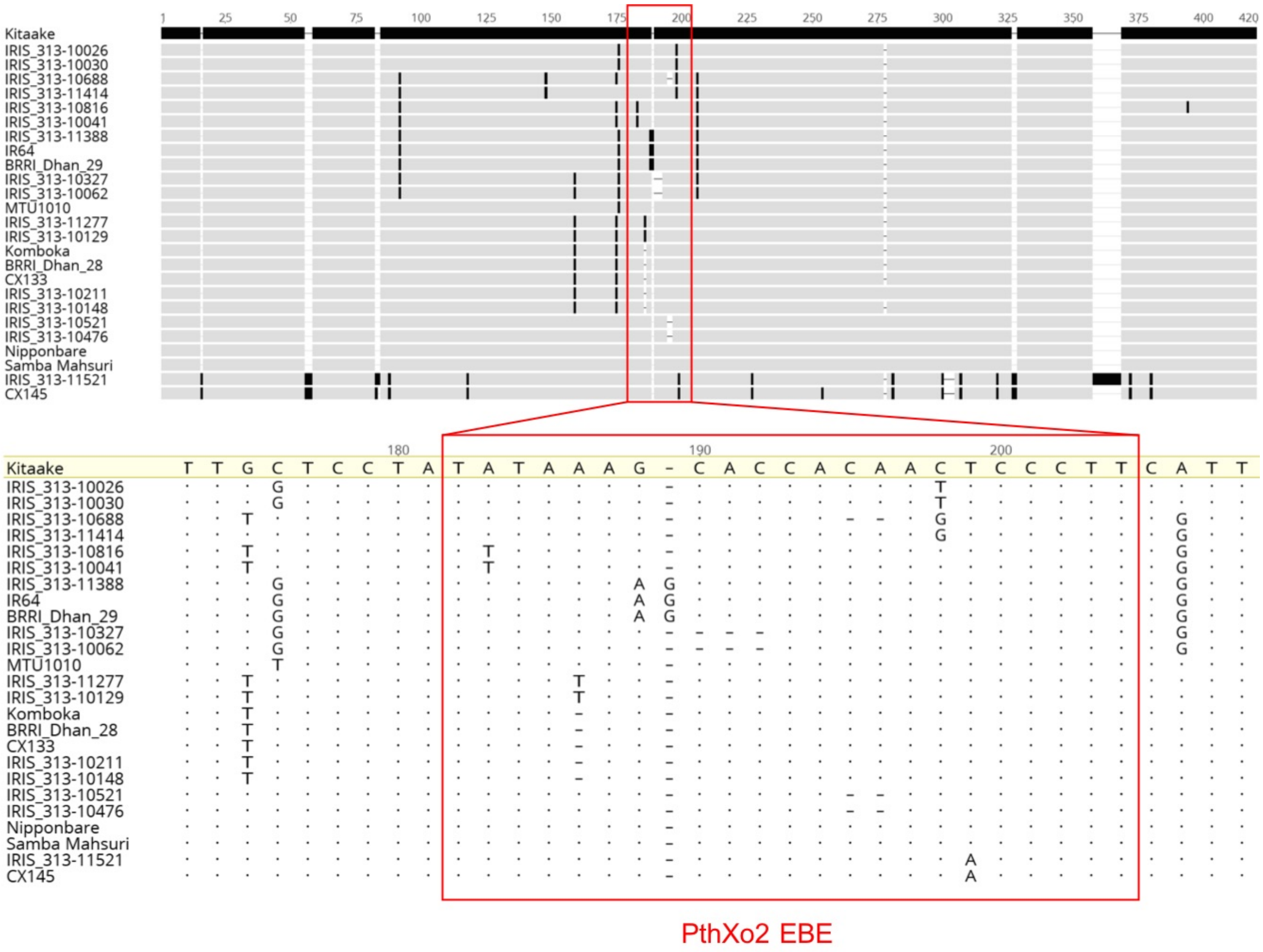

Supplementary Figure 2

Alignment of the *SWEET13* promoter sequences from selected rice varieties.

Rice varieties having nucleotide variations in the PthXo2 EBE were identified using RiceVarMap v.2 (http://ricevarmap.ncpgr.cn/v2/). Two varieties were selected for each variation types as representative. Sequences of the first 400 bp of *SWEET13* promoters of the selected varieties were extracted from the 3K database (http://snp-seek.irri.org/). Alignment was done using ClustalW in Geneious 11.1.5 (https://www.geneious.com). Nine variations were found in the PthXo2 EBE with frequencies ranging from 1.3% to 20.8%.

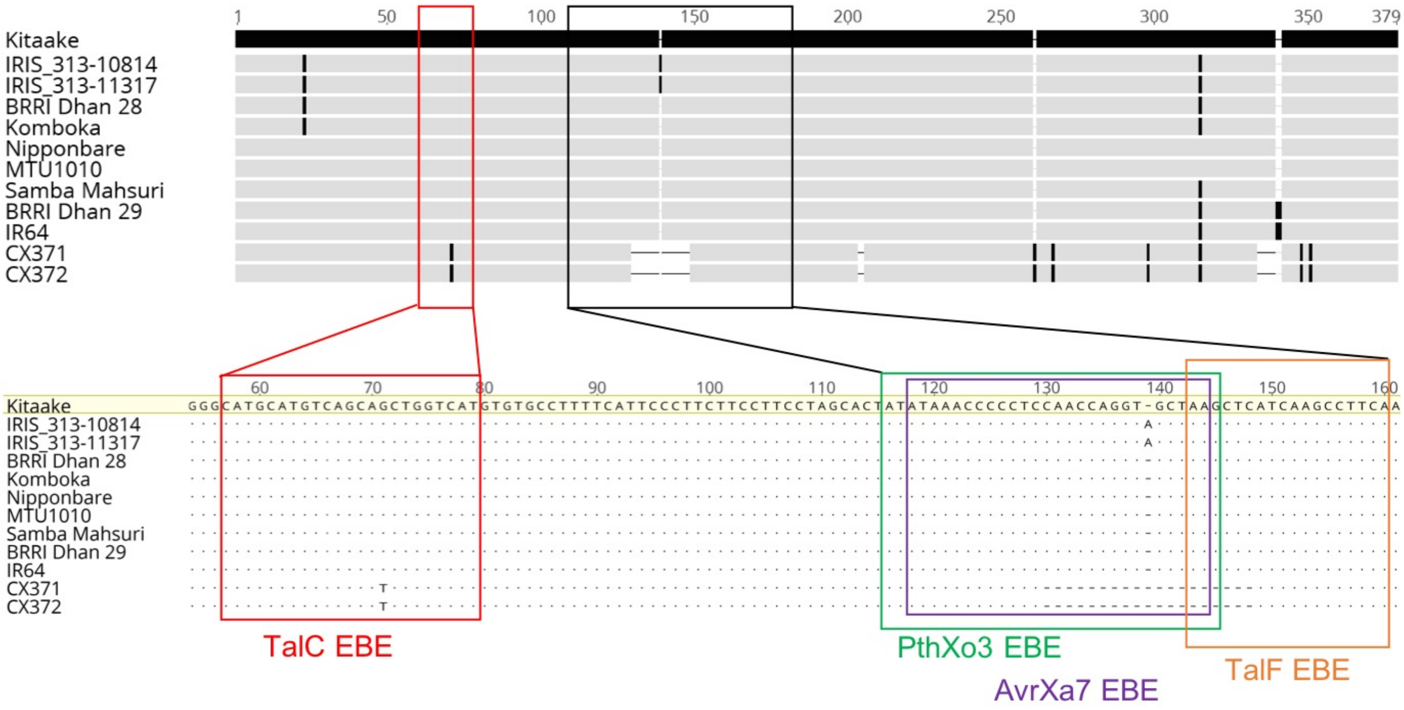

Supplementary Figure 3

Alignment of the *SWEET14* promoter sequences from selected rice varieties.

Rice varieties having nucleotide variations in the PthXo3, TalC, AvrXa7, TalF EBEs were identified using RiceVarMap v.2 (http://ricevarmap.ncpgr.cn/v2/). Two varieties were selected for each variation types as representative. Sequences of the first 400 bp of *SWEET14* promoters of the selected varieties were extracted from the 3K database (http://snp-seek.irri.org/). Alignment was done using ClustalW in Geneious 11.1.5 (https://www.geneious.com). In the PthXo3/AvrXa7 EBEs, there is one A insertion with a frequency of 7.7%. CX371 and CX372 have one G/T variation in the TalC EBE and a 18bp-deletion in the PthXo3/AvrXa7 and TalF EBEs.

a

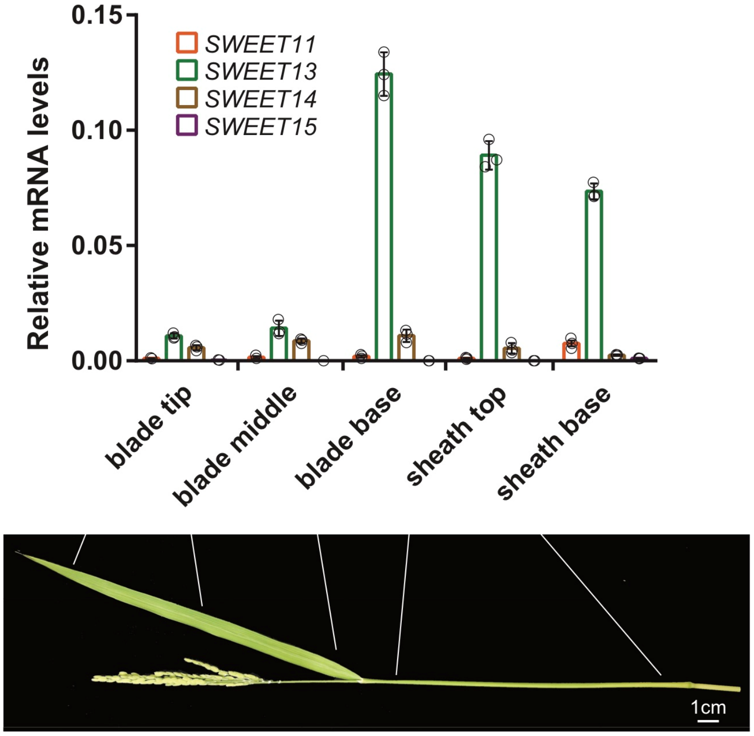

b

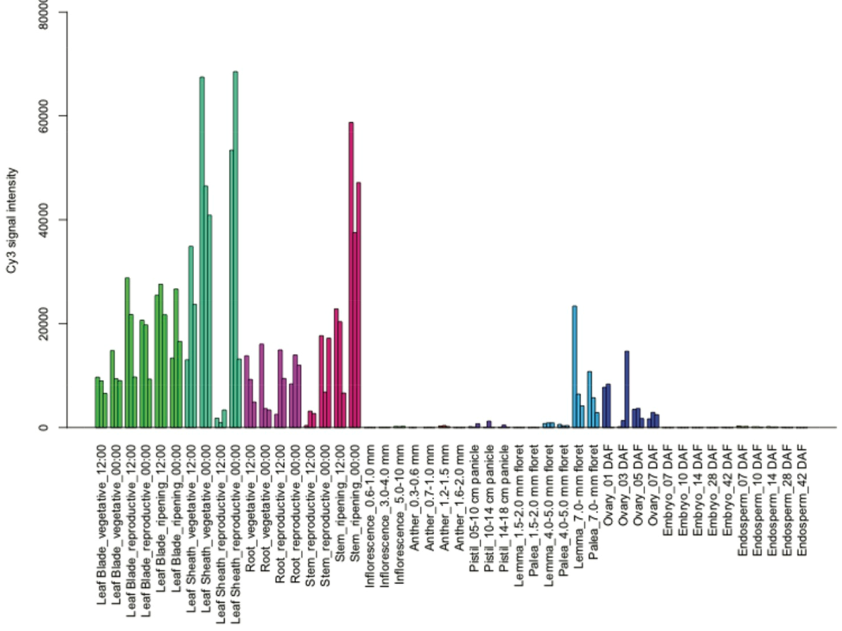

Supplementary Figure 4

***SWEET* mRNA levels in uninfected rice leaves.**

(a) Relative mRNA levels (quantitative RT-PCR) of *SWEET11*, *SWEET13*, *SWEET14* and *SWEET15* in different regions of rice flag leaves. Samples were harvested at noon (mean ± s.e.m., n=3 leaf samples from siblings grown in parallel) with expression normalized to rice *Ubiquitin1* levels; repeated independently three times with comparable results). **(b)** Tissue specific expression pattern of *SWEET13* from public microarray data (http://Ricexpro.dna.affrc.go.jp).

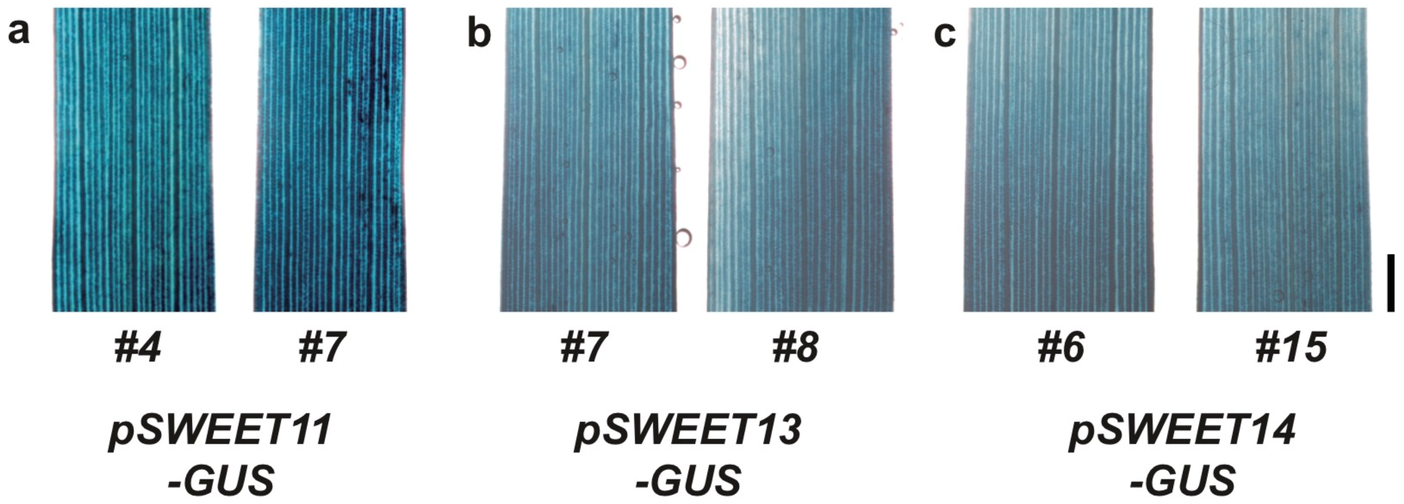

Supplementary Figure 5

Transcriptional fusion reporter lines for SWEET11, 13 and 14.

GUS staining patterns of SWEET11, 13 and 14 transcriptional GUS fusion lines. Transcriptional GUS fusion lines show non-specific expression pattern in leaf tissues.

(a) SWEET11 transcriptional GUS fusion lines. **(b)** SWEET13 transcriptional GUS fusion lines. **(c)** SWEET14 transcriptional GUS fusion lines. Scale Bar: 1mm. This experiment was repeated independently at least three times(n=3 leaf samples from siblings grown in parallel) with comparable results.

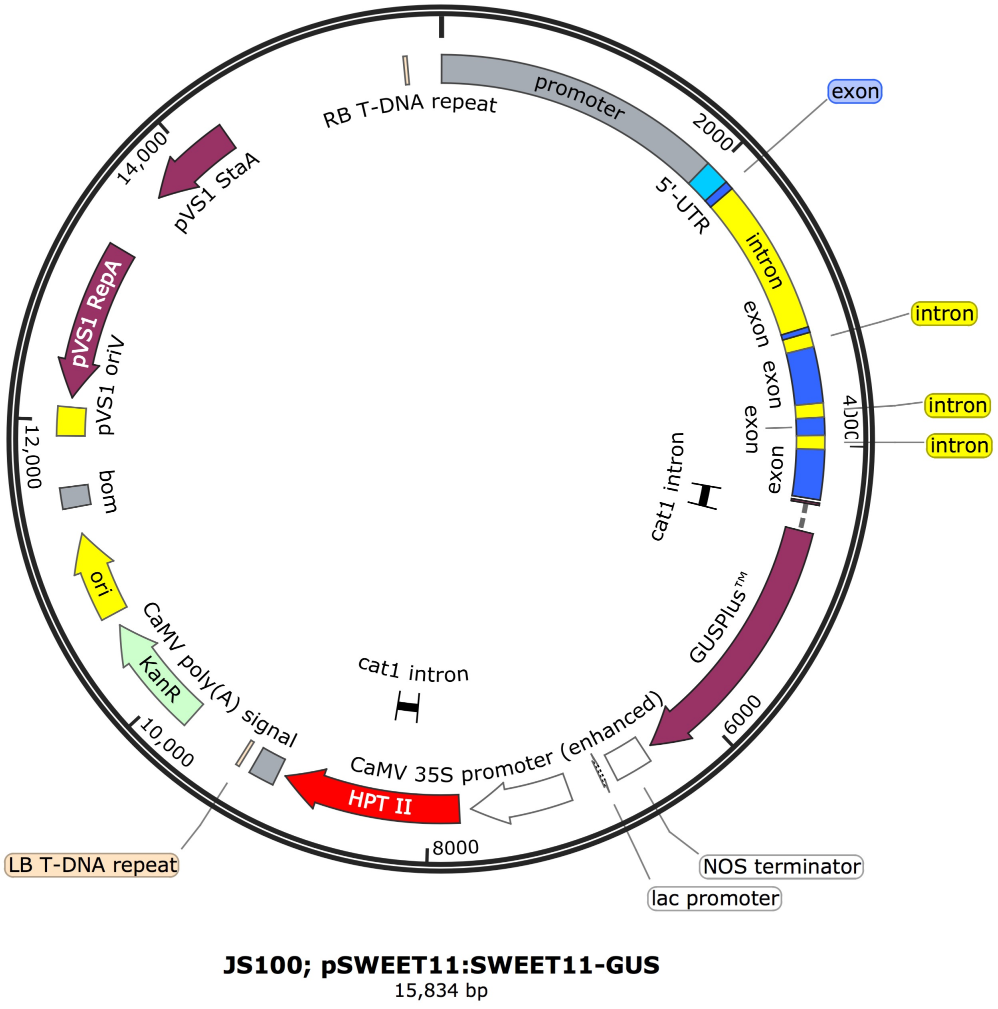

Supplementary Figure 6

Map of the SWEET11 translational reporter fusion constructs

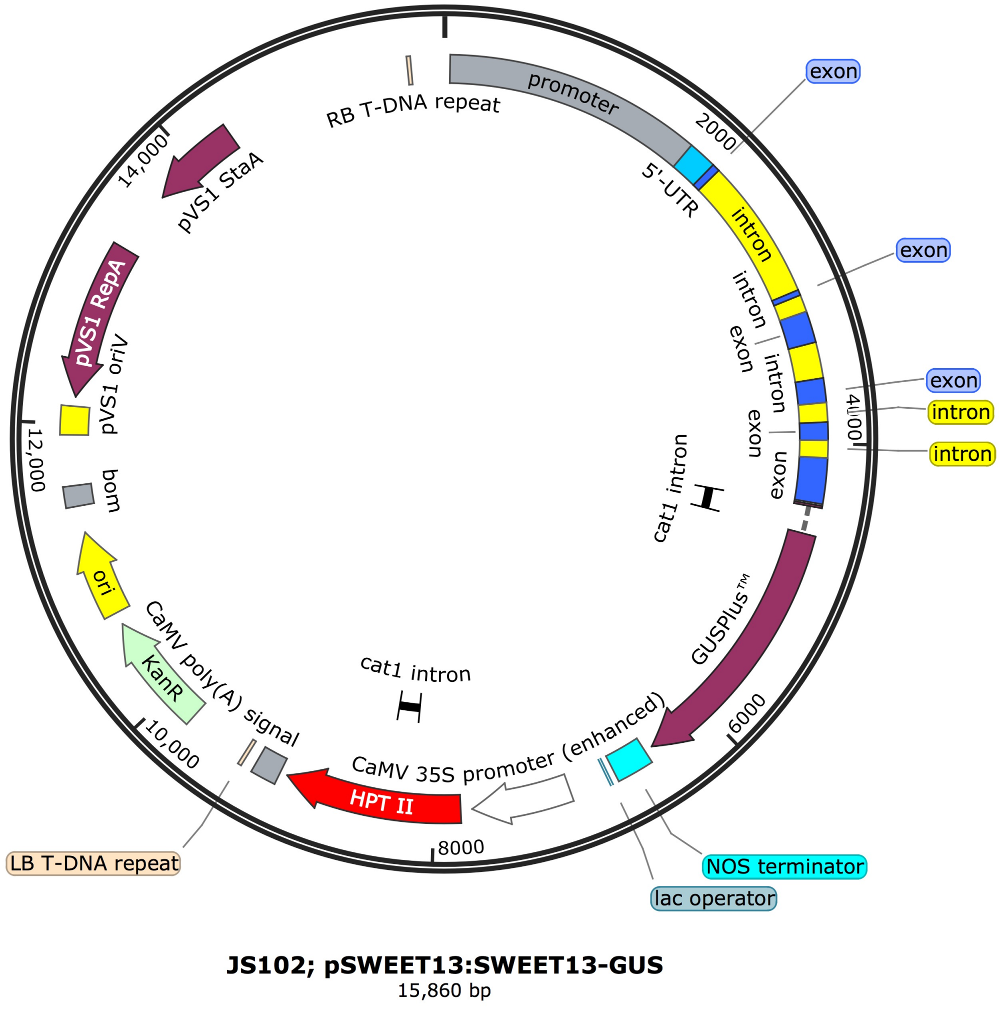

Supplementary Figure 7

Map of the SWEET13 translational reporter fusion constructs

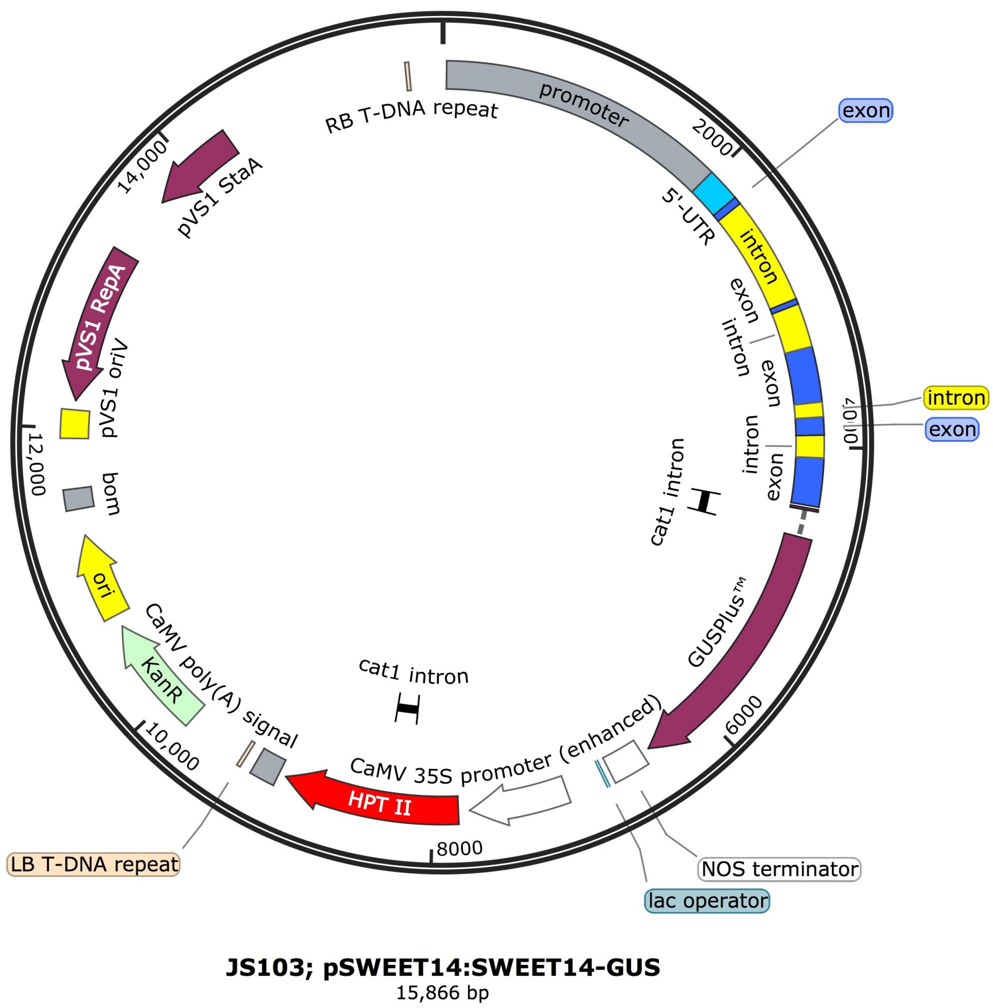

Supplementary Figure 8

Map of the SWEET14 translational reporter fusion constructs

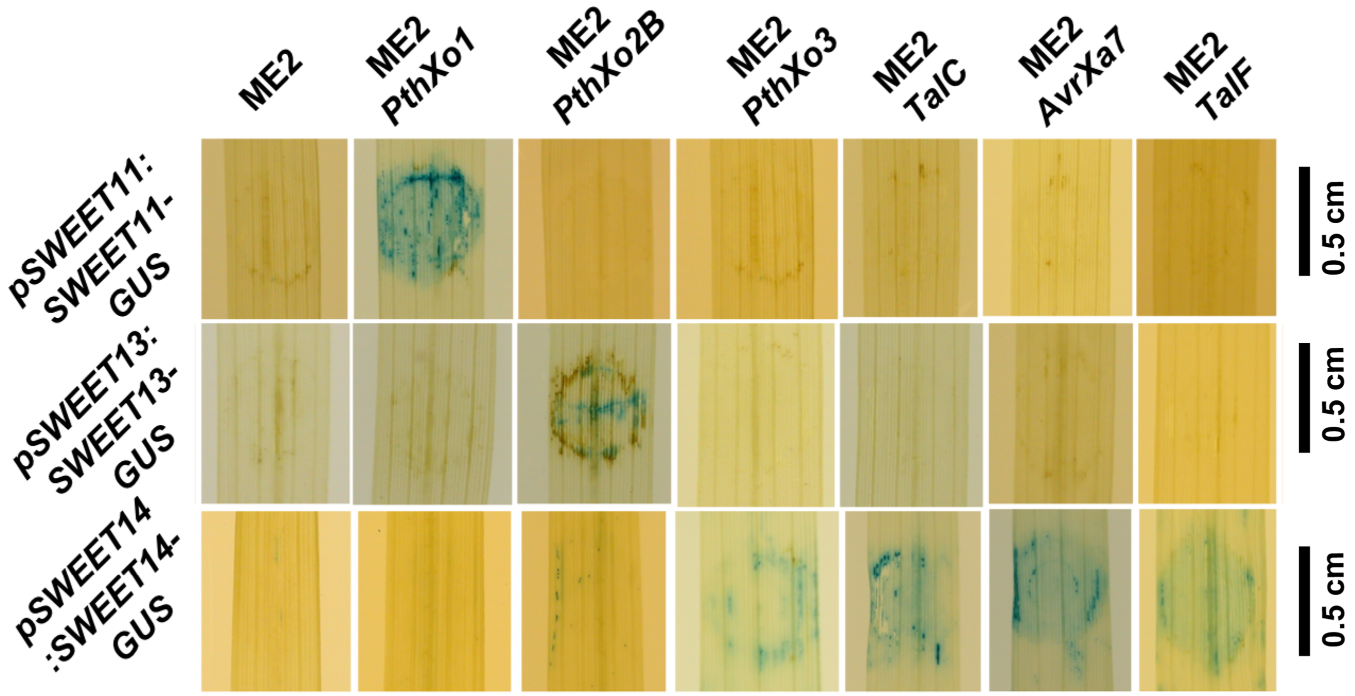

Supplementary Figure 9

SWEET protein accumulation in rice leaves infected with *Xoo* strains expressing a specific TALe.

SWEET protein accumulation upon infection with *Xoo* containing specific TAL effectors. Translational GUS fusion lines were infected with ME2 strain harboring a specific effector. SWEET11 was induced upon inoculation with ME2 expressing the PthXo1 effector. SWEET13 was induced by ME2 with PthXo2B effector. SWEET14 was induced by ME2 with PthXo3, AvrXa7, TalC or TalF. This experiment was repeated independently twice with similar results.

a

SWEET13
ATGGCTGGCCTGTCCCTGCAGCATCCCTGGGCTTTTGCCTTCGGCCTCCTTGgt
M A G L S L Q H P W A F A F G L L

sweet13-1
10nt deletion
ATGGCTGGCCTGTCCCTGCA----------GCTTTTGCCTTCGGCCTCCTTGgt
M A G L S L Q L L P S A S L

sweet13-2
4nt deletion
ATGGCTGGCCTGTCCCTGC----TCCCTGGGCTTTTGCCTTCGGCCTCCTTGgt
M A G L S L L P G L L P S A S L

SWEET14
ATGGCTGGCATGTCTCTTCAGCAT CCCTGGGCCTTCGCCTTTGGTCTCCTAGgt
M A G M S L Q H P W A F A F G L L

sweet14-1
1nt deletion
ATGGCTGGCATGTCTCTTCAGC-T CCCTGGGCCTTCGCCTTTGGTCTCCTAGgt
M A G M S L Q L P G P S P L V S *

sweet14-2
1nt insertion
ATGGCTGGCATGTCTCTTCAGCATTCCCTGGGCCTTCGCCTTTGGTCTCCTAGgt
M A G M S L Q H S L G L R L W S P R

b

sweet13-1	MAGLSLQLLPSASLATSYPSRPIWHQSRRSTGSTRASRRRGSSRCRTWWRSSAPCCGSSTR*
sweet13-2	MAGLSLLPGLLPSASLATSYPSRPIWHQSRRSTGSTRASRRRGSSRCRTWWRSSAPCCGSSTR*
sweet14-1	MAGMSLQLPGPSPLVS*
sweet14-2	MAGMSLQHSLGLRLWSPRQHHLLHDLPGPTADVLQDLQEQVDAGVPVGTLRGGAVQRDAVDLLRAAQVRRVPPHHHQLRWLRHRDHLHRRLPRLRPQEGQDVHRQAPPPRQRRRLRPHPPPHPPPLRRRPPHRGSWLGLRWLLRQRLRRPP*

c

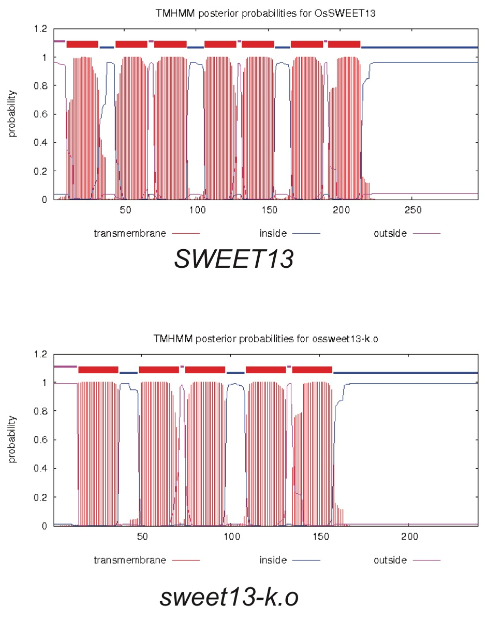

d

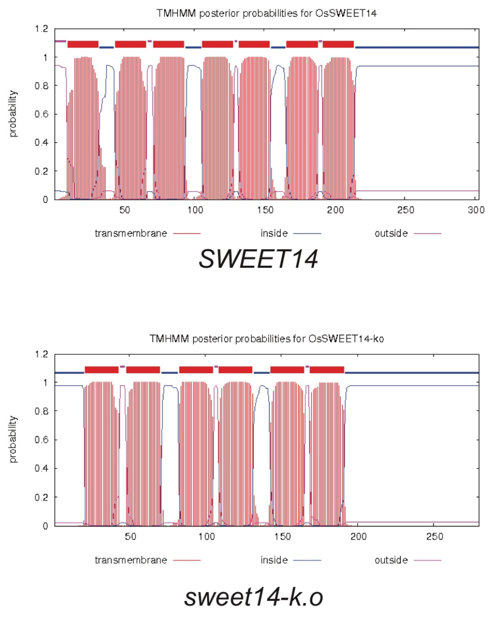

Supplementary Figure 10

CRISPR-Cas9 editing of *SWEET13* and *SWEET14* for *knockout* lines and predicted truncated form of transporters.
Mutagenesis of *SWEET13* and *SWEET14* using CRISPR/Cas9 genome editing. The guide RNA-targeting site is marked with an underline and the protospacer adjacent motif (PAM) is marked in green. **(a)** Mutagenesis scheme of *SWEET13* and *SWEET14*. Dashed line (-) denotes a deleted nucleotide in *sweet13-1* (10 nt), *sweet13-2* (4 nt) and *sweet14-1* (1 nt), respectively. 1nt insertion in *sweet14-2* was marked in blue. Both deletion and frame shift of amino acids occurred in the 1st exon and causes early termination. **(b)** Predicted amino acid sequence of *sweet13-1, sweet13-2*, *sweet14-1* and *sweet14-2*, respectively. In *sweet13-1* and *sweet13-2*, frameshifts occur at the position of codons 8 and 7 of the original open reading frame, respectively, leading to polypeptides with altered sequence and length due to premature stop codons. **(c)** If we assume that the second ATG (codon 58 in wild-type *SWEET13*) were used for protein production, only truncated proteins could be formed. In both mutants, the mutations will lead to loss of the first two transmembrane spanning domains, most likely leading to non-functional transporters. **(d)** Predicted topology of the truncated *SWEET14* protein in the *sweet14-1* and *sweet14-2* mutants in case codon 23 would serve a start codon. In both mutants, the mutations will lead to loss of the first transmembrane spanning domain, most likely leading to non-functional transporters. Typically, premature stop codons affect RNA stability. Moreover, typically only the first ATG is used, thus is likely that all four lines have completely lost the transport functions for the respective *SWEETs*.

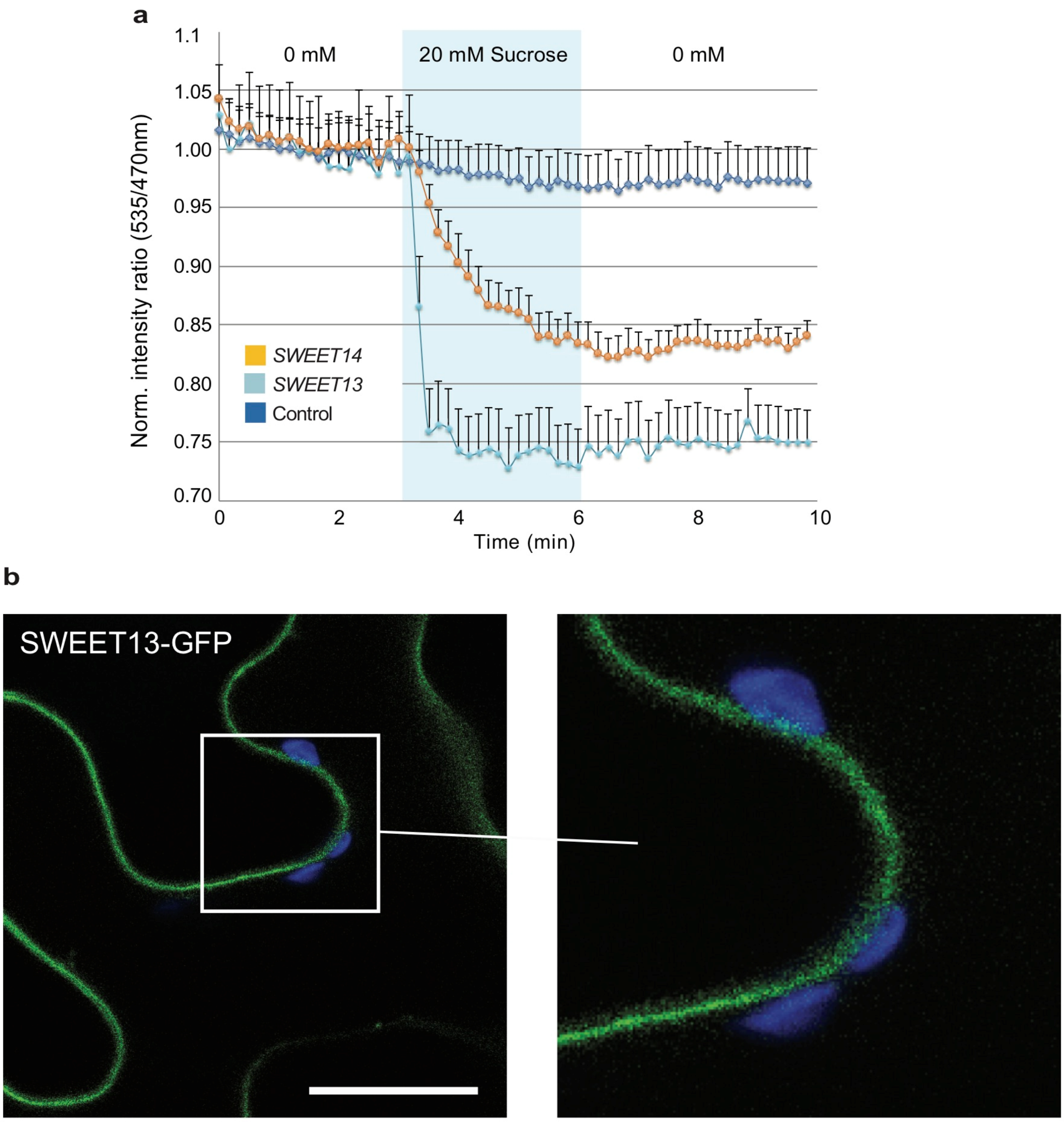

Supplementary Figure 11

Sucrose transport activity and subcellular localization of SWEET13 from rice.

(a) Sucrose transport activity by SWEET13 in HEK293T cells co-expressing the FLIPsuc90µΔ1V sucrose sensor. Cells expressing the sensor without SWEET13 were used as negative controls. SWEET14 served as positive control (mean ± s.e.m.). **(b)** Confocal Z-stack of *Agrobacterium*-infiltrated *N. benthamiana* epidermal leaf cells. ZmSWEET13a-eGFP fluorescence indicated localization at the plasma membrane. The eGFP signal (GREEN) was merged with fluorescence derived from chloroplasts (667– 773 nm) (BLUE). Both experiments were repeated at least three times independently. Scale bar: 50 µm.

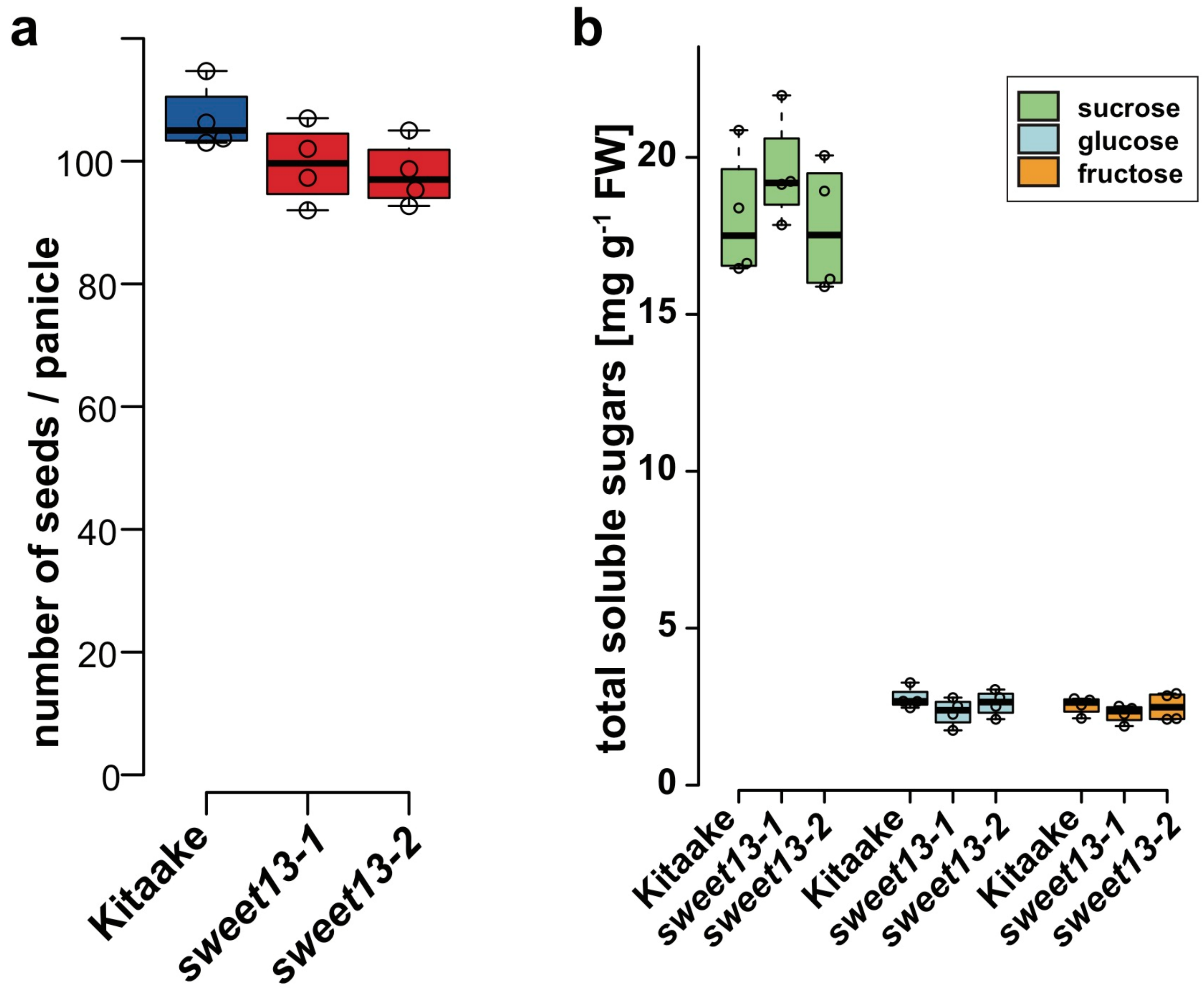

Supplementary Figure 12

Molecular and phenotypic characterization of alleles of *sweet13* mutants.
(a) Number of seeds per panicle of greenhouse-grown wild-type, *sweet13-1* and *-2* lines grown side-by-side (n=4). No significant differences (p-value=0.131 for *sweet13-1* and 0.054 for *sweet13*-2) were observed with Student's t-test. **(b)** Total soluble sugars in wild-type and *sweet13-1* and *-2* flag leaves. Both mutants showed similar sugar concentrations compared to wild-type. Samples were harvested at dusk (8:00 pm; n=4 leaf samples from siblings grown in parallel, and repeated independently at least three times with comparable results). Data were plotted using BoxPlotR (http://shiny.chemgrid.org/boxplotr/). Center lines show medians; box limits indicate 25th and 75th percentiles as determined by R software; data points are plotted as open circles. No significant differences were observed with Student's t-test (*sweet13-1*: p-value=0.318 for sucrose, 0.170 for glucose, 0.242 for fructose, *sweet13-2*: p-value=0.824 for sucrose, 0.573 for glucose, 0.882 for fructose).

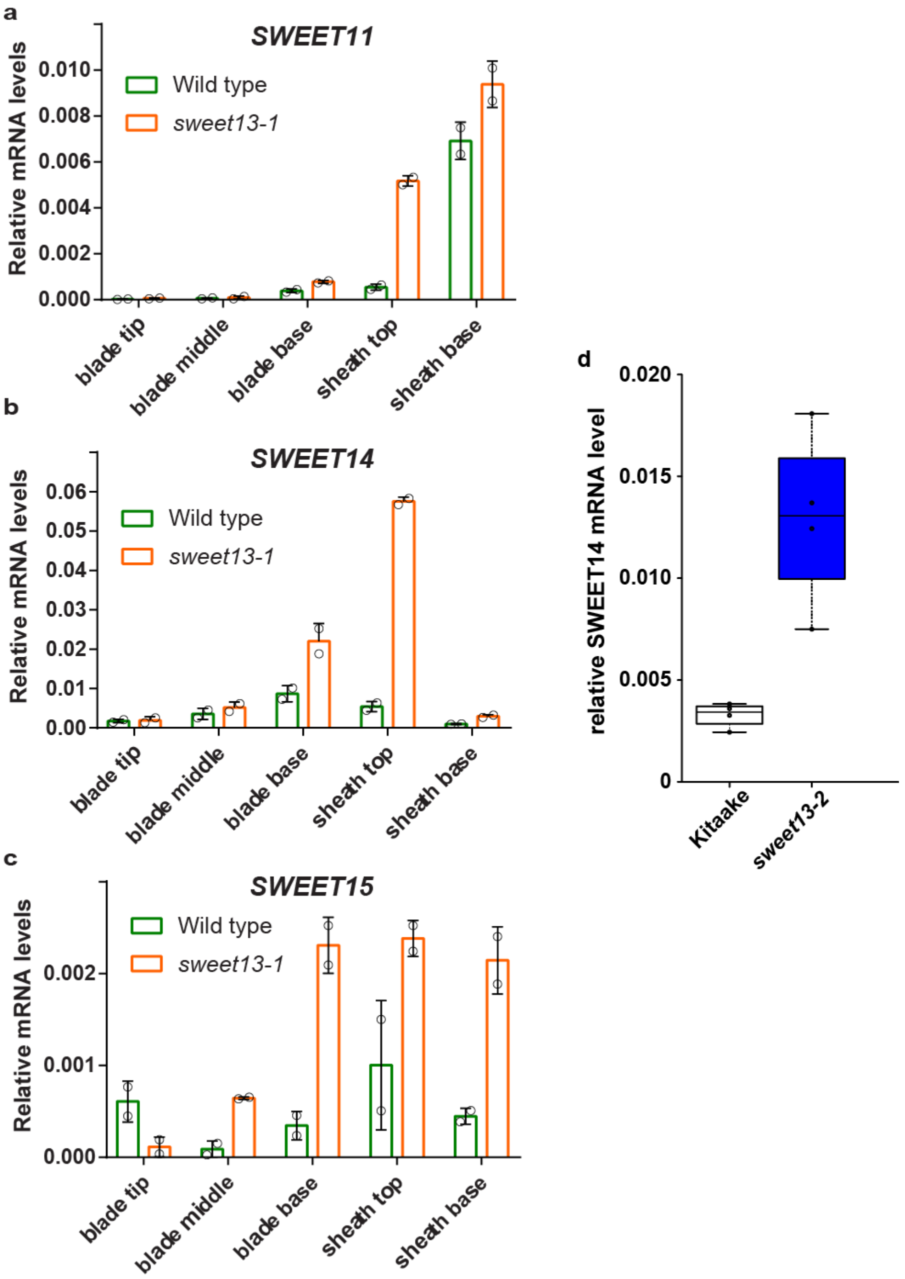

Supplementary Figure 13

mRNA levels of clade III *SWEETs* in *sweet13* mutants.

(a-c) Relative mRNA levels ($2^{-\Delta\Delta Ct}$) of *SWEET11*, *SWEET14* and *SWEET15* in the *sweet13-1* mutant (wild type control: Kitaake). *SWEET14* is the only *SWEET* Clade III that shows significant up-regulation in the mutant (mean ± s.e.m., n=2 leaf samples from siblings grown in parallel, with mRNA levels normalized to the rice *Ubiquitin1* levels, and repeated independently three times with comparable results). (d) *SWEET14* mRNA levels in the region around the laminar joint of the flag leaf (~1 cm of flag leaf blade base region plus laminar joint plus ~1cm of flag leaf sheath top region) of the second allele *sweet13-2* (in blue, wild type control in white: Kitaake; center lines show the medians; box limits indicate the 25th and 75th percentiles as determined by R software; whiskers extend 1.5 times the interquartile range from the 25th and 75th percentiles, outliers are represented by dots; data points are plotted as open circles; n=4 sample points).

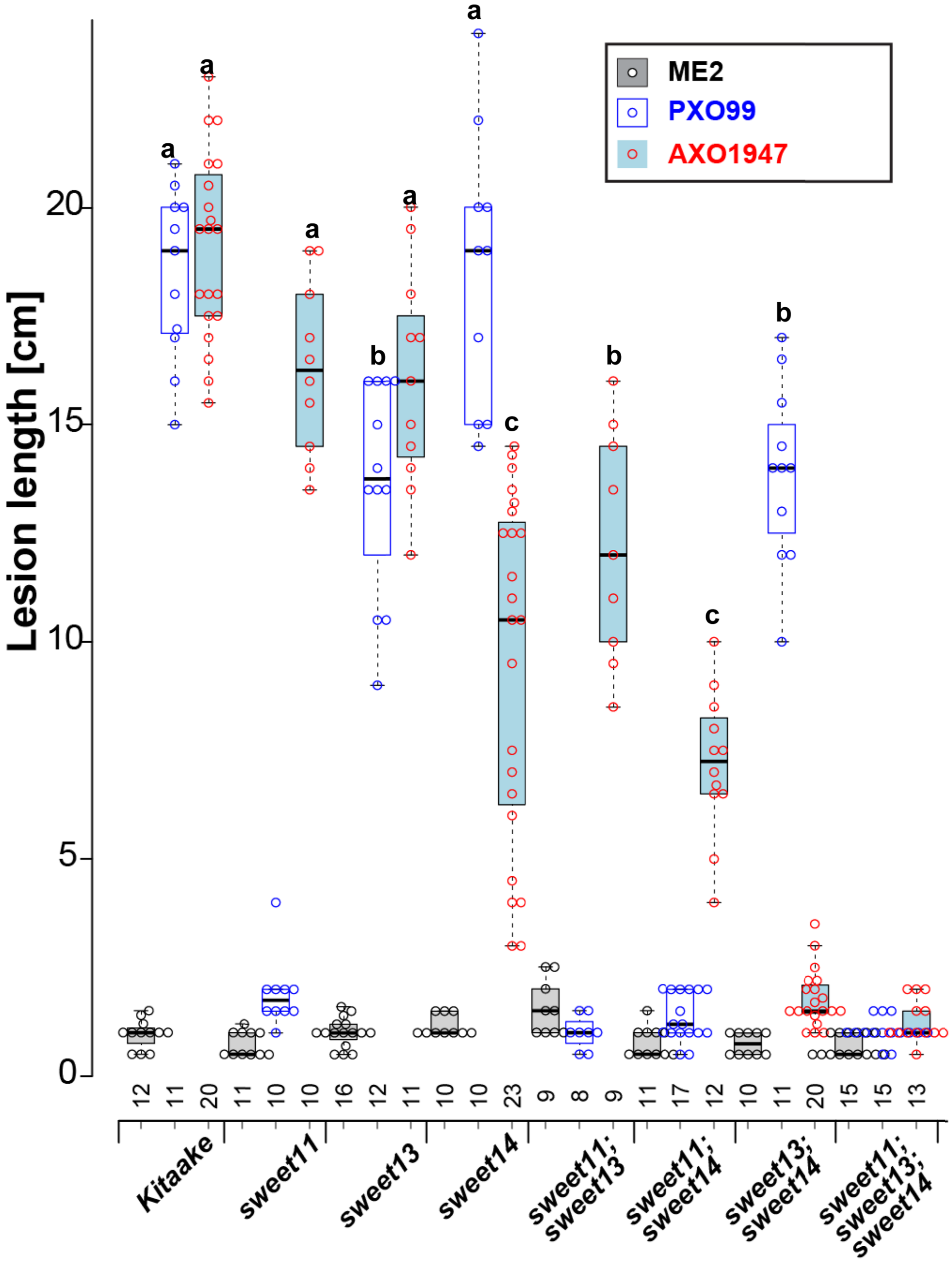

Supplementary Figure 14

SWEET^{ko} *knock out* mutants as diagnostic tools.

Lesion length caused by ME2 (negative control, black circles, grey box outline), PXO99 (positive control, blue circles, blue box outline) and the African strain AXO1947 (red circles, faint blue boxes) on single, double and triple (*sweet11, sweet13* and *sweet14) knock out* mutants relative to Kitaake wild-type. Lesion lengths measured at 14 DAI were plotted using BoxPlotR (http://shiny.chemgrid.org/boxplotr/). Center lines show medians; box limits indicate 25th and 75th percentiles as determined by R software; whiskers extend 1.5 times the interquartile range from 25th and 75th percentiles; data points are plotted as open circles. Number of tests n is indicated below each box on x-axis (numbers between 8 and 23). Same lower letters above the graph bars indicate no significant different at p<0.05. one-way ANOVA.

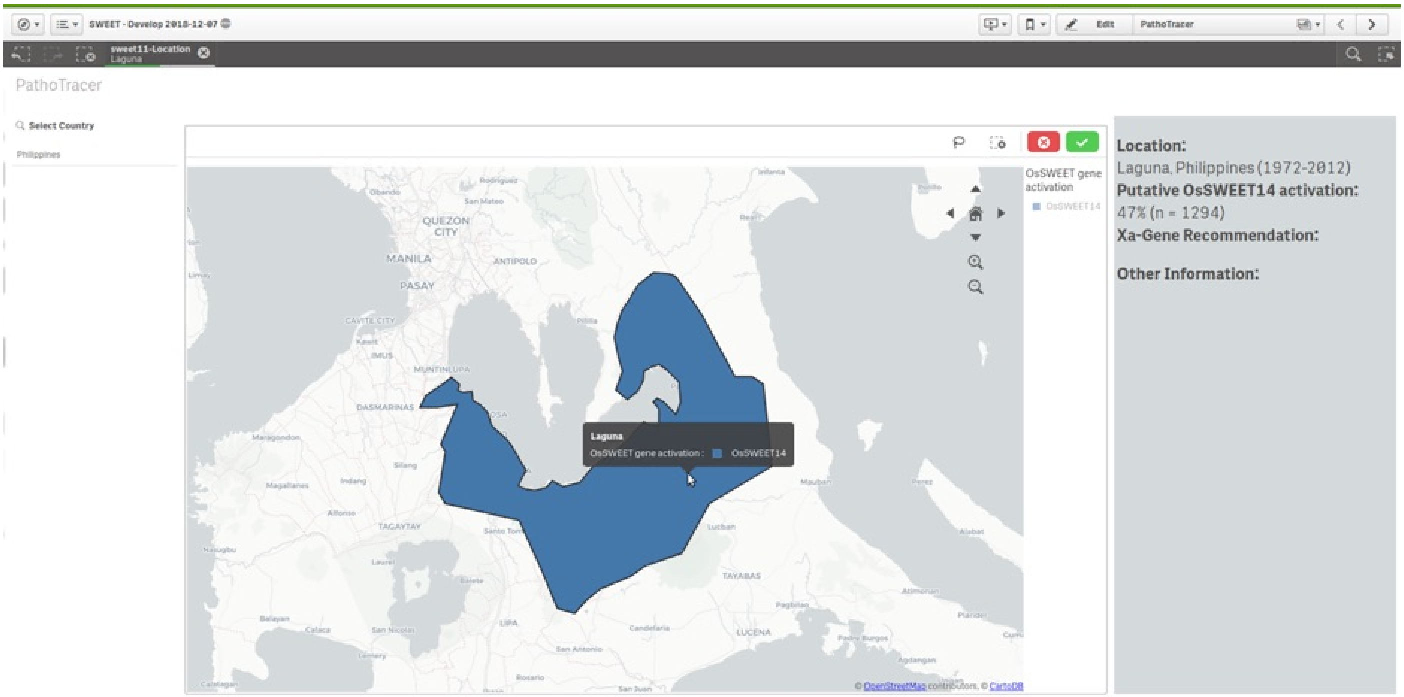

Supplementary Figure 15

PathoTracer visualization under the SWEET^{R} kit 1.0 showing prevalence of *Xoo* strains with putative *SWEET14* induction in the Philippines.

PathoTracer (http://webapps.irri.org/pathotracer/site2/) is an online repository that integrates genotypic and phenotypic pathogen data with resistance profiles of rice accessions to support strategic deployment of varieties in the region. Highlighted in this figure is the population of *Xoo* collected from 1972 – 2012 in Laguna, a BB-disease endemic area in the Philippines (n = 1294 isolates). A screenshot of the same map is shown in Figure 6. The right-hand side panel would display detailed information on the population structure of *Xoo* in that region in relation to putative induction of *SWEET14* (n = 1294 isolates) and any recommended varieties.

Supplementary Tables and Notes

Diagnostic kit for rice blight resistance

Joon-Seob Eom[1,*], Dangping Luo[2,*], Genelou Atienza-Grande[3,4], Jungil Yang[1], Chonghui Ji[2], Van Thi Luu[1], José C. Huguet-Tapia[5], Si Nian Char[6], Bo Liu[2], Hanna Nguyen[3], Sarah Maria Schmidt[1], Boris Szurek[7], Casiana Vera Cruz[3], Frank F. White[5], Ricardo Oliva[3], Bing Yang[2,8,Ψ], Wolf B. Frommer[1,9, Ψ]

11 Supplementary Tables
3 Supplementary Notes

Supplementary Table 1. Contents of the SWEET^R kit 1.0

Supplementary Table 2. Summary of EBEs sequence variations

Supplementary Table 3. Rice lines/varieties used for promoter sequence alignment

Supplementary Table 4. Upstream sequences (400 bp) of SWEET promoters for selected lines/varieties

Supplementary Table 5. List of PCR primers used and their applications.

Supplementary Table 6. DNA Sanger sequencing results for *SWEET* gene knockouts

Supplementary Table 7. Lesion length for individual, double and triple SWEET^{ko} lines (Kitaake)

Supplementary Table 8. Total number of genome-edited EBE variants in three SWEET genes

Supplementary Table 9. Resistance of Kitaake promoter edited lines

Supplementary Table 10. Virulence of Xoo strains on promoter variants of mega varieties

Supplementary Table 11. DNA sequencing results for construct validation

Supplementary Note 1. Standard Operating Procedure HC0002 Screening Genome-edited rice lines for absence of TDNA

Supplementary Note 2. Material Transfer Agreement for accessibility of kit components

Supplementary Note 3. Standard Material Transfer Agreement with Additional Terms and Conditions for Transgenic or Biotechnology Engineered (BE) Materials for biological resources of kit components according to the International Treaty on Plant Genetic Resources for Food and Agriculture.

Supplementary Table 1. Contents of the SWEET^{R} kit 1.0

Type of content	Name	Purpose
SWEET promoter database	SWEETpDB	Identify TALe targets in different rice lines
RT-PCR primer set for SWEET mRNA	SWEET^{up}	Detection of SWEET gene induction by Xoo strain
GUS tester lines (Kitaake)	SWEET^{acc}	Detection of SWEET protein accumulation by Xoo strain
Knock-out tester lines (Kitaake)	SWEET^{ko}	Detection of specific SWEET gene dependence
SWEETp^{R}-tester lines (Kitaake) promoter mutants	SWEETp^{R}	Test whether EBE mutations cause Xoo resistance
Web-based SWEET PathoTracer	SWEET PathoTracer	GIS-based prediction of best R gene deployment
Breeding lines (IR64 & Ciherang-Sub1)	SWEET^{R}	Elite rice lines containing multiple SWEET promoter variants

Supplementary Table 2. Summary of EBE sequence variations

EBE	Var ID	Chromosome	Position	* Reference allele	Primary Allele	Secondary Allele	% Primary Allele in 4726 varieties	% Secondary allele in 4726 varieties	% N **	% Deletions ***	% other alleles
PthXo1	vg0826728868	08	26728868	A	A	G	99.7	0.2	0.08	0.0	NA
PthXo2	vg1217305343	12	17305343	A	A	T	89.1	5.9	0.63	4.3	NA
PthXo2	vg1217305344	12	17305344	G	G	A	85.1	4.9	5.73	4.0	C: 0.23
PthXo2	vg1217305345	12	17305345	TTG	TTG	T	66.7	3.8	2.69	26.8	NA
PthXo2	vg1217305346	12	17305346	TGTG	TGTG	T	70.0	1.9	4.85	23.2	NA
PthXo2	vg1217305353	12	17305353	CT	CT	C	69.4	20.8	0.21	0.0	CTT: 9.59
PthXo2	vg1217305355	12	17305355	T	T	A	78.3	1.3	0.00	20.4	NA
PthXo2	vg1217305358	12	17305358	T	T	A	95.4	4.6	0.02	0.0	NA
PthXo3/AvrXa7	vg1118174486	11	18174486	G	G	A	92.3	7.7	0.02	0.0	NA

* reference allele from Nipponbare

** N is any nucleotide, which is still missing genotype after genome imputation in 3k genome sequence (snp-seek.irri.org/)

*** missing genotype in high coverage region in 3k genome sequence was defined as DEL in imputation (snp-seek.irri.org/)

Supplementary Table 3. Rice lines/varieties used for promoter sequence alignment

Name	IRIS ID	Accession	Sub-population	Country
ZHENGSHAN97	CX133		ind1A	China
MINGHUI 63	CX145	IRGC 117271	indx	China
NERICA 1	CX371	IRGC 136000	trop	Ivory Coast
NERICA 2	CX372	IRGC 136001	trop	Ivory Coast
KITRANA 1007::IRGC 68517-1	IRIS 313-10026	IRGC 125804	ind2	Madagascar
NS 252::IRGC 68878-1	IRIS 313-10030	IRGC 121075	ind2	Madagascar
BOTOHAVANA MENA::IRGC 69349-1	IRIS 313-10041	IRGC 126049	trop	Madagascar
REA::IRGC 73031-1	IRIS 313-10062	IRGC 126141	trop	Greece
JAERAERYUKDO::IRGC 73053-1	IRIS 313-10065	IRGC 127055	trop	South Korea
JEONBUKGUNGWEONNA::IRGC 73055-1	IRIS 313-10067	IRGC 125783	trop	South Korea
SHINA MOCHI::IRGC 74523-1	IRIS 313-10076	IRGC 125900	trop	Japan
JOKJEBICHAL::IRGC 77663-1	IRIS 313-10096	IRGC 125787	japx	South Korea
SAN SHIH TSI::IRGC 1038-1	IRIS 313-10129	IRGC 125897	ind1A	China
CAUVERY::IRGC 45255-1	IRIS 313-10148	IRGC 125695	indx	India
BAI HE::IRGC 76438-1	IRIS 313-10211	IRGC 125669	ind1A	China
CAROLINO BLANCO::IRGC 117249-1	IRIS 313-10327	IRGC 127042	trop	Peru
PACHCHAIPERUMAL 2462-11::IRGC 3474-1	IRIS 313-10476	IRGC 127698	ind2	Sri Lanka
SANNABATHA::IRGC 5971-1	IRIS 313-10521		aro	India
PADI TIMBUN::IRGC 13451-1	IRIS 313-10688	IRGC 135782	ind3	Malaysia
SIBORAS::IRGC 18816-2	IRIS 313-10814	IRGC 128163	ind3	Indonesia
SIDJERO GUNDIL::IRGC 18829-2	IRIS 313-10816	IRGC 128164	trop	Indonesia
ARC 15129::IRGC 41938-1	IRIS 313-11277	IRGC 127157	aus	India
SI RANTING::IRGC 43645-2	IRIS 313-11317	IRGC 128167	ind3	Indonesia
GLAM::IRGC 47978-2	IRIS 313-11388	IRGC 132250	ind3	Thailand
KARUNJEERAGA SAMBA::IRGC 49774-1	IRIS 313-11414	IRGC 127495	indx	India
TAN NONG::IRGC 56126-1	IRIS 313-11521	IRGC 127847	indx	Vietnam
KOMBOKA	IR05N-221	IRGC 56143		Africa
MTU1010		IRGC 84723	indx	India

SAMBHA MAHSURI	IRIS_313-15900	IRGC 126960	indx	India
BRRI_DHAN_28		IRGC 131872	indx	Bangladesh
BRRI_DHAN_29		IRGC 135635	indx	Bangladesh
IR64	CX230	IRGC 135929	indx	the Philippines
KITAAKE		GSOR 30034	japx	Japan
NIPPONBARE	CX140	IRGC 136196	japx	Japan

Supplementary Table 4. Upstream sequences (400 bp) of SWEET promoters for selected lines/varieties

Putative TATA boxes are highlighted in yellow. PthXo1 EBEs are highlighted in green. PthXo2 EBE is in red font. TalC EBEs are highlighted in red. PthXo3 EBEs are colored in green. AvrXa7 EBEs are underlined. TalF EBEs are highlighted in blue.

Gene	Variety	Source	First 400 bp of promoter sequence
SWEET 11	IRIS_313 -10067	3k-DB	ATGACCACATATTCAGAGTAGTGGAGAGAGGGACAGATC<br>TAGAGGTAGAAAAAGAAAATTCATATAAATGATATATCAG<br>AGTGAAAAAGAAATATCAAGCACAAGAAAAAAAAAAAGCA<br>AAGGTTAGATATGCATCTCCCCCTACTGTACACCACCAAA<br>AGTGGAGGGTCTCCAACTATATAAACACTGAGCCATGGC<br>CAAGGCCAAACCACACATGCAGTTGTAGTAGCACTTAAG<br>CCTTCCTCTCTAGCTAGCATCTCTTGTGTCAGGAAGTTG<br>GAAGGGATTTCTGGCTAGTTTCTAGCTGGTGTCTCCTCT<br>CCTCTTCCTAACCTTCTCACTGATTAACACCTTAGAGTTA<br>GTTAATAACCTTCATCACCAGTAGCA
SWEET 11	IRIS_313 -10065	3k-DB	ATGACCACATATTCAGAGTAGTGGAGAGAGGGACAGATC<br>TAGAGGTAGAAAAAGAAAATTCATATAAATGATATATCAG<br>AGTGAAAAAGAAATATCAAGCACAAGAAAAAAAAAAAGCA<br>AAGGTTAGATATGCATCTCCCCCTACTGTACACCACCAAA<br>AGTGGAGGGTCTCCAACTATATAAACACTGAGCCATGGC<br>CAAGGCCAAACCACACATGCAGTTGTAGTAGCACTTAAG<br>CCTTCCTCTCTAGCTAGCATCTCTTGTGTCAGGAAGTTG<br>GAAGGGATTTCTGGCTAGTTTCTAGCTGGTGTCTCCTCT<br>CCTCTTCCTAACCTTCTCACTGATTAACACCTTAGAGTTA<br>GTTAATAACCTTCATCACCAGTAGCA
SWEET 11	IRIS_313 -10076	3k-DB	ATGACCACATATTCAGAGTAGTGGAGAGAGGGACAGATC<br>TAGAGGTAGAAAAAGAAAATTCATATAAATGATATATCAG<br>AGTGAAAAAGAAATATCAAGCACAAGAAAAAAAAGCAAA<br>GGTTAGATATGCATCCCCCCCTACTGTACACCACCAAAA<br>GTGGAGGGTCTCCAACTATATAAACACTGAGCCATGGCC<br>AAGGCCAAACCACACATGCAGTTGTAGTAGCACTTAAGC<br>CTTCCTCTCTAGCTAGCATCTCTTGTGTCAGGAAGTTGGA<br>AGGGATTTCTGGCTAGTTTCTAGCTGGTGTCTCCTCTCCT<br>CTTCCTAACCTTCTCACTGATTAACACCTTAGAGTTAGTT<br>AATAACCTTCATCACCAGTAGCA
SWEET 11	IRIS_313 -10096	3k-DB	ATGACCACATATTCAGAGTAGTGGAGAGAGGGACAGATC<br>TAGAGGTAGAAAAAGAAAATTCATATAAATGATATATCAG<br>AGTGAAAAAGAAATATCAAGCACAAGAAAAAAAAGCAAA<br>GGTTAGATATGCATCCCCCCCTACTGTACACCACCAAAA<br>GTGGAGGGTCTCCAACTATATAAACACTGAGCCATGGCC<br>AAGGCCAAACCACACATGCAGTTGTAGTAGCACTTAAGC<br>CTTCCTCTCTAGCTAGCATCTCTTGTGTCAGGAAGTTGGA<br>AGGGATTTCTGGCTAGTTTCTAGCTGGTGTCTCCTCTCCT<br>CTTCCTAACCTTCTCACTGATTAACACCTTAGAGTTAGTT<br>AATAACCTTCATCACCAGTAGCA
SWEET 11	Kitaake	this work	ATGACCACATATTCAGAGTAGTGGAGAGAGGGACAGATC<br>TAGAGGTAGAAAAAGAAAATTCATATAAATGATATATCAG

			AGTGAAAAAGAAATATCAAGCACAAGAAAAAAAAGCAAA<br>GGTTAGATATGCATCTCCCCCTACTGTACACCACCAAAA<br>GTGGAGGGTCTCCAACTATATAAACACTGAGCCATGGCC<br>AAGGCCAAACCACACATGCAGTTGTAGTAGCACTTAAGC<br>CTTCCTCTCTAGCTAGCATCTCTTGTGTCAGGAAGTTGGA<br>AGGGATTTCTGGCTAGTTTCTAGCTGGTGTCTCCTCTCCT<br>CTTCCTAACCTTCTCACTGATTAACACCTTAGAGTTAGTT<br>AATAACCTTCATCACCAGTAGCA
SWEET 11	Nipponbare	3k-DB	ATGACCACATATTCAGAGTAGTGGAGAGAGGGACAGATC<br>TAGAGGTAGAAAAAGAAAATTCATATAAATGATATATCAG<br>AGTGAAAAAGAAATATCAAGCACAAGAAAAAAAAGCAAA<br>GGTTAGATATGCATCTCCCCCTACTGTACACCACCAAAA<br>GTGGAGGGTCTCCAACTATATAAACACTGAGCCATGGCC<br>AAGGCCAAACCACACATGCAGTTGTAGTAGCACTTAAGC<br>CTTCCTCTCTAGCTAGCATCTCTTGTGTCAGGAAGTTGGA<br>AGGGATTTCTGGCTAGTTTCTAGCTGGTGTCTCCTCTCCT<br>CTTCCTAACCTTCTCACTGATTAACACCTTAGAGTTAGTT<br>AATAACCTTCATCACCAGTAGCA
SWEET 11	Samba Mahsuri	this work	ATGACCACATATTCAGAGTAGTGGAGAGAGGGACAGATC<br>TAGAGGTAGAAAAAGAAAATTCATATAAATGATATATCAG<br>AGTGAAAAAGAAATATCAAGCACAAGAAAAAAAAAACAAA<br>GGTTAGATATGCATCTCCCCCTACTGTACACCACCAAAA<br>GTGGAGGGTCTCCAACTATATAAACACTGAGCCATGGCC<br>AAGGCCAAACCACACATGCAGTTGTAGTAGCACTTAAGC<br>CTTCCTCTCTAGCTAGCATCTCTTGTGTCAGGAAGTTGGA<br>AGGGATTTCTGGCTAGTTTCTAGCTGGTGTCTCCTCTCCT<br>CTTCCTAACCTTCTCACTGATTAACACCTTAGAGTTAGTT<br>AATAACCTTCATCACTAGTAGCA
SWEET 11	BRRI_Dhan_29	this work	ATGACCACATATTCAGAGTAGTGGAGAGAGGGACAGATC<br>TAGAGGTAGAAAAAGAAAATTCATATAAATGATATATCAG<br>AGTGAAAAAGAAATATCAAGCACAAGAAAAAAAAAACAAA<br>GGTTAGATATGCATCTCCCCCTACTGTACACCACCAAAA<br>GTGGAGGGTCTCCAACTATATAAACACTGAGCCATGGCC<br>AAGGCCAAACCACACATGCAGTTGTAGTAGCACTTAAGC<br>CTTCCTCTCTAGCTAGCATCTCTTGTGTCAGGAAGTTGGA<br>AGGGATTTCTGGCTAGTTTCTAGCTGGTGTCTCCTCTCCT<br>CTTCCTAACCTTCTCACTGATTAACACCTTAGAGTTAGTT<br>AATAACCTTCATCACTAGTAGCA
SWEET 11	Komboka	this work	ATGACCACATATTCAGAGTAGTGGAGAGAGGGACAGATC<br>TAGAGGTAGAAAAAGAAAATTCATATAAATGATATATCAG<br>AGTGAAAAAGAAATATCAAGCACAAGAAAAAAAAAAGCAA<br>AGGTTAGATATGCATCTCCCCCTACTGTACACCACCAAAA<br>GTGGAGGGTCTCCAACTATATAAACACTGAGCCATGGCC<br>AAGGCCAAACCACACATGCAGTTGTAGTAGCACTTAAGC<br>CTTCCTCTCTAGCTAGCATCTCTTGTGTCAGGAAGTTGGA<br>AGGGATTTCTGGCTAGTTTCTAGCTGGTGTCTCCTCTCCT<br>CTTCCTAACCTTCTCACTGATTAACACCTTAGAGTTAGTT<br>AATAACCTTCATCACCAGTAGCA
SWEET 11	BRRI_Dhan_28	this work	ATGACCACATATTCAGAGTAGTGGAGAGAGGGACAGATC<br>TAGAGGTAGAAAAAGAAAATTCATATAAATGATATATCAG<br>AGTGAAAAAGAAATATCAAGCACAAGAAAAAAAAAAGCAA

			AGGTTAGATATGCATCTCCCCCTACTGTACACCACCAAAA<br>GTGGAGGGTCTCCAACTATATAAACACTGAGCCATGGCC<br>AAGGCCAAACCACACATGCAGTTGTAGTAGCACTTAAGC<br>CTTCCTCTCTAGCTAGCATCTCTTGTGTCAGGAAGTTGGA<br>AGGGATTTCTGGCTAGTTTCTAGCTGGTGTCTCCTCTCCT<br>CTTCCTAACCTTCTCACTGATTAACACCTTAGAGTTAGTT<br>AATAACCTTCATCACCAGTAGCA
SWEET 11	MTU1010	this work	ATGACCACATATTCAGAGTAGTGGAGAGAGGGACAGATC<br>TAGAGGTAGAAAAAGAAAATTCATATAAATGATATATCAG<br>AGTGAAAAAGAAATATCAAGCACAAGAAAAAAAAAAGCAA<br>AGGTTAGATATGCATCTCCCCCTACTGTACACCACCAAAA<br>GTGGAGGGTCTCCAACTATATAAACACTGAGCCATGGCC<br>AAGGCCAAACCACACATGCAGTTGTAGTAGCACTTAAGC<br>CTTCCTCTCTAGCTAGCATCTCTTGTGTCAGGAAGTTGGA<br>AGGGATTTCTGGCTAGTTTCTAGCTGGTGTCTCCTCTCCT<br>CTTCCTAACCTTCTCACTGATTAACACCTTAGAGTTAGTT<br>AATAACCTTCATCACCAGTAGCA
SWEET 11	IR64	this work	ATGACCACATATTCAGAGTAGTGGAGAGAGGGACAGATC<br>TAGAGGTAGAAAAAGAAAATTCATATAAATGATATATCAG<br>AGTGAAAAAGAAATATCAAGCACAAGAAAAAAAAAAGCAA<br>AGGTTAGATATGCATCTCCCCCTACTGTACACCACCAAAA<br>GTGGAGGGTCTCCAACTATATAAACACTGAGCCATGGCC<br>AAGGCCAAACCACACATGCAGTTGTAGTAGCACTTAAGC<br>CTTCCTCTCTAGCTAGCATCTCTTGTGTCAGGAAGTTGGA<br>AGGGATTTCTGGCTAGTTTCTAGCTGGTGTCTCCTCTCCT<br>CTTCCTAACCTTCTCACTGATTAACACCTTAGAGTTAGTT<br>AATAACCTTCATCACCAGTAGCA
SWEET 13	IRIS_313-10026	3k-DB	CAGTAGCTCATTTTTGTAAAAGCCTAATTATTGTGCGTGT<br>CCAAAAGACTTTCCTCAAAAGCAAATAAAGAAAAAAAATC<br>TTTGCATAATTATTCTATGATTACTTTGATGCGTACGTGAA<br>TGGCCATGGGTAG<br>GAGGCAACCAAGTGATTCCCACCTAGCTAGCTTTGGTCC<br>TATATAAAGCACCACAATTCCCTTCATTCCTCTCCAAGAG<br>TTTTCAGCCAACACATTGAACTCTTCTTCAGAGCTCTCCC<br>TTCCCTCCACAAAGGGGTCTAGGGTTAGAGTGTGTGTGT<br>CTGTGACAAGTTCCAAGCTAGCAACAACAAGCTCAATTC<br>CTTGCTTGTTTGCTTCCATATTACACTACATCTCTTCCCTT<br>CAATTACCCCCCTTTTAGCACACAAAA
SWEET 13	IRIS_313-10030	3k-DB	CAGTAGCTCATTTTTGTAAAAGCCTAATTATTGTGCGTGT<br>CCAAAAGACTTTCCTCAAAAGCAAATAAAGAAAAAAAATC<br>TTTGCATAATTATTCTATGATTACTTTGATGCGTACGTGAA<br>TGGCCATGGGTAGGAGGCAACCAAGTGATTCCCACCTA<br>GCTAGCTTTGGTCCTATATAAAGCACCACAATTCCCTTCA<br>TTCCTCTCCAAGAGTTTTCAGCCAACACATTGAACTCTTC<br>TTCAGAGCTCTCCCTTCCCTCCACAAAGGGGTCTAGGGT<br>TAGAGTGTGTGTGTCTGTGACAAGTTCCAAGCTAGCAAC<br>AACAAGCTCAATTCCTTGCTTGTTTGCTTCCATATTACAC<br>TACATCTCTTCCCTTCAATTACCCCCCTTTTAGCACACAA<br>AA

SWEET 13	MTU1010	this work	CAGTAGCTCATTTTTGTAAAAGCCTAATTATTGTGCGTGT<br>CCAAAAGACTTTCCTCAAAAGCAAATAAAGAAAAAAAATC<br>TTTGCATAATTATTCTATGATTACTTTGATGCGTACGTGAA<br>TGGCCATGGGTAG<br>GAGGCAACCAAGTGATTCCCACCTAGCTAGCTTTGTTCC<br>TATATAAAGCACCACAACTCCCTTCATTCCTCTCCAAGAG<br>TTTTCAGCCAACACATTGAACTCTTCTTCAGAGCTCTCCC<br>TTCCCTCCACAAA<br>GGGGTCTAGGGTTAGAGTGTGTGTGTCTGTGACAAGTTC<br>CAAGCTAGCAACAACAAGCTCAATTCCTTGCTTGTTTGCT<br>TCCATATTACACTACATCTCTTCCCTTCAATTACCCCCCTT<br>TTAGCACACAAAA
SWEET 13	IRIS_313-10688	3k-DB	CAGTAGCTCATTTTTGTAAAAGCCTAATTATTGTGCGTGT<br>CCAAAAGACTTTCCTCAAAAGCAAATAAAGAAAAAAAATC<br>TTTGCGTAATTATTCTATGATTACTTTGATGCGTACGTGA<br>ATGGCCATGGGTAGGAGGCAATCAAGTGATTCCCACCTA<br>GCTAGCTTTTCTCCTATATAAAGCACCAAGTCCCTTCGTT<br>CCTCTCCAAGAGTTTTCAGCCAACACATTGAACTCTTCTT<br>CAGAGCTCTCCCTTCCCTCCACAAAGGGGTCTAGGGTTA<br>GAGTGTGTGTGTCTGTGACAAGTTCCAAGCTAGCAACAA<br>CAAGCTCAATTCCTTGCTTGTTTGCTTCCATATTACACTA<br>CATCTCTTCCCTTCAATTACCCCCCTTTTAGCACACAAAA
SWEET 13	IRIS_313-11414	3k-DB	CAGTAGCTCATTTTTGTAAAAGCCTAATTATTGTGCGTGT<br>CCAAAAGACTTTCCTCAAAAGCAAATAAAGAAAAAAAATC<br>TTTGCGTAATTATTCTATGATTACTTTGATGCGTACGTGA<br>ATGGCCATGGGTAGGAGGCAATCAAGTGATTCCCACCTA<br>GCTAGCTTTGCTCCTATATAAAGCACCACAAGTCCCTTCG<br>TTCCTCTCCAAGAGTTTTCAGCCAACACATTGAACTCTTC<br>TTCAGAGCTCTCCCTTCCCTCCACAAAGGGGTCTAGGGT<br>TAGAGTGTGTGTGTCTGTGACAAGTTCCAAGCTAGCAAC<br>AACAAGCTCAATTCCTTGCTTGTTTGCTTCCATATTACAC<br>TACATCTCTTCCCTTCAATTACCCCCCTTTTAGCACACAA<br>AA
SWEET 13	IRIS_313-11388	3k-DB	CAGTAGCTCATTTTTGTAAAAGCCTAATTATTGTGCGTGT<br>CCAAAAGACTTTCCTCAAAAGCAAATAAAGAAAAAAAATC<br>TTTGCGTAATTATTCTATGATTACTTTGATGCGTACGTGA<br>ATGGCCATGGGTAGGAGGCAACCAAGTGATTCCCACCTA<br>GCTAGCTTTGGTCCTATATAAAAGCACCACAACTCCCTTC<br>GTTCCTCTCCAAGAGTTTTCAGCCAACACATTGAACTCTT<br>CTTCAGAGCTCTCCCTTCCCTCCACAAAGGGGTCTAGGG<br>TTAGAGTGTGTGTGTCTGTGACAAGTTCCAAGCTAGCAA<br>CAACAAGCTCAATTCCTTGCTTGTTTGCTTCCATATTACA<br>CTACATCTCTTCCCTTCAATTACCCCCCTTTTAGCACACA<br>AAA
SWEET 13	IR64	this work	CAGTAGCTCATTTTTGTAAAAGCCTAATTATTGTGCGTGT<br>CCAAAAGACTTTCCTCAAAAGCAAATAAAGAAAAAAAATC<br>TTTGCGTAATTATTCTATGATTACTTTGATGCGTACGTGA<br>ATGGCCATGGGTAGGAGGCAACCAAGTGATTCCCACCTA<br>GCTAGCTTTGGTCCTATATAAAAGCACCACAACTCCCTTC<br>GTTCCTCTCCAAGAGTTTTCAGCCAACACATTGAACTCTT<br>CTTCAGAGCTCTCCCTTCCCTCCACAAAGGGGTCTAGGG

			TTAGAGTGTGTGTGTCTGTGACAAGTTCCAAGCTAGCAA<br>CAACAAGCTCAATTCCTTGCTTGTTTGCTTCCATATTACA<br>CTACATCTCTTCCCTTCAATTACCCCCCTTTTAGCACACA<br>AAA
SWEET 13	IRIS_313-10327	3k-DB	CAGTAGCTCATTTTTGTAAAAGCCTAATTATTGTGCGTGT<br>CCAAAAGACTTTCCTCAAAAGCAAATAAAGAAAAAAAATC<br>TTTGCGTAATTATTCTATGATTACTTTGATGCGTACGTGA<br>ATGGCCATGGGTAGGAGGCAACCAAGTGATTCGCACCTA<br>GCTAGCTTTGGTCCTATATAAAGCACAACTCCCTTCGTTC<br>CTCTCCAAGAGTTTTCAGCCAACACATTGAACTCTTCTTC<br>AGAGCTCTCCCTTCCCTCCACAAAGGGGTCTAGGGTTAG<br>AGTGTGTGTGTCTGTGACAAGTTCCAAGCTAGCAACAAC<br>AAGCTCAATTCCTTGCTTGTTTGCTTCCATATTACACTAC<br>ATCTCTTCCCTTCAATTACCCCCCTTTTAGCACACAAAA
SWEET 13	IRIS_313-10062	3k-DB	CAGTAGCTCATTTTTGTAAAAGCCTAATTATTGTGCGTGT<br>CCAAAAGACTTTCCTCAAAAGCAAATAAAGAAAAAAAATC<br>TTTGCGTAATTATTCTATGATTACTTTGATGCGTACGTGA<br>ATGGCCATGGGTAGGAGGCAACCAAGTGATTCGCACCTA<br>GCTAGCTTTGGTCCTATATAAAGCACAACTCCCTTCGTTC<br>CTCTCCAAGAGTTTTCAGCCAACACATTGAACTCTTCTTC<br>AGAGCTCTCCCTTCCCTCCACAAAGGGGTCTAGGGTTAG<br>AGTGTGTGTGTCTGTGACAAGTTCCAAGCTAGCAACAAC<br>AAGCTCAATTCCTTGCTTGTTTGCTTCCATATTACACTAC<br>ATCTCTTCCCTTCAATTACCCCCCTTTTAGCACACAAAA
SWEET 13	IRIS_313-10816	3k-DB	CAGTAGCTCATTTTTGTAAAAGCCTAATTATTGTGCGTGT<br>CCAAAAGACTTTCCTCAAAAGCAAATAAAGAAAAAAAATC<br>TTTGCGTAATTATTCTATGATTACTTTGATGCGTACGTGA<br>ATGGCCATGGGTAGGAGGCAACCAAGTGATTCCCACCTA<br>GCTAGCTTTTCTCCTATTTAAAGCACCACAACTCCCTTCG<br>TTCCTCTCCAAGAGTTTTCAGCCAACACATTGAACTCTTC<br>TTCAGAGCTCTCCCTTCCCTCCACAAAGGGGTCTAGGGT<br>TAGAGTGTGTGTGTCTGTGACAAGTTCCAAGCTAGCAAC<br>AACAAGCTCAATTCCTTGCTTGTTTGCTTCCATATTACAC<br>TACATCTCTTCCCTTTAATTACCCCCCTTTTAGCACACAA<br>AA
SWEET 13	IRIS_313-10041	3k-DB	CAGTAGCTCATTTTTGTAAAAGCCTAATTATTGTGCGTGT<br>CCAAAAGACTTTCCTCAAAAGCAAATAAAGAAAAAAAATC<br>TTTGCGTAATTATTCTATGATTACTTTGATGCGTACGTGA<br>ATGGCCATGGGTAGGAGGCAACCAAGTGATTCCCACCTA<br>GCTAGCTTTTCTCCTATTTAAAGCACCACAACTCCCTTCG<br>TTCCTCTCCAAGAGTTTTCAGCCAACACATTGAACTCTTC<br>TTCAGAGCTCTCCCTTCCCTCCACAAAGGGGTCTAGGGT<br>TAGAGTGTGTGTGTCTGTGACAAGTTCCAAGCTAGCAAC<br>AACAAGCTCAATTCCTTGCTTGTTTGCTTCCATATTACAC<br>TACATCTCTTCCCTTCAATTACCCCCCTTTTAGCACACAA<br>AA
SWEET 13	BRRI_Dhan_29	this work	CAGTAGCTCATTTTTGTAAAAGCCTAATTATTGTGCGTGT<br>CCAAAAGACTTTCCTCAAAAGCAAATAAAGAAAAAAAATC<br>TTTGCGTAATTATTCTATGATTACTTTGATGCGTACGTGA<br>ATGGCCATGGGTAGGAGGCAACCAAGTGATTCCCACCTA<br>GCTAGCTTTGGTCCTATATAAAAGCACCACAACTCCCTTC

			GTTCCTCTCCAAGAGTTTTCAGCCAACACATTGAACTCTT<br>CTTCAGAGCTCTCCCTTCCCTCCACAAAGGGGTCTAGGG<br>TTAGAGTGTGTGTGTCTGTGACAAGTTCCAAGCTAGCAA<br>CAACAAGCTCAATTCCTTGCTTGTTTGCTTCCATATTACA<br>CTACATCTCTTCCCTTCAATTACCCCCCTTTTAGCACACA<br>AAA
SWEET 13	Komboka	this work	CAGTAGCTCATTTTTGTAAAAGCCTAATTATTGTGCGTGT<br>CCAAAAGACTTTCCTCAAAAGCAAATAAAGAAAAAAAATC<br>TTTGCATAATTATTCTATGATTACTTTGATGCGTACGTGAA<br>TGGCCATGGGTAGGAGGCAACCAAGTGATTCGCACCTA<br>GCTAGCTTTTCTCCTATATAAGCACCACAACTCCCTTCAT<br>TCCTCTCCAAGAGTTTTCAGCCAACACATTGAACTCTTCT<br>TCAGAGCTCTCCCTTCCCTCCACAAAGGGGTCTAGGGTT<br>AGAGTGTGTGTGTCTGTGACAAGTTCCAAGCTAGCAACA<br>ACAAGCTCAATTCCTTGCTTGTTTGCTTCCATATTACACT<br>ACATCTCTTCCCTTCAATTACCCCCCTTTTAGCACACAAA<br>A
SWEET 13	BRRI_Dhan_28	this work	CAGTAGCTCATTTTTGTAAAAGCCTAATTATTGTGCGTGT<br>CCAAAAGACTTTCCTCAAAAGCAAATAAAGAAAAAAAATC<br>TTTGCATAATTATTCTATGATTACTTTGATGCGTACGTGAA<br>TGGCCATGGGTAGGAGGCAACCAAGTGATTCGCACCTA<br>GCTAGCTTTTCTCCTATATAAGCACCACAACTCCCTTCAT<br>TCCTCTCCAAGAGTTTTCAGCCAACACATTGAACTCTTCT<br>TCAGAGCTCTCCCTTCCCTCCACAAAGGGGTCTAGGGTT<br>AGAGTGTGTGTGTCTGTGACAAGTTCCAAGCTAGCAACA<br>ACAAGCTCAATTCCTTGCTTGTTTGCTTCCATATTACACT<br>ACATCTCTTCCCTTCAATTACCCCCCTTTTAGCACACAAA<br>A
SWEET 13	CX133	3k-DB	CAGTAGCTCATTTTTGTAAAAGCCTAATTATTGTGCGTGT<br>CCAAAAGACTTTCCTCAAAAGCAAATAAAGAAAAAAAATC<br>TTTGCATAATTATTCTATGATTACTTTGATGCGTACGTGAA<br>TGGCCATGGGTAGGAGGCAACCAAGTGATTCGCACCTA<br>GCTAGCTTTTCTCCTATATAAGCACCACAACTCCCTTCAT<br>TCCTCTCCAAGAGTTTTCAGCCAACACATTGAACTCTTCT<br>TCAGAGCTCTCCCTTCCCTCCACAAAGGGGTCTAGGGTT<br>AGAGTGTGTGTGTCTGTGACAAGTTCCAAGCTAGCAACA<br>ACAAGCTCAATTCCTTGCTTGTTTGCTTCCATATTACACT<br>ACATCTCTTCCCTTCAATTACCCCCCTTTTAGCACACAAA<br>A
SWEET 13	IRIS_313-11277	3k-DB	CAGTAGCTCATTTTTGTAAAAGCCTAATTATTGTGCGTGT<br>CCAAAAGACTTTCCTCAAAAGCAAATAAAGAAAAAAAATC<br>TTTGCATAATTATTCTATGATTACTTTGATGCGTACGTGAA<br>TGGCCATGGGTAGGAGGCAACCAAGTGATTCGCACCTA<br>GCTAGCTTTTCTCCTATATATAGCACCACAACTCCCTTCA<br>TTCCTCTCCAAGAGTTTTCAGCCAACACATTGAACTCTTC<br>TTCAGAGCTCTCCCTTCCCTCCACAAAGGGGTCTAGGGT<br>TAGAGTGTGTGTGTCTGTGACAAGTTCCAAGCTAGCAAC<br>AACAAGCTCAATTCCTTGCTTGTTTGCTTCCATATTACAC<br>TACATCTCTTCCCTTCAATTACCCCCCTTTTAGCACACAA<br>AA

SWEET 13	IRIS_313 -10129	3k-DB	CAGTAGCTCATTTTTGTAAAAGCCTAATTATTGTGCGTGT<br>CCAAAAGACTTTCCTCAAAAGCAAATAAAGAAAAAAAATC<br>TTTGCATAATTATTCTATGATTACTTTGATGCGTACGTGAA<br>TGGCCATGGGTAGGAGGCAACCAAGTGATTCGCACCTA<br>GCTAGCTTTTCTCCTATATATAGCACCACAACTCCCTTCA<br>TTCCTCTCCAAGAGTTTTCAGCCAACACATTGAACTCTTC<br>TTCAGAGCTCTCCCTTCCCTCCACAAAGGGGTCTAGGGT<br>TAGAGTGTGTGTGTCTGTGACAAGTTCCAAGCTAGCAAC<br>AACAAGCTCAATTCCTTGCTTGTTTGCTTCCATATTACAC<br>TACATCTCTTCCCTTCAATTACCCCCCTTTTAGCACACAA<br>AA
SWEET 13	IRIS_313 -10148	3k-DB	CAGTAGCTCATTTTTGTAAAAGCCTAATTATTGTGCGTGT<br>CCAAAAGACTTTCCTCAAAAGCAAATAAAGAAAAAAAATC<br>TTTGCATAATTATTCTATGATTACTTTGATGCGTACGTGAA<br>TGGCCATGGGTAGGAGGCAACCAAGTGATTCGCACCTA<br>GCTAGCTTTTCTCCTATATAAGCACCACAACTCCCTTCAT<br>TCCTCTCCAAGAGTTTTCAGCCAACACATTGAACTCTTCT<br>TCAGAGCTCTCCCTTCCCTCCACAAAGGGGTCTAGGGTT<br>AGAGTGTGTGTGTCTGTGACAAGTTCCAAGCTAGCAACA<br>ACAAGCTCAATTCCTTGCTTGTTTGCTTCCATATTACACT<br>ACATCTCTTCCCTTCAATTACCCCCCTTTTAGCACACAAA<br>A
SWEET 13	IRIS_313 -10211	3k-DB	CAGTAGCTCATTTTTGTAAAAGCCTAATTATTGTGCGTGT<br>CCAAAAGACTTTCCTCAAAAGCAAATAAAGAAAAAAAATC<br>TTTGCATAATTATTCTATGATTACTTTGATGCGTACGTGAA<br>TGGCCATGGGTAGGAGGCAACCAAGTGATTCGCACCTA<br>GCTAGCTTTTCTCCTATATAAGCACCACAACTCCCTTCAT<br>TCCTCTCCAAGAGTTTTCAGCCAACACATTGAACTCTTCT<br>TCAGAGCTCTCCCTTCCCTCCACAAAGGGGGTCTAGGGT<br>TAGAGTGTGTGTGTCTGTGACAAGTTCCAAGCTAGCAAC<br>AACAAGCTCAATTCCTTGCTTGTTTGCTTCCATATTACAC<br>TACATCTCTTCCCTTCAATTACCCCCCTTTTAGCACACAA<br>AA
SWEET 13	Kitaake	this work	CAGTAGCTCATTTTTGTAAAAGCCTAATTATTGTGCGTGT<br>CCAAAAGACTTTCCTCAAAAGCAAATAAAGAAAAAAAATC<br>TTTGCATAATTATTCTATGATTACTTTGATGCGTACGTGAA<br>TGGCCATGGGTAGGAGGCAACCAAGTGATTCCCACCTA<br>GCTAGCTTTGCTCCTATATAAAGCACCACAACTCCCTTCA<br>TTCCTCTCCAAGAGTTTTCAGCCAACACATTGAACTCTTC<br>TTCAGAGCTCTCCCTTCCCTCCACAAAGGGGGTCTAGGG<br>TTAGAGTGTGTGTGTCTGTGACAAGTTCCAAGCTAGCAA<br>CAACAAGCTCAATTCCTTGCTTGTTTGCTTCCATATTACA<br>CTACATCTCTTCCCTTCAATTACCCCCCTTTTAGCACACA<br>AAA
SWEET 13	Nipponba re	3k-DB	CAGTAGCTCATTTTTGTAAAAGCCTAATTATTGTGCGTGT<br>CCAAAAGACTTTCCTCAAAAGCAAATAAAGAAAAAAAATC<br>TTTGCATAATTATTCTATGATTACTTTGATGCGTACGTGAA<br>TGGCCATGGGTAGGAGGCAACCAAGTGATTCCCACCTA<br>GCTAGCTTTGCTCCTATATAAAGCACCACAACTCCCTTCA<br>TTCCTCTCCAAGAGTTTTCAGCCAACACATTGAACTCTTC<br>TTCAGAGCTCTCCCTTCCCTCCACAAAGGGGGTCTAGGG

			TTAGAGTGTGTGTGTCTGTGACAAGTTCCAAGCTAGCAA<br>CAACAAGCTCAATTCCTTGCTTGTTTGCTTCCATATTACA<br>CTACATCTCTTCCCTTCAATTACCCCCCTTTTAGCACACA<br>AAA
SWEET 13	Samba Mahsuri	this work	CAGTAGCTCATTTTTGTAAAAGCCTAATTATTGTGCGTGT<br>CCAAAAGACTTTCCTCAAAAGCAAATAAAGAAAAAAAATC<br>TTTGCATAATTATTCTATGATTACTTTGATGCGTACGTGAA<br>TGGCCATGGGTAGGAGGCAACCAAGTGATTCCCACCTA<br>GCTAGCTTTGCTCCTATATAAAGCACCACAACTCCCTTCA<br>TTCCTCTCCAAGAGTTTTCAGCCAACACATTGAACTCTTC<br>TTCAGAGCTCTCCCTTCCCTCCACAAAGGGGGTCTAGGG<br>TTAGAGTGTGTGTGTCTGTGACAAGTTCCAAGCTAGCAA<br>CAACAAGCTCAATTCCTTGCTTGTTTGCTTCCATATTACA<br>CTACATCTCTTCCCTTCAATTACCCCCCTTTTAGCACACA<br>AAA
SWEET 13	IRIS_313 -10521	3k-DB	CAGTAGCTCATTTTTGTAAAAGCCTAATTATTGTGCGTGT<br>CCAAAAGACTTTCCTCAAAAGCAAATAAAGAAAAAAAATC<br>TTTGCATAATTATTCTATGATTACTTTGATGCGTACGTGAA<br>TGGCCATGGGTAGGAGGCAACCAAGTGATTCCCACCTA<br>GCTAGCTTTGCTCCTATATAAAGCACCAACTCCCTTCATT<br>CCTCTCCAAGAGTTTTCAGCCAACACATTGAACTCTTCTT<br>CAGAGCTCTCCCTTCCCTCCACAAAGGGGGTCTAGGGTT<br>AGAGTGTGTGTGTCTGTGACAAGTTCCAAGCTAGCAACA<br>ACAAGCTCAATTCCTTGCTTGTTTGCTTCCATATTACACT<br>ACATCTCTTCCCTTCAATTACCCCCCTTTTAGCACACAAA<br>A
SWEET 13	IRIS_313 -10476	3k-DB	CAGTAGCTCATTTTTGTAAAAGCCTAATTATTGTGCGTGT<br>CCAAAAGACTTTCCTCAAAAGCAAATAAAGAAAAAAAATC<br>TTTGCATAATTATTCTATGATTACTTTGATGCGTACGTGAA<br>TGGCCATGGGTAGGAGGCAACCAAGTGATTCCCACCTA<br>GCTAGCTTTGCTCCTATATAAAGCACCAACTCCCTTCATT<br>CCTCTCCAAGAGTTTTCAGCCAACACATTGAACTCTTCTT<br>CAGAGCTCTCCCTTCCCTCCACAAAGGGGGTCTAGGGTT<br>AGAGTGTGTGTGTCTGTGACAAGTTCCAAGCTAGCAACA<br>ACAAGCTCAATTCCTTGCTTGTTTGCTTCCATATTACACT<br>ACATCTCTTCCCTTCAATTACCCCCCTTTTAGCACACAAA<br>A
SWEET 13	IRIS_313 -11521	3k-DB	CAGTAGCTCATTTTTTGTAAAAGCCTAATTATTGTGCGTG<br>TCCAAAAGACTTTCCGGATCAAAAGCAAATAAAGAAAAAA<br>AAAATCTCTGCATAATTATTCTATGATTACTTTGATGTGTA<br>CGTGAATGGCCATGGGTAGGAGGCAACCAAGTGATTCC<br>CACCTAGCTAGCTTTGCTCCTATATAAAGCACCACAACAC<br>CCTTCATTCCTCTCCAAGAGTTTTCAACCAACACATTGAA<br>CTCTTCTTCAGAGCTCTCCCTTCCCTCCACAAAGGGGTC<br>AAGGGTTAGAGTGTGTGTGAGAGAAGTTCCAAGCTAACA<br>ACACAACAAGCTCAATTCCTTGCTTGTTTGCTTCAAGTTT<br>GCAACCATCTTACACTGCATCTCTTCCCTTCAATTACCCC<br>CCTTTTAGCACACAAAA
SWEET 13	CX145	3k-DB	CAGTAGCTCATTTTTTGTAAAAGCCTAATTATTGTGCGTG<br>TCCAAAAGACTTTCCGGATCAAAAGCAAATAAAGAAAAAA<br>AAATCTCTGCATAATTATTCTATGATTACTTTGATGTGTAC

			GTGAATGGCCATGGGTAGGAGGCAACCAAGTGATTCCC ACCTAGCTAGCTTTGCTCCTATATAAAGCACCACAACACC CTTCATTCCTCTCCAAGAGTTTTCAACCAACACATTGAAC TCTTCTTCAGAGTTCTCCCTTCCCTCCACAAAGGGGTCAA GGGTTAGAGTGTGTGTGAGAGAAGTTCCAAGCTAACAAC ACAACAAGCTCAATTCCTTGCTTGTTTGCTTCCATCTTAC ACTGCATCTCTTCCCTTCAATTACCCCCCTTTTAGCACAC AAAA
SWEET 14	BRRI Dhan 29	this work	AGTTTGTGTGTGCAGCTATATTGCCTATTGGTGTCCAGG GTCACACACCATAAGGGCATGCATGTCAGCAGCTGGTCA TGTGTGCCTTTTCATTCCCTTCTTCCTTCCTAGCACTATAT AAACCCCCTCCAACCAGGTGCTAAGCTCATCAAGCCTTC AAGCAAAGCAAACTCAAGTAGTAGCTGATTACCAGCTCTT CTCTCTTCTCATTGAGAAGAGGGAATTAAGTTTTGATCTC TGCTTTATTGCCTGATCATCCTCTTGTTACTTGCAAGCAA GAACAGTAGTGTACTGTGCCTCATTGATCTCCTCTCACCA AACTCTCTCTCTCTCTCTCTCATATTCCGAGCTAGCTAGT TAATCAAGATCTTGCTGCA
SWEET 14	Samba Mahsuri	this work	AGTTTGTGTGTGCAGCTATATTGCCTATTGGTGTCCAGG GTCACACACCATAAGGGCATGCATGTCAGCAGCTGGTCA TGTGTGCCTTTTCATTCCCTTCTTCCTTCCTAGCACTATAT AAACCCCCTCCAACCAGGTGCTAAGCTCATCAAGCCTTC AAGCAAAGCAAACTCAAGTAGTAGCTGATTACCAGCTCTT CTCTCTTCTCATTGAGAAGAGGGAATTAAGTTTTGATCTC TGCTTTATTGCCTGATCATCCTCTTGTTACTTGCAAGCAA GAACAGTAGTGTACTGTGCCTCATTGATCTCCTCTCACCA AACTCTCTCTCTCTCTCTCTCATATTCCGAGCTAGCTAGTTA ATCAAGATCTTGCTGCA
SWEET 14	BRRI Dhan 28	this work	AGTTTGTGTGTGCAGCTATATTACCTATTGGTGTCCAGG GTCACACACCATAAGGGCATGCATGTCAGCAGCTGGTCA TGTGTGCCTTTTCATTCCCTTCTTCCTTCCTAGCACTATAT AAACCCCCTCCAACCAGGTGCTAAGCTCATCAAGCCTTC AAGCAAAGCAAACTCAAGTAGTAGCTGATTACCAGCTCTT CTCTCTTCTCATTGAGAAGAGGGAATTAAGTTTTGATCTC TGCTTTATTGCCTGATCATCCTCTTGTTACTTGCAAGCAA GAACAGTAGTGTACTGTGCCTCATTGATCTCCTCTCACCA AACTCTCTCTCTCTCTCTCTCATATTCCGAGCTAGCTAGTTA ATCAAGATCTTGCTGCA
SWEET 14	IRIS_313 -10814	3k-DB	AGTTTGTGTGTGCAGCTATATTACCTATTGGTGTCCAGG GTCACACACCATAAGGGCATGCATGTCAGCAGCTGGTCA TGTGTGCCTTTTCATTCCCTTCTTCCTTCCTAGCACTATAT AAACCCCCTCCAACCAGGTAGCTAAGCTCATCAAGCCTT CAAGCAAAGCAAACTCAAGTAGTAGCTGATTACCAGCTC TTCTCTCTTCTCATTGAGAAGAGGGAATTAAGTTTTGATC TCTGCTTTATTGCCTGATCATCCTCTTGTTACTTGCAAGC AAGAACAGTAGTGTACTGTGCCTCATTGATCTCCTCTCAC CAAACTCTCTCTCTCTCTCTCTCATATTCCGAGCTAGCTAGT TAATCAAGATCTTGCTGCA
SWEET	IRIS_313	3k-DB	AGTTTGTGTGTGCAGCTATATTACCTATTGGTGTCCAGG

14	-11317		GTCACACACCATAAGGGCATGCATGTCAGCAGCTGGTCATGTGTGCCTTTTCATTCCCTTCTTCCTTCCTAGCACTATATAAACCCCCTCCAACCAGGTAGCTAAGCTCATCAAGCCTTCAAGCAAAGCAAACTCAAGTAGTAGCTGATTACCAGCTCTTCTCTCTTCTCATTGAGAAGAGGGAATTAAGTTTTGATCTCTGCTTTATTGCCTGATCATCCTCTTGTTACTTGCAAGCAAGAACAGTAGTGTACTGTGCCTCATTGATCTCCTCTCACCAAACTCTCTCTCTCTCTCTCATATTCCGAGCTAGCTAGTTAATCAAGATCTTGCTGCA
SWEET 14	Komboka	this work	AGTTTGTGTGTGCAGCTATATTACCTATTGGTGTCCAGGGTCACACACCATAAGGGCATGCATGTCAGCAGCTGGTCATGTGTGCCTTTTCATTCCCTTCTTCCTTCCTAGCACTATATAAACCCCCTCCAACCAGGTGCTAAGCTCATCAAGCCTTCAAGCAAAGCAAACTCAAGTAGTAGCTGATTACCAGCTCTTCTCTCTTCTCATTGAGAAGAGGGAATTAAGTTTTGATCTCTGCTTTATTGCCTGATCATCCTCTTGTTACTTGCAAGCAAGAACAGTAGTGTACTGTGCCTCATTGATCTCCTCTCACCAAACTCTCTCTCTCTCTCTCATATTCCGAGCTAGCTAGTTAATCAAGATCTTGCTGCA
SWEET 14	Nipponbare	3k-DB	AGTTTGTGTGTGCAGCTATATTGCCTATTGGTGTCCAGGGTCACACACCATAAGGGCATGCATGTCAGCAGCTGGTCATGTGTGCCTTTTCATTCCCTTCTTCCTTCCTAGCACTATATAAACCCCCTCCAACCAGGTGCTAAGCTCATCAAGCCTTCAAGCAAAGCAAACTCAAGTAGTAGCTGATTACCAGCTCTTCTCTCTTCTCATTGAGAAGAGGGAATTAAGTTTTGATCTCTGCTTTATTGCCTGATCATCCTCTTGTTACTTGCAAGCAAGAACAGTAGTGTACTGTGCCTCATTGATCTCCTCCCACCAAACTCTCTCTCTCTCTCTCATATTCCGAGCTAGCTAGTTAATCAAGATCTTGCTGCA
SWEET 14	MTU1010	this work	AGTTTGTGTGTGCAGCTATATTGCCTATTGGTGTCCAGGGTCACACACCATAAGGGCATGCATGTCAGCAGCTGGTCATGTGTGCCTTTTCATTCCCTTCTTCCTTCCTAGCACTATATAAACCCCCTCCAACCAGGTGCTAAGCTCATCAAGCCTTCAAGCAAAGCAAACTCAAGTAGTAGCTGATTACCAGCTCTTCTCTCTTCTCATTGAGAAGAGGGAATTAAGTTTTGATCTCTGCTTTATTGCCTGATCATCCTCTTGTTACTTGCAAGCAAGAACAGTAGTGTACTGTGCCTCATTGATCTCCTCCCACCAAACTCTCTCTCTCTCTCTCATATTCCGAGCTAGCTAGTTAATCAAGATCTTGCTGCA
SWEET 14	Kitaake	this work	AGTTTGTGTGTGCAGCTATATTGCCTATTGGTGTCCAGGGTCACACACCATAAGGGCATGCATGTCAGCAGCTGGTCATGTGTGCCTTTTCATTCCCTTCTTCCTTCCTAGCACTATATAAACCCCCTCCAACCAGGTGCTAAGCTCATCAAGCCTTCAAGCAAAGCAAACTCAAGTAGTAGCTGATTACCAGCTCTTCTCTCTTCTCATTGAGAAGAGGGAATTAAGTTTTGATCTCTGCTTTATTGCCTGATCATCCTCTTGTTACTTGCAAGCAAGAACAGTAGTGTACTGTGCCTCATTGATCTCCTCCCACCAAACTCTCTCTCTCTCTCTCATATTCCGAGCTAGCTAGTTAATCAAGATCTTGCTGCA
SWEET 14	CX371	3k-DB	AGTTTGTGTGTGCAGCTATATTGCCTATTGGTGTCCAGGGTCACACACCATAAGGGCATGCATGTCAGCATCTGGTCA

			TGTGTGCCTTTTCATTCCCTTCTTCCTTCCTAGCACTATAT AAACCCCCTCATCAAGCCTTCAAGCAAAGCAAACTCAAG TAGTAGCTGATTACCAGCTCTTCTCTTCTCATTGAGAAGA GGGAATTAAGTTTTGATCTCTGCTTTATTGCCTGATCATC CCTCTTGCTACTTGCAAGCAAGAACAGTAGTGTACTGTA CCTCATTGATCTCCTCTCACCAAACTCTCTCTCTCATATT CTGATCTAGCTAGTTAATCAAGATCTTGCTGCA
SWEET 14	IR64	this work	AGTTTGTGTGTGCAGCTATATTGCCTATTGGTGTCCAGG GTCACACACCATAAGGGCATGCATGTCAGCAGCTGGTCA TGTGTGCCTTTTCATTCCCTTCTTCCTTCCTAGCACTATAT AAACCCCCTCCAACCAGGTGCTAAGCTCATCAAGCCTTC AAGCAAAGCAAACTCAAGTAGTAGCTGATTACCAGCTCTT CTCTCTTCTCATTGAGAAGAGGGAATTAAGTTTTGATCTC TGCTTTATTGCCTGATCATCCTCTTGTTACTTGCAAGCAA GAACAGTAGTGTACTGTGCCTCATTGATCTCCTCTCACCA AACTCTCTCTCTCTCTCTCTCATATTCCGAGCTAGCTAGT TAATCAAGATCTTGCTGCA
SWEET 14	CX372	3k-DB	AGTTTGTGTGTGCAGCTATATTGCCTATTGGTGTCCAGG GTCACACACCATAAGGGCATGCATGTCAGCATCTGGTCA TGTGTGCCTTTTCATTCCCTTCTTCCTTCCTAGCACTATAT AAACCCCCTCATCAAGCCTTCAAGCAAAGCAAACTCAAG TAGTAGCTGATTACCAGCTCTTCTCTTCTCATTGAGAAGA GGGAATTAAGTTTTGATCTCTGCTTTATTGCCTGATCATC CCTCTTGCTACTTGCAAGCAAGAACAGTAGTGTACTGTA CCTCATTGATCTCCTCTCACCAAACTCTCTCTCTCATATT CTGATCTAGCTAGTTAATCAAGATCTTGCTGCA

Supplementary Table 5. List of PCR primers and their applications.

Primer	Sequence (5' to 3')	Usage
SWEET11-ko-F1	TCTGGCTAGTTTCTAGCTGGT	Genotype *SWEET11* knockout mutations through PCR and sequencing
SWEET11-ko-R1	CAGCAATTAACCTGAATTTGA	
SWEET13-ko-F1	ACACATTGAACTCTTCTTCAGAG	Genotype *SWEET13* knockout mutations through PCR and sequencing
SWEET13-ko-R1	AATGAAGGGAATGTCTTAGTTGT	
SWEET14-ko-F1	AAGCCTTCAAGCAAAGCAAA	Genotype *SWEET14* knockout mutations through PCR and sequencing
SWEET14-ko-R1	TTGGCAACAAAAGTGGAACA	
SWEET11-RT-F	GGGATTTCTGGCTAGTTTCT	RT-PCR for *SWEET11*
SWEET11-RT-R	CGAGGTAGAGGACGATGTAG	
SWEET13-RT-F	AGAGTTTTCAGCCAACACAT	RT-PCR for *SWEET13*
SWEET13-RT-R	TGTAGATCCGGTAGAACGTC	
SWEET14-RT-F	TATTGCCTGATCATCCTCTT	RT-PCR for *SWEET14*
SWEET14-RT-R	GTGAACATCTTGGCCTTCT	
UBI1-F	AGAAGGAGTCCACCCTCCACC	Internal Control for RT-PCR
UBI1-R	GCATCCAGCACAGTAAAACACG	
gSWEET13-XbaI-F	TCTAGATCGTAGAAAATGAGACGA	Translational GUSplus fusion construction for SWEET13
gSWEET13-KpnI-F	GGTACCCACGGCGGCCACCTCGA	
gSWEET14-BamHI-F	GGATCCTAGGTTAACGCTATGTTTTGA	Translational GUSplus fusion construction for SWEET14
gSWEET14-XmaI-F	CCCGGGGGTGCAGCTACCTTCTTCTC	

Supplementary Table 6. DNA Sanger sequencing results for *SWEET* gene knockouts

**Knockout allele**	*sweet11* (-1 bp)
**Raw sequencing**	>E08_BY8254_8N3F4_993226.seq | Sequence #1 of 1 downloaded on Fri Jun 17 11:28:19 CDT 2016<br>NNNNNNTNNNANCGACTACTCTGCATTCTTTTCTGACGATAGAAGTCGATTGA<br>TGGATCACCCAACATGTTACTAATTAAGTTGCATCATTGTCCATGGTTGTACAT<br>CCTTCTACAAATAAAACTACACAAATCAAGAAAATTTTCAATAACATTTCAACTA<br>TTGTAACAAGTAAAAGAAACCTATATGAGAGCTCCAGCTCTCCAAATGGCAAC<br>AGACACACTGAGTGGTCATACGTGTCATATTGCCCCTCAGTTATGCATTCATA<br>TGACCACATATTCAGAGTAGTGGAGAGAGGGACAGATCTAGAGGTAGAAAAA<br>GAAAATTCATATAAATGATATATCAGAGTGAAAAAGAAATATCAAGCACAAGAA<br>AAAAAAGCAAAGGTTAGATATGCATCTCCCCCTACTGTACACCACCAAAAGTG<br>GAGGGTCTCCAACTATATAAACACTGAGCCATGGCCAAGGCCAAACCACACA<br>TGCAGTTGTAGTAGCACTTAAGCCTTCCTCTCTAGCTAGCATCTCTTGTGTCA<br>GGAAGTTGGAAGGGATTTCTGGCTAGTTTCTAGCTGGTGTCTCCTCTCCTCTT<br>CCTAACCTTCTCACTGATTAACACCTTAGAGTTAGTTAATAACCTTCATCACCA<br>GTAGCAATGCAGGAGGTTTCTTGTCCATGGCTAACCCGGCGGTCACCCTCTC<br>CGGTGTTGCAGGTAAAGCATGCAACCAATGCATAATGCTCAAACTTAATTTCA<br>TCATCATCATCATCATCATCATCTTCACAGCCATGATCATCCATGGACAAATG<br>CAACTGAAGATCATTTTAGTTTTCATATGCTAATG
**Knockout allele**	*sweet13* (-10 bp)
**Raw sequencing**	>A02_by4376_swt13-f_877808.seq | Sequence #7 of 10 downloaded on Tue Mar 18 12:12:38 CDT 2014<br>NNNNNNNNNNNNNNNNNNNNNNNNANCTAGCNCNACAAGCTCAATTCCTTGCT<br>TGTTTGCTTCCATATTACACTACATCTCTTCCCTTCAATTACCCCCCTTTTAGC<br>ACACAAAAATGGCTGGCCTGTCCCTGCAGCTTTTGCCTTCGGCCTCCTTGGT<br>ATATCATCATCACCTACCACAACTAAGACATTCCCTTCATTGCCAACATTTTAC<br>TTCTTTTTATTAGAAACCATTGAGTTTGTACATATAAGCTCAGAGTTATTACTTG<br>CTGGCAGATACGCATAGCTAGCGACGACATTTAGTGAGGATTACACTTTAGT<br>GGTCATCATATATAAAATAATAAAATTGTTGTGTTTCTTTATTTGTTTTTTATTTT<br>CTAGCTAGCTTCTTTCAGTTATTTTCAACAAAAAGAATTTCTTCTAGCACTTTTA<br>TTTACAGCAGTTTATTATGTCTATTTGGGCATATTAATTAGCTACTAGCGCATA<br>AGCTAAAAACCCTACTAGCCTTCCGAAGGCAAANTNGANNGNTNNNNNGCAT<br>CACANGNTCGACGCTCNTCCGAGGNNGCNAAGCCCGNCGGGACNATAAAGA<br>TACCATGGNTTCCNCCTGANANNTCCCTCTTGAANTCTCCTGANCCGACCCT<br>GCCGCTTANCNNANACCTGTNCNNCTNNCTCCCGNCGGGAANCGTGCCGCT<br>ATCACANANCTCACNCTGTTGGGTATCNCNNTTCGGTGTAGGTCGTTCGCTC<br>CAAGCAGGGGCTGTGTGCACNAAACCCCCCCTTTCAGCCCAACGGCCGCGC<br>CTTATCCAGGTAACTATGNNATATGANTCCAACCCGGTAGGACNCGACTTAAT<br>NNCCTCTGGCGCAGCCACTGGCNANGTGACNANCTNAACNNGNNNTAGCNG<br>TGCTCCGGATNTCNTGAAAGGTGGCACCACTACCCNTTCCCCNNGANAACCG<br>T
**Knockout allele**	*sweet14* (-1 bp)
**Raw sequencing**	> BY8859_RGT48-F_1006267.seq<br>NNNNNNTTANTANNNATGCATTGAGGANAGAGTTATGAAGGAAACAAAAAAAA<br>GCTAGCAGATTGGCACTTTCTGTCATGCATGGGTGCTGATGATTATCTTGTAT<br>CTAATTTAATCAATCCCATGGCTGTGATTGATCAGGAATAGTTTGTGTGTGCA<br>GCTATATTGCCTATTGGTGTCCAGGGTCACACACCATAAGGGCATGCATGTC<br>AGCAGCTGGTCATGTGTGCCTTTTCATTCCCTTCTTCCTTCCTAGCACTATATA<br>AACCCCCTCCAACCAGGTGCTAAGCTCATCAAGCCTTCAAGCAAAGCAAACT<br>CAAGTAGTAGCTGATTACCAGCTCTTCTCTCTTCTCATTGAGAAGAGGGAATT<br>AAGTTTTGATCTCTGCTTTATTGCCTGATCATCCTCTTGTTACTTGCAAGCAAG<br>AACAGTAGTGTACTGTGCCTCATTGATCTCCTCCCACCAAACTCTCTCTCTCT<br>CTCTCATATTCCGAGCTAGCTAGTTAATCAAGATCTTGCTGCAATGGCTGGCA

	TGTCTCTTCAGCTCCCTGGGCCTTCGCCTTTGGTCTCCTAGGTGTGTTGCCTT TGATCTGATCCAAGGAATTCTCTTGAGAATTAATCTTGCATGGTTATTTACTTT TGTTGTTATTATTCTCTACATTTTTAATCATGTACTTTTCCATGTTCCACTTTTG TTGCCAAAGCTACTATATTTTTCCTACCAATTCATCCAAAACTACTATATTATAG CAAGGCAGCTAGTGGATCGACTTTGCACTTTTGGATGCAATTGTGAGTGGTTT ACATTAGAGGGGCCATGCATAGCCAAATAAATTGTTTGGCAAAATATGATTTC CCTTGTAGTTAGCACAATATTGTGATCTTTCATTCCTTTACTCTTTTTGTTCCAC CACCCTCATTATTGCAAAGCCCCCTCTTTTAGAACCAAACTAAAGATAAGAAA CATTTGATTCATCATTAGAGGAGATTAGATATACATACNTACTACACATGCATT GCTGTGATAAGCCTAGCCGGGCACAGAAAGAGCCAGACAAAGA
**Knockout allele**	*sweet11;13 (-1 bp;+1 bp)*
**Raw sequencing**	>G01_11-2_1226504.seq | Sequence #7 of 96 downloaded on Thu May 31 08:19:48 CDT 2018 (*sweet11*, 1 bp homozygous deletion) NNNNNNANNNNGATCATGGAGACAGAAAGCTCAAGAACACAAGGACATAAAA AAAAGGAGAAGATTTTTTAACAACATTGCCCTGCTGCACTGTGTGAACTGCTG AACAGATATGCATTCCTAGAAGCATCAAAAGCGTGTGTGTGCTGTGCACAATG CAATGGTGCAGACAACAACTAGTGTATGGAGAAATTAAACAGCAATTAACCTG AATTTGATCATTAGCATATGAAAACTAAAATGATCTTCAGTTGCATTTGTCCAT GGATGATCATGGCTGTGAAGATGATGATGATGATGATGATGATGAAATTAAGT TTGAGCATTATGCATTGGTTGCATGCTTTACCTGCAACACCGGAGAGGGTGA CCGCCGGGTTACCATGGACAAGAAACCTCCTGCCATTGCTACTGGTGATGAA GGTTATTAACTAACTCTAAGGTGTTAATCAGTGAGAAAATA
	>B09_13-7_1226563.seq | Sequence #66 of 96 downloaded on Thu May 31 08:19:48 CDT 2018 (*sweet13*, 1 bp homozygous insertion) NNNNNNNNNNAGTANTGATTTCTACATGATATATACATATATAGCTTTTGTGTA ATTTTAATTCCCAATCCGCACCGCGTTCCGTATCCGATAATATATGTGCATAAT ATTTTATGCAGCCCGACGTTCTACCGGATCTACAAGAGCAAGTCGATCGGAG GGGTTCCAGTCGGTGCCGTACGTGGTGGCGCTCTTCAGCGCCATGCTGTGG ATCTTCTACGCGCTGATCAAGTCCAACGAGGCCCTCCTCATCACCATCAACG CCGCCGGTTGCGTCATCGAGACCATCTACATCGTCATGTACCTCGCCTACGC CCCCAAGAAGGCCAAGGTTCGATCCATGTCATCCAAACCCTAGCACTACCAC AGAAACCCTATTTCGATGCCGGTGGGGACTTGGGAACCACCCTTTGATTCTC GAGTCGCAACCGGCACCGATGAGAGGCCCCTTACAGGTGTCATCGGTTGTG AACAAGAACCGGCACCTATATAAGATTGTTCCATATTTACCGTTTTGTAGTAGT AAAATATAATAATTGACAAGTATATATGGTTCGACGTCGTTTTTGCAGGTGTTC ACGACGAAGATCCTGCTGCTGCTGAACGTGGGGGTGTTCGGGGTGATCCTG CTGCTTGGACGCTGAA
**Knockout allele**	*sweet11;14 (-1 bp;+1 bp)*
**Raw sequencing**	>E10_B10264_1151982.seq | Sequence #77 of 96 downloaded on Wed Sep 6 09:46:02 CDT 2017 (*sweet11*, 1 bp homozygous deletion) NNNNNNNNATTNTATGANNANATATTCAGAGTAGTGGAGAGAGGGACAGATC TAGAGGTAGAAAAAGAAAATTCATATAAATGATATATCAGAGTGAAAAAGAAAT ATCAAGCACAAGAAAAAAAAGCAAAGGTTAGATATGCATCTCCCCCTACTGTA CACCACCAAAAGTGGAGGGTCTCCAACTATATAAACACTGAGCCATGGCCAA GGCCAAACCACACATGCAGTTGTAGTAGCACTTAAGCCTTCCTCTCTAGCTAG CATCTCTTGTGTCAGGAAGTTGGAAGGGATTTCTGGCTAGTTTCTAGCTGGTG TCTCCTCTCCTCTTCCTAACCTTCTCACTGATTAACACCTTAGAGTTAGTTAAT AACCTTCATCACCAGTAGCAATGCAGGAGGTTTCTTGTCCATGGCTAACCCG GCGGTCACCCTCTCCGGTGTTGCAGGTAAAGCATGCAACCAATGCATAATGC TCAAACTTAATTTCATCATCATCATCATCATCATCATCTTCACAGCCATGATCA TCCATGGACAAATGCAACTGAAGATCATTTTAGTTTTCATATGCTAATGATCAA ATTCAGGTTAATTGCTGTTTAATTTCTCCATACACTAGTTGTTGTCTGCACCAT TGCATTGTGCACAGCACACACACGCTTTTGATGCTTCTAGGAATGCATATCTG TTCAGCAGTTCACACAGTGCAGCAGGGCAATGTTGTTAAAAAATCTTCTCCTT

	TTTTTTATGTCCTTGTGTTCTTGAGCTTTCTGTCTCCATTGATCTGCTTTTTTCT<br>TGTTTACAAGTGATGG
	>F10_B10265_1151983.seq | Sequence #78 of 96 downloaded on Wed Sep 6 09:46:02 CDT 2017 (*sweet14*, 1 bp homozygous insertion)<br>NNNANNNNTTTNTATCNATGCATTGAGGANAGAGTTATGAAGGAAACAAAAAA<br>AAGCTAGCAGATTGGCACTTTCTGTCATGCATGGGTGCTGATGATTATCTTGT<br>ATCTAATTTAATCAATCCCATGGCTGTGATTGATCAGGAATAGTTTGTGTGTG<br>CAGCTATATTGCCTATTGGTGTCCAGGGTCACACACCATAAGGGCATGCATG<br>TCAGCAGCTGGTCATGTGTGCCTTTTCATTCCCTTCTTCCTTCCTAGCACTAT<br>ATAAACCCCCTCCAACCAGGTGCTAAGCTCATCAAGCCTTCAAGCAAAGCAA<br>ACTCAAGTAGTAGCTGATTACCAGCTCTTCTCTCTTCTCATTGAGAAGAGGGA<br>ATTAAGTTTTGATCTCTGCTTTATTGCCTGATCATCCTCTTGTTACTTGCAAGC<br>AAGAACAGTAGTGTACTGTGCCTCATTGATCTCCTCCCACCAAACTCTCTCTC<br>TCTCTCTCATATTCCGAGCTAGCTAGTTAATCAAGATCTTGCTGCAATGGCTG<br>GCATGTCTCTTCAGCACCCTGGGCCTTCGCCTTTGGTCTCCTAGGTGTGTTG<br>CCTTTGATCTGATCCAAGGAATTCTCTTGAGAATTAATCTTGCATGGTTATTTA<br>CTTTTGTTGTTATTATTCTCTACATTTTTAATCATGTACTTTTCCATGTTCCACT<br>TTTGTTGCCAAAGCTACTATATTTTTCCTACCAATTCATCCAAAACTACTATATT<br>ATAGCAAGGCAGCTAGTGGATCGACTTTGCACTTTTGGATGCAATTGTGAGT<br>GGTTTACATTAGAGGGGCCATGCATAGCCAAATAAATTGTTTGGCAAAATATG<br>ATTTCCCTTGTAGTTAGCACAATATTGTGATCTTTCATTCCTTTACTCTTTTTGT<br>TCCACCACCCTCATTATTGCAAAGCCCCCTCTTTTAGAACCAAACTAAAGATA<br>AGAAACATTTGATTCATCATTAGAGGAGATTAGATATACNTACATACTACACAT<br>GCATTGCTGTGATAAGCCTAGCCAGGCACAGAAAGAGCCA
**Knockout allele**	sweet13;14 *(+1 bp;+1 bp)*
**Raw sequencing**	>C10_B10262_1151980.seq | Sequence #75 of 96 downloaded on Wed Sep 6 09:46:02 CDT 2017 (*sweet13,* 1 bp homozygous insertion)<br>NNNNNNNTTTNNTTATGCGCTAGTAGCTAATTAATATGCCCAAATAGACATAA<br>TAAACTGCTGTAAATAAAAGTGCTAGAAGAAATTCTTTTTGTTGAAAATAACTG<br>AAAGAAGCTAGCTAGAAAATAAAAAACAAATAAAGAAACACAACAATTTTATTA<br>TTTTATATATGATGACCACTAAAGTGTAATCCTCACTAAATGTCGTCGCTAGCT<br>ATGCGTATCTGCCAGCAAGTAATAACTCTGAGCTTATATGTACAAACTCAATG<br>GTTTCTAATAAAAAGAAGTAAAATGTTGGCAATGAAGGGAATGTCTTAGTTGT<br>GGTAGGTGATGATGATATACCAAGGAGGCCGAAGGCAAAAGCCCAGGGGAT<br>GCTGCAGGGACAGGCCAGCCATTTTTGTGTGCTAAAAGGGGGGTAATTGAAG<br>GGAAGAGATGTAGTGTAATATGGAAGCAAACAAGCAAGGAATTGAGCTTGTT<br>GTTGCTAGCTTGGAACTTGTCACAGACACACACACTCTAACCCTAGACCCCTT<br>TTGGTGGAAGAGAA
	>D10_B10263_1151981.seq | Sequence #76 of 96 downloaded on Wed Sep 6 09:46:02 CDT 2017 (*sweet14,* 1 bp homozygous insertion)<br>NNNNNNNNTNNTANNNATGCATTGAGGANAGAGTTATGAAGGAAACAAAAAA<br>AAGCTAGCAGATTGGCACTTTCTGTCATGCATGGGTGCTGATGATTATCTTGT<br>ATCTAATTTAATCAATCCCATGGCTGTGATTGATCAGGAATAGTTTGTGTGTG<br>CAGCTATATTGCCTATTGGTGTCCAGGGTCACACACCATAAGGGCATGCATG<br>TCAGCAGCTGGTCATGTGTGCCTTTTCATTCCCTTCTTCCTTCCTAGCACTAT<br>ATAAACCCCCTCCAACCAGGTGCTAAGCTCATCAAGCCTTCAAGCAAAGCAA<br>ACTCAAGTAGTAGCTGATTACCAGCTCTTCTCTCTTCTCATTGAGAAGAGGGA<br>ATTAAGTTTTGATCTCTGCTTTATTGCCTGATCATCCTCTTGTTACTTGCAAGC<br>AAGAACAGTAGTGTACTGTGCCTCATTGATCTCCTCCCACCAAACTCTCTCTC<br>TCTCTCTCATATTCCGAGCTAGCTAGTTAATCAAGATCTTGCTGCAATGGCTG<br>GCATGTCTCTTCAGCATTCCCTGGGCCTTCGCCTTTGGTCTCCTAGGTGTGTT<br>GCCTTTGATCTGATCCAAGGAATTCTCTTGAGAATTAATCTTGCATGGTTATTT<br>ACTTTTGTTGTTATTATTCTCTACATTTTTAATCATGTACTTTTCCATGTTCCAC<br>TTTTGTTGCCAAAGCTACTATATTTTTCCTACCAATTCATCCAAAACTACTATAT<br>TATAGCAAGGCAGCTAGTGGATCGACTTTGCACTTTTGGATGCAATTGTGAGT

	GGTTTACATTAGAGGGGCCATGCATAGCCAAATAAATTGTTTGGCAAAATATG<br>ATTTCCCTTGTAGTTAGCACAATATTGTGATCTTTCATTCCTTTACTCTTTTTGT<br>TCCACCACCCTCATTATTGCAAAGCCCCCTCTTTTAGAACCAAACTAAAGATA<br>AGAAACATTTGATTCATCATTAGAGGAGATTAGATATACATACATACTACACAT<br>GCATTGCTGTGATAAGCCTAGCCNGGCACAGAAAGAGCCAAGACAAAAGA
**Knockout allele**	*sweet11;13;14* (-1 bp;+1 bp;+1 bp)
**Raw sequencing**	>G05_BY9336_8N3-R2_1021703.seq | Sequence #2 of 2 downloaded on Mon Jan 30 13:07:38 CST 2017 (*sweet11*, homozygous of 1 bp deletion)<br>NNNNNNNNNNNNNNNATCANTGNNAANCAGAAAGCTCAAGAACACAAGGACAT<br>AAAAAAAAGGAGAAGATTTTTAACAACATTGCCCTGCTGCACTGTGTGAACT<br>GCTGAACAGATATGCATTCCTAGAAGCATCAAAAGCGTGTGTGTGCTGTGCA<br>CAATGCAATGGTGCAGACAACAACTAGTGTATGGAGAAATTAAACAGCAATTA<br>ACCTGAATTTGATCATTAGCATATGAAAACTAAAATGATCTTCAGTTGCATTTG<br>TCCATGGATGATCATGGCTGTGAAGATGATGATGATGATGATGATGATGAAAT<br>TAAGTTTGAGCATTATGCATTGGTTGCATGCTTTACCTGCAACACCGGAGAGG<br>GTGACCGCCGGGTTACCATGGACAAGAAACCTCCTGCCATTGCTACTGGTGA<br>TGAAGGTTATTAACTAACTCTAAGGTGTTAATCAGTGAGAAGGTTAGGAAGAG<br>GAGAGGAGACACCAGCTAGAAACTAGCCAGAAATCCCTTCCAACTTCCTGAC<br>ACAAGAGATGCTAGCTAGAGAGGAAGGCTTAAGTGCTACTACAACTGCATGT<br>GTGGTTTGGCCTTGGCCATGGCTCAGTGTTTATATAGTTGGAGACCCTCCAC<br>TTTTGGTGGTGTACAGTAGGGGGAGATGCATATCTAACCTTTGCTTTTTTTTCT<br>TGTGCTTGATATTTCTTTTTCACTCTGATATATCATTTATATGAATTTTCTTTTTC<br>TACCTCTAGATCTGTCCCTCTCTCCACTACTCTGAATATGTGGTCATATGAAT<br>GCATAACTGAGGGGCAATATGACACTAGGA
	>H03_BY9334_SWT13-F2_1021676.seq | Sequence #1 of 1 downloaded on Mon Jan 30 12:58:17 CST 2017 (*sweet13*, homozygous of 1 bp insertion)<br>NNNNNNNNNAGTACTGATTTCTACATGATATATACATATATAGCTTTTGTGTAA<br>TTTTAATTCCCAATCCGCACCGCGTTCCGTATCCGATAATATATGTGCATAATA<br>TTTTATGCAGCCCGACGTTCTACCGGATCTACAAGAGCAAGTCGATCGGAGG<br>GGTTCCAGTCGGTGCCGTACGTGGTGGCGCTCTTCAGCGCCATGCTGTGGA<br>TCTTCTACGCGCTGATCAAGTCCAACGAGGCCCTCCTCATCACCATCAACGC<br>CGCCGGTTGCGTCATCGAGACCATCTACATCGTCATGTACCTCGCCTACGCC<br>CCCAAGAAGGCCAAGGTTCGATCCATGTCATCCAAACCCTAGCACTACCACA<br>GAAACCCTATTTCGATGCCGGTGGGGACTTGGGAACCACCCTTTGATTCTCG<br>AGTCGCAACCGGCACCGATGAGAGGCCCCTTACAGGTGTCATCGGTTGTGA<br>ACAAGAACCGGCACCTATATAAGATTGTTCCATATTTACCGTTTTGTAGTAGTA<br>AAATATAATAATTGACAAGTATATATGGTTCGACGTCGTTTTTGCAGGTGTTCA<br>CGACGAAGATCCTGCTGCTGCTGAACGTGGGGGTGTTCGGGGTGATCCTG
	>A07_BY9354_SWET14F2_1022612.seq | Sequence #1 of 1 downloaded on Fri Feb 3 11:34:46 CST 2017 (*sweet14*, homozygous of 1 bp insertion)<br>NNNNNNNNNNTTNNNNNNNNNATNNGANNNNATTTTCTTTTCTTTACCACANTT<br>TTACATTGCTCCAGAAAGTACCTTTTATACTAAAATTTCTTATACACCACCAGA<br>AAATTAATGTGGTACTATTGGTACCTTAGCAAAAATGATAAAATTACCTTTTTC<br>TTCAGATAAAAACACTCCCCGTCTCTGTATAACTAAGATGTAGACAACCAATA<br>CACATGTCTAGAGCTAATCCTATCAATTAACATTGCTAGTAAATACAAGGAAAA<br>ATATAAAGAATTAACCAATTGCTTGGATGTCTTGCAGGCCGACGTTCTACAGG<br>ATCTACAAGAGCAAGTCGAACGCAGGGGTTCCAGTCGGTACCCTACGTGGTG<br>GCGCTGTTCAGCGCGATGCTGTGGATCTACTACGCGCTGCTCAAGTCCGAC<br>GAGTGCCTCCTCATCACCATCAACTCCGCTGGCTGCGTCATCGAGACCATCT<br>ACATCGCCGTCTACCTCGTCTACGCCCCCAAGAAGGCCAAGATGTTCACCGC<br>CAAGCTCCTCCTCCTCGTCAACGTCGGCGTCTTCGGCCTCATCCTCCTCCTC<br>ACCCTCCTCCTCTCCGCCGGCGACCGCCGCATCGTGGTTCTTGGTTGGGTCT<br>GCGTTGGCTTCTCCGTCAGCGTCTTCGTCGCCCCCCTTAGCATCATCGTAAG<br>AAACCCTAGAATTGCCTGTAAACAATAATAACTTTCGGTCTCTGCACCAACTA<br>AGGATGCACATATTTACGCACCGCTTTTGCTATCTCTGCAGAGGCTGGTGGT

	GCGCACCAAGAGCGTGGAGTTCATGCCGTTCTCGCTCTCCTTC

Supplementary Table 7. Lesion length for individual, double and triple SWEET^{ko} lines (Kitaake)

Strain	origin	Kitaake	*sweet11*	*sweet13*	*sweet14*	*sweet11; sweet13*	*sweet11; sweet14*	*sweet13; sweet14*	*sweet11; sweet13; sweet14*
ME2	Lab	0.6±0.2	0.6±0.2	0.5±0	1.0±0	2.0±0.4	0.5±0	0.6±0.2	0.5±0
ME2 (*PthXo1*)	Lab	15.3±1.8	0.6±0.2	12.0±1.6	13±0.2	1.3±0.2	0.5±0	13.3±2	0.9±0.2
ME2 (*PthXo2B*)	Lab	14.5±0.6	16.6±2.1	1.0±0	11.4±0.7	0.9±0.3	16.5±1	0.8±0.2	0.8±0.2
ME2 (*PthXo3*)	Lab	15.8±1.2	17.1±1.5	11.8±1.1	2.6±0.3	14.1±2.1	1.9±0.4	1.0±0.2	1.0±0.2
ME2 (*AvrXa7*)	Lab	17.1±1.9	13.9±0.9	13.5±1.6	1.0±0	14.3±0.8	0.6±0.2	0.9±0.3	0.8±0.2
ME2 (*TalC*)	Lab	9.1±2.0	4.6±0.8	3.3±0.5	0.9±0.2	5.5±0.8	0.9±0.2	0.8±0.2	0.8±0.2
ME2 (*TalF*)	Lab	14.0±3.4	13.6±0.6	11.0±1.4	1.0±0.2	12.9±1.1	0.5±0	0.6±0.2	1.4±0.3
PXO99	PHL	15.9±1.3	1.0±0.2	15.6±0.7	16.8±1.6	1.0±0	1.0±0	18.6±1.5	0.8±0.2
PXO86	PHL	16.8±1.1	13.9±0.7	13.8±0.9	0.9±0.2	13.3±0.2	0.9±0.2	1.1±0.4	1.1±0.4
PXO61	PHL	19.5±0.8	15.5±0.8	14.0±0.8	11.0±1.6	17.4±1.1	13.3±0.8	0.9±0.2	1.3±0.3
PXO364	PHL	18.6±0.7	16.9±0.6	13.4±0.9	14.6±1.1	19.3±1.3	16.8±0.7	1.1±0.2	0.8±0.2
PXO404	PHL	19.5±1.0	17.0±0.8	17.3±0.4	13.0±1.2	14.9±1.1	16.5±1.6	0.9±0.2	0.9±0.2
PXO421	PHL	20.3±0.6	18.4±0.7	15.0±1.6	13.1±1.6	14.9±1.7	17.1±0.9	0.9±0.2	1.5±0.4
PXO513	PHL	17.9±0.5	18.3±0.3	15.1±1.3	12.3±1	13.6±2.7	16.3±0.4	1.1±0.2	0.9±0.2
KXO85	Korea	18.1±1.3	16.6±1.1	17.4±1.9	16.1±1.5	13.6±1.2	15.3±1.2	1.1±0.2	0.9±0.2
JW89011	Korea	18.9±1.7	15.8±1.5	15.5±1.0	12.9±1.1	17.0±1.8	11.6±1.4	1.0±0.2	1.4±0.3
T7174	Japan	16.5±1.0	14.4±1.0	12.4±1.0	0.6±0.2	13.1±0.7	1.3±0.2	0.9±0.2	1.5±0.6
XOO2	Thailand	19.5±3.0	18.3±1.2	12.8±0.4	1.5±0.4	14.4±1.1	1.8±0.3	1.5±0.2	1.5±0.4
PbXo7	India	18.9±1.1	0.9±0.2	16.9±1.4	17.6±1.2	0.9±0.2	1.0±0.2	19.9±0.5	1.1±0.4
IXO56	Indonesia	18.9±1.3	18.0±1.2	14.6±0.5	0.9±0.2	16.3±1.2	1.8±0.2	1.5±0.6	0.9±0.2
NXO260	Nepal	16.5±2.0	1.1±0.2	12.9±0.9	18.0±1.6	1.0±0	0.8±0.2	19.4±0.7	1.1±0.4
CIAT1185	Colombia	10.5±2.6	0.8±0.2	10.1±0.9	13.6±0.9	1.6±0.3	1.0±0	14.4±1.7	0.9±0.3
ZHE173	China	15.3±1.0	16.0±0.8	13.9±1.1	1.3±0.3	11.8±0.2	1.0±0	0.9±0.2	1.3±0.5
C1	China	17.1±0.6	16.9±1.3	12.1±2.1	1.4±0.3	13.9±1.7	1.3±0.2	0.8±0.2	0.8±0.2

HB21	China	20.3±1.5	17.5±1.2	17.0±0.2	1.4±0.3	16.5±1.4	2.1±0.3	1.0±0.2	1.6±0.3
Aust-2013	Australia	18.4±1.3	14.9±1.7	11.0±1.2	1.5±0.4	15.5±0.6	1.6±0.3	0.6±0.2	0.9±0.2
Aust-R3	Australia	16.8±1.6	18.0±1.6	14.8±0.8	1.6±0.5	17.3±1.1	2.0±0.2	1.1±0.2	2.1±0.4
AXO1947	Africa	16.4±1.1	15.5±1.0	10.4±1.7	3.5±0.4	11.9±1.7	6.9±0.3	1.4±0.3	0.9±0.3
BAI3	Africa	14.8±1.2	13.1±1.1	10.8±1.4	7.1±1.2	12.0±1.8	8.5±0.5	0.8±0.2	0.9±0.2
MAI1	Africa	12.5±1.0	16.9±2.1	7.9±0.7	4.5±0.4	14.0±1.0	3.1±0.6	0.8±0.2	0.6±0.2

PHL, The Philippines; Red indicates resistance; Blue indicates moderate resistance. Lesion lengths (mean ± s.e.m., n=10 inoculated leaves) were measured 14 days post inoculation. The experiment was repeated independently at least twice with similar results.

Supplementary Table 8. Total number of genome-edited EBE variants in three SWEET genes

promoter	EBE for TALe	Kitaake variants	IR64 variants	Ciherang-Sub1 variants
SWEET11	PthXo1	9	3	3
SWEET13	PthXo2 (PthXo2B)	10	5	6
SWEET14	TalC	12	4	5
SWEET14	TalF	2	0	0
SWEET14	AvrXa7	9	8	4
SWEET14	PthXo3	9	8	4

Supplementary Table 9. Resistance of promoter-edited Kitaake lines

	SWEET11	*SWEET13*	*SWEET14*			Xoo pathogenicity*		
	PthXo1	PthXo2B	TalC	AvrXa7/PthXo3	TalF	PXO99	PXO86	PXO61
Kitaake WT	WT	WT	WT	WT	WT	S	S	S
#11.1-45	-3	-38	-4	-2	WT	R	R	S
#12.2-12	+1	+2	-62		WT	MS	R	R

* Pathogenicity was scored as resistant reaction (R) with lesion lengths < 3 cm, susceptible reaction (S) with length 15-20 cm, and moderately susceptible reaction (MS) with length lesion >8 and <15 cm. The disease assay was repeated three times with similar results.

Supplementary Table 10. Virulence of Xoo strains on promoter variants of mega varieties
reproduced from Oliva, R. *et al. Nat. Biotechnol.*, 2019, doi.org/10.1038/s41587-019-0267-z

<table>
<tr><th colspan="2"></th><th colspan="3">Xoo pathogenicity[1]</th><th>SWEET11</th><th>SWEET13</th><th colspan="2">SWEET14</th></tr>
<tr><th colspan="2">Variant line</th><th>PXO99</th><th>PXO339</th><th>PXO86</th><th>PthXo1</th><th>PthXo2</th><th>TalC</th><th>AvrXa7 PthXo3</th></tr>
<tr><td rowspan="15">IR64</td><td rowspan="2">IR64-5a</td><td rowspan="2">MS</td><td rowspan="2">R</td><td rowspan="2">R</td><td rowspan="2">+1, T</td><td rowspan="2">+2, AA</td><td>-1, A</td><td>+1, C</td></tr>
<tr><td>-3bp</td><td>-7bp</td></tr>
<tr><td>IR64-5b</td><td>MS</td><td>R</td><td>R</td><td>+1, T</td><td>+2, AA</td><td>-1, A</td><td>+1, C</td></tr>
<tr><td>IR64-5c</td><td>MR</td><td>R</td><td>R</td><td>+1, A</td><td>+2, AA</td><td colspan="2">-61bp</td></tr>
<tr><td>IR64-5d</td><td>R</td><td>R</td><td>R</td><td>+1, T</td><td>+2, AA</td><td>-1, A</td><td>+1, C</td></tr>
<tr><td rowspan="2">IR64-7a</td><td rowspan="2">MS</td><td rowspan="2">R</td><td rowspan="2">R</td><td rowspan="2">+1, A</td><td rowspan="2">-1, C</td><td>-1, A</td><td>+1, C</td></tr>
<tr><td colspan="2">-61bp</td></tr>
<tr><td>IR64-7b</td><td>MR</td><td>R</td><td>R</td><td>+1, T</td><td>+2, AA</td><td>-1, A</td><td>+1, C</td></tr>
<tr><td>IR64-9a</td><td>MS</td><td>R</td><td>R</td><td>+1, A</td><td>+1, A</td><td>-44bp</td><td>+1, A</td></tr>
<tr><td>IR64-9b</td><td>MS</td><td>R</td><td>R</td><td>+1, A</td><td>+1, A</td><td>-44bp</td><td>+1, A</td></tr>
<tr><td>IR64-106</td><td>MR</td><td>R</td><td>R</td><td>+1, A</td><td>+2, AA</td><td colspan="2">-62bp</td></tr>
<tr><td>IR64-134a</td><td>R</td><td>R</td><td>R</td><td>+2, TT</td><td>-7bp</td><td>-6bp</td><td>-7bp</td></tr>
<tr><td>IR64-134e</td><td>R</td><td>R</td><td>R</td><td>+2, TT</td><td>-7bp</td><td>-6bp</td><td>-7bp</td></tr>
<tr><td>IR64-134c</td><td>R</td><td>R</td><td>R</td><td>+2, TT</td><td>-8bp</td><td>-6bp</td><td>-7bp</td></tr>
<tr><td>IR64-136</td><td>R</td><td>R</td><td>R</td><td>+1, T</td><td>+1, T</td><td colspan="2">-62bp</td></tr>
</table>

<table>
<tr><td rowspan="26">**Ciherang-Sub1**</td><td>CS-1a</td><td>MR</td><td>R</td><td>R</td><td>-3bp</td><td>-1, C</td><td>-3bp</td><td>+1, A</td></tr>
<tr><td>CS-1b</td><td>R</td><td>R</td><td>R</td><td>+1, A</td><td>+1, A</td><td>-6bp</td><td>+1, A</td></tr>
<tr><td>CS-1c</td><td>MR</td><td>R</td><td>R</td><td>+1, A</td><td>+2, AA</td><td>-3bp</td><td>+1, A</td></tr>
<tr><td rowspan="2">CS-1d</td><td rowspan="2">MR</td><td rowspan="2">R</td><td rowspan="2">R</td><td>+1, A</td><td rowspan="2">-1, C</td><td rowspan="2">-3bp</td><td rowspan="2">+1, A</td></tr>
<tr><td>-3bp</td></tr>
<tr><td rowspan="2">CS-1e</td><td rowspan="2">MR</td><td rowspan="2">R</td><td rowspan="2">R</td><td>+1, A</td><td>+1, A</td><td rowspan="2">-6bp</td><td rowspan="2">+1, A</td></tr>
<tr><td>-3bp</td><td>-1, C</td></tr>
<tr><td>CS-1f</td><td>MS</td><td>R</td><td>R</td><td>+1, A</td><td>+1, A</td><td>-6bp</td><td>+1, A</td></tr>
<tr><td rowspan="2">CS-1g</td><td rowspan="2">MR</td><td rowspan="2">R</td><td rowspan="2">R</td><td>+1, A</td><td rowspan="2">+1, A</td><td rowspan="2">-6bp</td><td rowspan="2">+1, A</td></tr>
<tr><td>-3bp</td></tr>
<tr><td rowspan="2">CS-1h</td><td rowspan="2">MR</td><td rowspan="2">R</td><td rowspan="2">R</td><td rowspan="2">-3bp</td><td>+1, A</td><td rowspan="2">-3bp</td><td rowspan="2">+1, A</td></tr>
<tr><td>-1, C</td></tr>
<tr><td rowspan="2">CS-3a</td><td rowspan="2">MR</td><td rowspan="2">MS</td><td rowspan="2">R</td><td>+1, A</td><td rowspan="2">-1, C</td><td rowspan="2">-11bp</td><td rowspan="2">-20bp</td></tr>
<tr><td>-9bp</td></tr>
<tr><td rowspan="2">CS-3b</td><td rowspan="2">R</td><td rowspan="2">R</td><td rowspan="2">R</td><td>+1, A</td><td rowspan="2">-1, C</td><td rowspan="2">-11bp</td><td rowspan="2">-20bp</td></tr>
<tr><td>-9bp</td></tr>
<tr><td>CS-4a</td><td>R</td><td>R</td><td>R</td><td>-23bp</td><td>+1, A</td><td colspan="2">-62bp</td></tr>
<tr><td>CS-6a</td><td>R</td><td>MR</td><td>MS</td><td>+1, A</td><td>-2bp</td><td colspan="2">-61bp</td></tr>
<tr><td>CS-6b</td><td>MS</td><td>R</td><td>MS</td><td>+1, A</td><td>-3bp</td><td colspan="2">-61bp</td></tr>
<tr><td>CS-6c</td><td>MR</td><td>R</td><td>R</td><td>+1, A</td><td>-2bp</td><td colspan="2">-61bp</td></tr>
<tr><td>CS-6d</td><td>MR</td><td>R</td><td>R</td><td>+1, A</td><td>-2bp</td><td colspan="2">-61bp</td></tr>
<tr><td>CS-6e</td><td>MR</td><td>R</td><td>R</td><td>+1, A</td><td>-2bp</td><td colspan="2">-61bp</td></tr>
<tr><td rowspan="2">CS-6f</td><td rowspan="2">MR</td><td rowspan="2">R</td><td rowspan="2">R</td><td rowspan="2">+1, A</td><td>-2bp</td><td rowspan="2">-6bp</td><td rowspan="2">-1, A</td></tr>
<tr><td>-3bp</td></tr>
<tr><td rowspan="2">CS-6g</td><td rowspan="2">MR</td><td rowspan="2">R</td><td rowspan="2">R</td><td rowspan="2">+1, A</td><td>-2bp</td><td rowspan="2" colspan="2">-61bp</td></tr>
<tr><td>-3bp</td></tr>
</table>

[1] R: resistant, MR: moderately resistant, S: susceptible. The disease assay was repeated three times independently with similar results.
>: SNP, inserted nucleotides are indicated after the comma, deleted nucleotides are not indicated.

Supplementary Table 11. DNA sequencing results for construct validation

**Sample name**	pJS102; pSWEET13-SWEET13:GUSplus
>JS102;Neo21	NNNNNNNGGNNNNCNCCNNNNNTTTTCCCAGTCACGACGTTGTAAAAC<br>GACGGCCAGTGCCAAGCTTGCATGCCTGCAGGTCGACTCTAGAAAGAT<br>TCTAGATCGTAGAAAATGAGACGACTAAATATAGTTAAAGCTATTGAG<br>AGAGTTTTCACTTGTGTCCGATCGATCAAGCCCATTATTCCCCCAATT<br>TATGTCGCATATGAAACACACTAGCTAGCTGAATGAAGCACAAGAGAC<br>ATGCATTATCAGGGAGTAAACGTATGGCAGAGGTTCAGGTAGCCAAGT<br>TCAGACCTGGTTGAGCCAACCTTTCCAAAAGATAGGCGTTCTAGCCGT<br>TCTTTGCTTCCTATTTTTAACAAAATCACTTTAGTTTTCCTCCAATCA<br>TGTTTAGGAGTCAAACACGGTCTCTCCAAACGATGATGATCAGAGGCT<br>AGCTATCACGCAATCATTTATGTCAGAGAGATGTACTTTATTTCAGAC<br>AGTACATATGCATGTCATTCCTTCCAGCAGTGCCACTACTTAGACACC<br>GGTAATTCCAAACTAACCAACCTATATGCATCATGCATGCCAATAAAA<br>TATAACACAGCATGAAAGCGTGCTCACACCCACCCCTACAAACGCATA<br>CACTACTCTAGCCGACATATTTTGAGATTGACGAAATTACCCACGGAC<br>GTCTCGCTGTTTACAACTACACACGAGTATGTTGTGCCTATCATTAAA<br>AGAACAATNAATANGAGTACCTGTATCAAATTTAGGATTTAAATANAT<br>GTATGTATCATATATATTTCGAAATTTACGTATGCTTTANTATATGCA<br>ATTGGNTGCGCANTANCTTGNGCNNCGCCNNNAGNTGATGNAANNNCC<br>NTNNNNNNNCNA
>JS102;WF20	NNNNNNNCNNCGACGGCGCTGAGGGTGAGCGTGAGGGAGAGGGAGAAG<br>GGCATGTACTCCACGCTCCTCGACTGGATCACTCGCTTCTGCAATATC<br>GCATCCATCGTCCATGGAAGCAGTTCAGTTAGCAACGAGATCGATCGA<br>TCAAACCAAGACACGAAGTAAAGATGAGCTAGCTAGAGATTGACGACG<br>ACTGATCGTATATATGCACATACGATGATGCTGAGCGGCGCGACAAAG<br>ACGCTGACGGAGAAGGCGACGCAGACCCAGCCGAGGGAGACGACGCGC<br>TGCTCGCCATGGGAGAGCAGCAGCGTCAGCAGCAGGATCACCCCGAAC<br>ACCCCCACGTTCAGCAGCAGCAGGATCTTCGTCGTGAACACCTGCAAA<br>AACGACGTCGAACCATATATACTTGTCAATTATTATATTTTACTACTA<br>CAAAACGGTAAATATGGAACAATCTTATATAGGTGCCGGTTCTTGTTC<br>ACAACCGATGACACCTGTAAGGGGCCTCTCATCGGTGCCGGTTGCGAC<br>TCGAGAATCAAAGGGTGGTTCCCAAGTCCCCACCGGCATCGAAATAGG<br>GTTTCTGTGGTAGTGCTAGGGTTTGGATGACATGGATCGAACCTTGGC<br>CTTCTTGGGGGCGTAGGCGAGGTACATGACGATGTAGATGGTCTCGAT<br>GACGCAACCGGCGGCGTTGATGGTGATGAGGAGGGCCTCGTTGGACTT<br>GATCAGCGCGTAGAAGATCCACAGCATGGCGCTGAAGAGCGCCACCAC<br>GTACGGCACCGACTGGAACCCCTCCGTCGACTTGCTCTTGTAGATCCG<br>GTAGAACGTCGGGCTGCATAAAATATTATGCACATATATTATCGGATA<br>CGGAACGCGNGCGGATTGGGAATTAAAATTACACAAAAGCTATATATG<br>NNTNTATCATGTANNAAATTCAGTTACCTNCNTTNNNGCCAGATAGNC<br>NNGNNNNATGAANNNGCNGCAAGNNAANNNNNAACNANNAT
>JS102;WF33	CNNGNNNNNNCGGANNGATACGCTGATCCTTCAGATAGGCCGGCACCG<br>TGAACTCACGTTCGTACCAGACATATCCGATATGGTTGCGGATTTCCT<br>TGGTCACGCCAATGTCATTGTAACTGCTTGGGACGGCCATACTAATAG<br>TGTCGGTCAGCTTGCTTTCGTACCACTTCTCTTCCAGTCCTTTCCCGT<br>AGTCCAGCTTGAAGTTCCAGACGCCATTGAGGTCGAAGACGCCACGGG<br>TCTCGGTGTTGATCGGGTACAGACTAGTTCGTCGGTTCTGTAACTATC<br>ATCATCATCATAGACACACGAAATAAAGTAATCAGATTATCAGTTAAA<br>GCTATGTAATATTTACACCATAACCAATCAATTAAAAAATAGATCAGT<br>TTAAAGAAAGATCAAAGCTCAAAAAAATAAAAAGAGAAAAGGGTCCTA

ACCAAGAAAATGAAGGAGAAAAACTAGAAATTTACCCTCAGATCTACC
ATGAGCTCGGTACCCACGGCGGCCACCTCGACGGCGCGCGGCGGNNGA
GGCTTAAGTACTGNTGTTCTTATCCCCTTAATACCTCCACCGACACCA
GCTCCAGCTGCCACTACGTTATGTGGTACTAGATACACTTTCGACTTC
GCAATAGGTGGTGTTCTCATCCCTCCACACCTATTCTCGANCGCACCA
ACAGCNACTAGCCTATCCTGACGCGTGAGTAAGNTCCCNATCTCACNC
NNCTCAGTAGCANTTCNNTCACTANNACCTGCNCCNCTCCNATCCTGA
CCTGTACCATANGATCTCTGTATCTTANCCTCNTCGACGACNCNTGAC
CTCNTCCTCCTGTCATNATCACNACCNTCTACAGATCAAAANACTCT

>JS102;WF39 NNGNNGNGNNNCTGCNNCAGTAATAACTCTGAGCTTATATGTACAAAC
TCAATGGTTTCTAATAAAAAGAAGTAAAATGTTGGCAATGAAGGGAAT
GTCTTAGTTGTGGTAGGTGATGATGATATACCAAGGAGGCCGAAGGCA
AAAGCCCAGGGATGCTGCAGGGACAGGCCAGCCATTTTTGTGTGCTAA
AAGGGGGGTAATTGAAGGGAAGAGATGTAGTGTAATATGGAAGCAAAC
AAGCAAGGAATTGAGCTTGTTGTTGCTAGCTTGGAACTTGTCACAGAC
ACACACACTCTAACCCTAGACCCCCTTTGTGGAGGGAAGGGAGAGCTC
TGAAGAAGAGTTCAATGTGTTGGCTGAAAACTCTTGGAGAGGAATGAA
GGGAGTTGTGGTGCTTTATATAGGAGCAAAGCTAGCTAGGTGGGAATC
ACTTGGTTGCCTCCTACCCATGGCCATTCACGTACGCATCAAAGTAAT
CATAGAATAATTATGCAAAGATTTTTTTTCTTTATTTGCTTTTGAGGA
AAGTCTTTTGGACACGCACAATAATTAGGCTTTTACAAAAATGAGCTA
CTGTTGGAGATAATCCTGGCTAATTTTCAAAAAAAGTTGCATTGGCTT
TGTGCGCAGCGCACCTCTCACTAGCCATAGCCAATTTAGTGAGTAATT
GCTTTGTGCATTGTCAGTAGAGATCGATCTCTCCAACGTCTGCAGCTT
CCTGCTGGGAACGGTAAAAGATAGCCGCCGAGTGCAACAGCAAATCAC
ATGAGATCTCTCCCAGTGAAGGATTATTCCTGAATCCTGATACGGCTT
TTGACTTAAATTTTACTACTCGAAAAATATTAATCGCGGANAAAAAGA
TAATTGTACTTAGACAAAAGAAAGGTAGAATTCTCTGCCTTGTTTCTA
TATATTANNAGAACCGNTTTGTANATGATCTTGCANNTNGGNGANNGA
TACATGTAGGNATACTATANTTTCTCTNNNNTGTTNGNNN

>JS102;WF107 GGNNNNNNNATCTCTCACGTCTGCAGCTTCCTGCTGGGAACGGTAAAA
GATAGCCGCCGAGTGCAACAGCAAATCACATGAGATCTCTCCCAGTGA
AGGATTATTCCTGAATCCTGATACGGCTTTTGACTTAAATTTTACTAC
TCGAAAAATATTAATCGCGGAGAAAAAGATAATTGTACTTAGACAAAA
GAAAGGTAGAATTCTCTGCCTTGTTTCTATATATTAATTAGAACCGTT
TTGTAACATGATCTTGCAATTATTGGGTGATAGATACATGTAGGTATA
CTATAGTTTCTCTGTTCTTTGTTTGCACGGGTAGATTAATTCTCAATG
TCGCCGACAGTGTTCTGAAAAAAAAAATAATGATTGTAATTGAATATGC
AATTCTCCCATGCGCGTGATATAGCTGCTTATTATTTAACTGTCTTTA
CCAAAAGTAACTTATTTTTTGTCGAAGGATTGTTTTCACCTGCAGTAG
ACATCCCTGTCGACTAAGCTTTCATACACAGTTTGTTGGGAAGACAGG
TCGATTTCTGCTTCTGCGAAGCGCGTTGATCTTGTTCTACTCCCACGT
ATATGAAGCTAAGCTAGTGGATCTGCTTTTTTTTTCTCTCTACTTTTT
TCTGTGCGCGCGGCTGCTTGCCTTTCCAATCAACCTATAAGCATAGCA
CAATATATATACGCAATCAATCTGCATATATATAAGCATACTTAATTT
CGAAATATATATGATACATACATATATTTAAATCCTAAATTTGATACA
GGTACTCGTATTTAATTGTTCTTTTAATGATAGGCACAACATACTCGT
GTGTAGTTGTAAACAGCGAGACGTCCGTGGTAATTTCGTCAATCTCAA
AATATGTCGGCTAGAGTAGTGTATGCGTTTGTAGGGGTGGGTGTGAGC
ACGCTTTCATGCTGNGTNATATTTTTATTGGNATGCATGATGCATATA
GGNNNNANTTTGGAATTACCGGNGTCNAGTANNGNNACNG

>JS102;WF108 NNNNNGNNNTNCNNNTTCTTTAATTATTGTGTTACATGGTGCACTATG
ATGTCATTAATTAGAGTACTATATAGCTTGACCTATTAATTCCACTCT

CTCTCCATGGCAACATAAATATATAGAGGTTGCACCAAAGGATCGAAT
GTTTGCTCCACAAGTAGGATATACATACAAATGATGAGGACTTGCTGT
ATATATATAGTTTAACAAGAAGCAGAAAAATTATATGGCTATGTGCAT
CTCATTAAGTATGCTTAGGCCCTTGTAGTTTATATGCTTGTGGATTTT
AAGCCTGCAATTTTAAGGGTGGCGCCGCGCGTATAATTCTAATAGACA
AAAGCCAAAAGAAATAAAAAAAATCAAATCTAGCTTTTGGCTTATTAC
TACAAGAGTACCATCCATATTGCCTTCGGAAGGCTAGTAGGGTTTTTA
GCTTATGCGCTAGTAGCTAATTAATATGCCCAAATAGACATAATAAAC
TGCTGTAAATAAAAGTGCTAGAAGAAATTCTTTTTGTTGAAAATAACT
GAAAGAAGCTAGCTAGAAAATAAAAAACAAATAAAGAAACACAACAAT
TTTATTATTTTATATATGATGACCACTAAAGTGTAATCCTCACTAAAT
GTCGTCGCTAGCTATGCGTATCTGCCAGCAAGTAATAACTCTGAGCTT
ATATGTACAAACTCAATGGTTTCTAATAAAAAGAAGTAAAATGTTGGC
AATGAAGGGAATGTCTTAGTTGTGGTANGTGATGATGATATACCAAGG
AGGCCGAAAGCAAAAGCCCAGGGATGCTGCNGGNCAGGCCAGCCATTT
TTGTGTGCTAAAAGGGGGTAATTGAAGGGAGAGATGTAGTGTAATATG
GAAGCAAACAAGCAAGGAATTGAGCTTGTGTTGCTAGCTTNNNCTTGT
CACAGACACACACACTCTAACCCTANACCCCNNNGTNNNGGNNGNANN
NCTCTGANANANTTCANNNNNNGNNGANNCTNNTNNNANN

>JS102;WF118 NNNANNCTATATATTTATGTTGCCATGGAGAGAGAGTGGAATTAATAG
GTCAAGCTATATAGTACTCTAATTAATGACATCATAGTGCACCATGTA
ACACAATAATTTAAAGAACAAGTTAAAAGCCAAGGCACAATCTTTGTT
GCAAAGGAAGGCAAGGGCTAGACATAAAGTTTGTAAACGTGTCTATCT
ATCACAACTTATCACGTCTGGTCGCTACCTTAGCTGCCTCCATGGGGC
TCTTTACTCGCATTACTTTGCCTTTTTTCCTTTACAAAATCCTGATCT
TGTTTCTGTTCCTTTGGCTTTGCAGGCAACCTCATATCCTTCACGACC
TATCTGGCACCAATGTAAGTAACTGAATTTCTACATGATATATACATA
TATAGCTTTTGTGTAATTTTAATTCCCAATCCGCACCGCGTTCCGTAT
CCGATAATATATGTGCATAATATTTTATGCAGCCCGACGTTCTACCGG
ATCTACAAGAGCAAGTCGACGGAGGGGTTCCAGTCGGTGCCGTACGTG
GTGGCGCTCTTCAGCGCCATGCTGTGGATCTTCTACGCGCTGATCAAG
TCCAACGAGGCCCTCCTCATCACCATCAACGCCGCCGGTTGCGTCATC
GAGACCATCTACATCGTCATGTACCTCGCCTACGCCCCCAAGAAGGCC
AAGGTTCGATCCATGTCATCCAAACCCTAGCACTACCACAGAAACCCT
ATTTCGATGCCGGTGGGGACTTGGGAACCACCCTTTGATTCTCGAGTC
GCAACCGGCACCGATGAGAGGCCCCTTACAGGTGTCATCGGTTGTGAA
CAAGAACCGGCACCTATATAAGATTGTTCCATATTTACCGTTTTGTAG
TAGTAAAATATAATAATTGACAGTATATATGGTTCGACGTCGTTTTTG
CAGTGTCACGACGANATCNGCTGCTGCTGACGTGGGGNNTCGGGNGAT
CNGCTGCTGACGCTGCTGCTCTCCNTGNNANCAGCGCGNC

>JS102;WF119 NNNNNCGNTATTGCAGAGCGAGTGATCCAGTCGAGGAGCGTGGAGTAC
ATGCCCTTCTCCCTCTCCCTCACGCTCACCCTCAGCGCCGTCGTCTGG
TTCCTCTACGGCCTTCTCATCAAGGACAAATACGTCGCGGTAATTATT
CAATCAATTACCTAATTATTTCTTCAAATCACAAACTGCTCAATTCAA
ATTTATGTGTGTGACCACATGAATTGTAATTAAGCTAAAATTGTGTGA
ATTTTTGCAGCTTCCCAACATCCTGGGCTTCACATTCGGTGTGGTCCA
GATGGGGCTCTACGTGTTCTACATGAACGCGACGCCGGTGGCCGGCGA
GGGGAAAGAAGGGAAGGGGAAGCTGGCGGCGGCGGAGGAGCTCCCCGT
CGTCGTCAACGTCGGCAAGCTCGCCGCCGCCACGCCCGACAGGAGCAC
CGGCGCCGTGCACGTGCACCCAGTCCCGAGGAGCTGCGCGGCGGAGGC
GNCAGNGGCCAAGACGGCGTCACTAGGAGATAAAGGCCGAAAACCAGC
ACGGTGAAGCTAAGGAGAAGACTAACTCCAATAGTCCTTGAGGGTGAT
AAATTGCACCACGGGACAGGAGTATTTGAGTACACNAGAAATTAAAAA

	GCGACATAATT
**Note**	Confirmation of DNA sequence of *OsSWEET13* genomic DNA in GUSplus fusion binary vector. Multiple primers were used to confirm genomic clone. This clone was used for Kitaake transformation.
**Sample name**	pJS103;pSWEET14-SWEET14:GUSplus
>JS103;Neo21	GNNNNNNNNNANNCCNNGTTTTCCCAGTCACGACGTTGTAAAACGAC<br>GGCCAGTGCCAAGCTTGCATGCCTGCAGGTCGACTCTAGGTTAACGCT<br>ATGTTTTGATTAAAAAGAGGCACGTGGGACTATGTTCTACCCCTTAAT<br>TTACATGTTATCCAAATAGTTAAAAAAAACTTAAAATCAAACTCAACA<br>TAAATTAAACCATCATAAGTTACCAAAAACATGCACCGCTAAACTTGA<br>TATATCACCCAAATAATCTTTTTATTGGGACGATAATTTCCTATCTTT<br>TCTTTCGACTTAGGAGAAATATAGTCGAATTTAGCTTCTTTTAGTTTA<br>GTTAAGCCATTTAGTTATGTGAAGTTGAAAAAACGTACTTAGGCTATC<br>ACATGGCACTCGTACAAATGCTCCTAAACTGCCACACAGTGGAAATGA<br>TTTTGTTATTATATGAATAATGTATTATGCTTATTTTTGTCATACTAA<br>TGTAATTTCATATATAAAAAGGGACTCAACTTACAAGGCAAAGATACT<br>AAATAGTCGTGATTGGTGAGTGTAGATGTAATTGGGACAAAGTGAATT<br>TTTTTAATATGACATTCAGATATCAAAGTCTATAATATATTTCACAAT<br>ATAAATATTTATTTTTTTAGTGGTTTCTGATTATATTTAGTTTATCGT<br>ACTAATAATGAATACAATGAGGTAGGCTTTCTTTTAGCATTTCTTTTA<br>TGAGCAATAAATAGCATGCCCGCGCATCTGCGCGGGCTTTCTTTCTAG<br>TTCATATTAATTTTAATGAATCTAGACATATATATCTATCTAGATTCA<br>TTAACATCTATATGAATGTGAGAAATGCTAGAATGACTTACATTGTGN<br>AACGGATGGAGTAANAGCTTCTGAATTTATCAACTAANTNNTATATTT<br>CNNNACATGCANAATGCGNTTGNNNCATTAGATACCAANNNTTNANNN<br>GNAAAANNAANNNTTGAAAAAAANNNNNNNTNNNNTNNCN
>JS103;WF33	NNNCNNANNGATACGCTGATCCTTCAGATAGGCCGGCACCGTGAACTC<br>ACGTTCGTACCAGACATATCCGATATGGTTGCGGATTTCCTTGGTCAC<br>GCCAATGTCATTGTAACTGCTTGGGACGGCCATACTAATAGTGTCGGT<br>CAGCTTGCTTTCGTACCACTTCTCTTCCAGTCCTTTCCCGTAGTCCAG<br>CTTGAAGTTCCAGACGCCATTGAGGTCGAAGACGCCACGGGTCTCGGT<br>GTTGATCGGGTACAGACTAGTTCGTCGGTTCTGTAACTATCATCATCA<br>TCATAGACACACGAAATAAAGTAATCAGATTATCAGTTAAAGCTATGT<br>AATATTTACACCATAACCAATCAATTAAAAAATAGATCAGTTTAAAGA<br>AAGATCAAAGCTCAAAAAAATAAAAAGAGAAAAGGGTCCTAACCAAGA<br>AAATGAAGGAGAAAAACTAGAAATTTACCCTCAGATCTACCATGAGCT<br>CGGTACCCGGGGGTGCAGCTACCTTCTTCTCGCCGGCGCCGGCGACGG<br>CGGCGGCGGTGGCGGCGGCGGCCTTGTCGTCCTCCTCAGGCGGCGCCT<br>CTGCCGGCGGGGACTCGACGTCGACGGGGTGCACCTCGCGGGTCTTGA<br>CGACGTCGACGGCGGCAGAGAGCTTGGCGATGTTGACGACGTGCTCCT<br>TGACGCCGGCGGCGGAGTGGTCGTCGTCGCCGGTGGCCGTCGCCGCCT<br>CGACCTCCTTGGTCAGCACGGCCTTGGGCGTCGAGTTCCTGTACATGG<br>CGTACAGCCCCATCTGGATGACGCCGAANGAGAAGCCCAGCACGTTGG<br>GAAGCTGTTCCATTTTGGATCACCATCAGAATTAAAATCGGTNATCAG<br>CTAGCCATAATCACTATAATTAAANATCTTCNCAATTAGAAATAGAAA<br>NNANCNAGCANCGGAAATTTAGGAAGNNNANNGGGTCNANCGGANCTA<br>NNNCANNNNNNNNNNNNNNNNANNNNGAGNNGGCGTANNN
>JS103;WF47	NNNNNTANGATCTAGANTATATATCTATCTAGATTCATTAACATCTAT<br>ATGAATGTGAGAAATGCTAGAATGACTTACATTGTGAAACGGATGGAG<br>TAAATAGCTTCTGAATTTATCAACTAATTATATATTCATTAACATGCA

```
            TATGCGTTGGGTTCATTAGATACCAAGCTTTAAACCCGGAAAAAAGAT
            CATGAAAAAAAGGAAGAACAATTATGTTCTCTCTCCCTAAATGCAGGA
            CTGTTAATTGAAGCAAATAAAGCAGTCCAAGCTAGCTAGGGGAATTAT
            CAGTAGCAGCATACATGGAGAAAGCAAGCAAAAAATTTTGTGGTTATG
            TGCTGAGGAAGAAAGCCATGGAAGAAAGCAAGGGTGCAGTGACGTGTA
            ACAGTCATGGCCAAGCTTGAATGATCTGGCACGTAGAGTGTTCTGGCC
            TGTGCCTGGCACGCTTCTTTTTTGTGTGCCCATATTGCAAAGTAACCG
            TGTGTGTGCCACTCCAACTGATAACTCACCATATAATAACTCTACAGT
            TATTGATAGATATATACATTACGTCCCTTTTTTTAAATAATATCATCC
            AAATATTAGTTAAAAAGGGGGCGTAGTGTATATAGCCAACAGCTGTCA
            AAAAATTGAAAATTACAACGAATATACGTACAATTGCAAAGTGCAAAT
            CACTCTTGTACTATCTTAAAAAGAAATGCAGAGTTTGATCGATTTTGT
            CTCTAATTTTAGGCAACTGGCATTCTGATTTTTTACAAGTGGGTTAAT
            TAGAGCCAGCTGGTCTCAGATCGTCCGTTGGGGAATTAAAGGAGGTGT
            GCTCCTTTGCGGCTCATCAGTTTCTCTAAGCTCTCACCATTCATTCCA
            CTATACAAGCCTAAAGCAGCTAGCTTAGTTAAATTACCTAATAACTAT
            AGCCTTGCCCNACTCTAGATCCCTTACTAGGACAACTTGGNNTACNNA
            ACNATNNNCTANCCATGCNTTNAGGANGGANNNNNAGNNN
>JS103;WF48 NNNNNNANANNCGATGCGGCGGTCGCCGGCGGAGAGGAGGAGGGTGAG
            GAGGAGGATGAGGCCGAAGACGCCGACGTTGACGAGGAGGAGGAGCTT
            GGCGGTGAACATCTTGGCCTTCTTGGGGGCGTAGACGAGGTAGACGGC
            GATGTAGATGGTCTCGATGACGCAGCCAGCGGAGTTGATGGTGATGAG
            GAGGCACTCGTCGGACTTGAGCAGCGCGTAGTAGATCCACAGCATCGC
            GCTGAACAGCGCCACCACGTAGGGTACCGACTGGAACCCCTGCGTCGA
            CTTGCTCTTGTAGATCCTGTAGAACGTCGGCCTGCAAGACATCCAAGC
            AATTGGTTAATTCTTTATATTTTTCCTTGTATTTACTAGCAATGTTAA
            TTGATAGGATTAGCTCTAGACATGTGTATTGGTTGTCTACATCTTAGT
            TATACAGAGACGGGGAGTGTTTTTATCTGAAGAAAAAGGTAATTTTAT
            CATTTTTGATAAGGTACCAATAGTACCACATTAATTTTATGGTGGTGT
            ATAAGAAATTTTAGTGTAAAAGGTACTTTCTGGAGCAATGTAAAATTT
            CGCTAAAGAAAAGAAAATATCCGGATGTGTGGGACTTACAGTGGGGCC
            AGGTAGGTCATGAAGGAGATGATGTTGCCTGCAAAGAGAATAAAATAG
            AAATCATATGTTAGGATCTTGTTTAATTTGATTAACTAAAAACGAAGT
            GCATGCATAAAATGCGACGGACGTTATAATTAAGCGAGTGAACCATGC
            ACAGCAACGTGACACAAGGAGATACGTAACGAACACATATGCATCTAT
            CCTTTTAATAATTGTGTGGGTGACGTCATGTTACACTGATCAGGCCGG
            GCAAGATATTGGAGCCCCTTTTCTTTGAGCTCCTTTTGTCTTGGCTCT
            TTCTGTGCCTGGNTAGGCTTATCNNCAGCAATGCATGTGTAGTATGNA
            TGTATATCTAAATCNCCTCTAANGATGANNNANNNTTCNN
>JS103;WF49 NNANNANGCAGAGTTTGATCGATTTTGTCTCTAATTTTAGGCAACTGG
            CATTCTGATTTTTTACAAGTGGGTTAATTAGAGCCAGCTGGTCTCAGA
            TCGTCCGTTGGGGAATTAAAGGAGGTGTGCTCCTTTGCGGCTCATCAG
            TTTCTCTAAGCTCTCACCATTCATTCCACTATACAAGCCTAAGGCAGC
            TAGCTTAGTTAATTACCTAATAACTATAGCTTGCCCAACTCTAGATCC
            CTTAACTAGGACAACTTGGAGTACACAACAATGTTACTAATCCCATGC
            ATTGAGGACAGAGTTATGAAGGAAACAAAAAAAAGCTAGCAGATTGGC
            ACTTTCTGTCATGCATGGGTGCTGATGATTATCTTGTATCTAATTTAA
            TCAATCCCATGGCTGTGATTGATCAGGAATAGTTTGTGTGTGCAGCTA
            TATTGCCTATTGGTGTCCAGGGTCACACACCATAAGGGCATGCATGTC
            AGCAGCTGGTCATGTGTGCCTTTTCATTCCCTTCTTCCTTCCTAGCAC
            TATATAAACCCCCTCCAACCAGGTGCTAAGCTCATCAAGCCTTCAAGC
            AAAGCAAACTCAAGTAGTAGCTGATTACCAGCTCTTCTCTCTTCTCAT
            TGAGAAGAGGGAATTAAGTTTTGATCTCTGCTTTATTGCCTGATCATC
```

CTCTTGTTACTTGCAAGCAAGAACAGTAGTGTACTGTGCCTCATTGAT
CTCCTCCCACCAAACTCTCTCTCTCTCTCTCATATTCCGAGCTAGCTA
GTTAATCAAGATCTTGCTGCAATGGCTGGCATGTCTCTTCAGCATCCC
TGGGCCTTCGCCTTTGGTCTCCTAGGTGTGTTGCCTTTGATCTGATCC
AAGGAATTCTCTTGAGAATTAATCTTGCATGGGTTATTTACTTTTGNN
NATTANTCTCTACAATTTTTAATCATGTACTTTTCCATGTCCACTTTT
GTTGCCAAAGCCTACNATATTTTTNNACNNNNNTNNNANN

>JS103;WF100 NNNNNNNNNNNGTCGGTACCCTACGTGGTGGCGCTGTTCAGCGCGATG
CTGTGGATCTACTACGCGCTGCTCAAGTCCGACGAGTGCCTCCTCATC
ACCATCAACTCCGCTGGCTGCGTCATCGAGACCATCTACATCGCCGTC
TACCTCGTCTACGCCCCCAAGAAGGCCAAGATGTTCACCGCCAAGCTC
CTCCTCCTCGTCAACGTCGGCGTCTTCGGCCTCATCCTCCTCCTCACC
CTCCTCCTCTCCGCCGGCGACCGCCGCATCGTGGTTCTTGGTTGGGTC
TGCGTTGGCTTCTCCGTCAGCGTCTTCGTCGCCCCCCTTAGCATCATC
GTAAGAAACCCTAGAATTGCCTGTAAACAATAATAACTTTCGGTCTCT
GCACCAACTAAGGATGCACATATTTACGCACCGCTTTTGCTATCTCTG
CAGAGGCTGGTGGTGCGCACCAAGAGCGTGGAGTTCATGCCGTTCTCG
CTCTCCTTCTCCCTCACCATCAGCGCCGTCGTCTGGTTCCTCTACGGC
CTCCTCATCAAGGACAAATATGTCGCTGTGAGTAGCTCCGATTCGACC
CGTTCTTCTTCCTAAATTTTCCGCTGCTCGATTTAATTTCTATTTCTA
ATTGTGGAAGATTGTTTAATTATAGTGATTATGGCTAGCTGATAACCG
ATTTTAATTTCTGATGGTGATCCAAAATGGAACAGCTTCCCAACGTGC
TGGGCTTCTCCTTCGGCGTCATCCAGATGGGGCTGTACGCCATGTACA
GGAACTCGACGCCCAAGCCGTGCTGACCAAGGAGGTCGAGGCGGCGAC
GGCCACCGGCGACGACGACCACTCCGCCGCNGCGTCAAGGAGCACGTC
GTCAACATCGCCAAGCTCTCTGCCGCCGTCGACGTCGTCAGACCCGCG
AGGNGCACCCGGTCGACGTCNAGTCCCCNNNNAGANNCGCCNCNGAGA
GACGANANGNNCCNCNNCACNNNNCNNNNCNNNNNNNNNN

>JS103;WF111 NNNNNNNGNCNNCACCATAAGGGCATGCATGTCAGCAGCTGGTCATGT
GTGCCTTTTCATTCCCTTCTTCCTTCCTAGCACTATATAAACCCCCTC
CAACCAGGTGCTAAGCTCATCAAGCCTTCAAGCAAAGCAAACTCAAGT
AGTAGCTGATTACCAGCTCTTCTCTCTTCTCATTGAGAAGAGGGAATT
AAGTTTTGATCTCTGCTTTATTGCCTGATCATCCTCTTGTTACTTGCA
AGCAAGAACAGTAGTGTACTGTGCCTCATTGATCTCCTCCCACCAAAC
TCTCTCTCTCTCTCTCATATTCCGAGCTAGCTAGTTAATCAAGATCTT
GCTGCAATGGCTGGCATGTCTCTTCAGCATCCCTGGGCCTTCGCCTTT
GGTCTCCTAGGTGTGTTGCCTTTGATCTGATCCAAGGAATTCTCTTGA
GAATTAATCTTGCATGGTTATTTACTTTTGTTGTTATTATTCTCTACA
TTTTTAATCATGTACTTTTCCATGTTCCACTTTTGTTGCCAAAGCTAC
TATATTTTTCCTACCAATTCATCCAAAACTACTATATTATAGCAAGGC
AGCTAGTGGATCGACTTTGCACTTTTGGATGCAATTGTGAGTGGTTTA
CATTAGAGGGGCCATGCATAGCCAAATAAATTGTTTGGCAAAATATGA
TTTCCCTTGTAGTTAGCACAATATTGTGATCTTTCATTCCTTTACTCT
TTTTGTTCCACCACCCTCATTATTGCAAAGCCCCCTCTTTTAGAACCA
AACTAAAGATAAGAAACATTTGATTCATCATTAGAGGAGATTANATAT
ACATACATACTACACATGCATTGCTGTGATAAGCCTAGCCAGGCACAG
AAAGNANCCCAAGAANAAANNAGCTCAAAAGAAAANGGCTCCAATATC
TTGCCNGGCCTGATCAGTGTACATGACGTCACCCNCACNNNATAAAAG
GATAGATGCATATGGNNGTCNNNCNNATCTNNNNNTNTCAN

Note Confirmation of DNA sequence of *OsSWEET14* genomic DNA in GUSplus fusion binary vector. Multiple primers were used to confirm genomic clone. This clone was used for Kitaake

	transformation.
**Sample name**	sweet11-1 k.o
>sweet11-1ko;WF150	CNNNNTCCTACTTCTNCTGATTAACACCTTAGAGTTAGTTAATAACCT<br>TCATCACCAGTAGCAATGCAGGAGGTTTCTTGTCCATGGCTAACCCGG<br>CGGTCACCCTCTCCGGTGTTGCAGGTAAAGCATGCAACCAATGCATAA<br>TGCTCAAACTTAATTTCATCATCATCATCATCATCATCATCTTCACAG<br>CCATGATCATCCATGGACAAATGCAACTGAAGATCATTTTAGTTTTCA<br>TATGCTAATGATCAAATTCAGGTTAATTGCTGA
**Note**	Confirmation of the Cas9-edited site in the homozygous *sweet11-1 knock out* mutant
**Sample name**	sweet13-2 k.o
>sweet13-2ko;WF120	TTTNTCCAAATGGGGNCTAGGGTTAGAGTGTGTGTGTCTGTGACAAGT<br>TCCAAGCTAGCAACAACAAGCTCAATTCCTTGCTTGTTTGCTTCCATA<br>TTACACTACATCTCTTCCCTTCAATTACCCCCCTTTTAGCACACAAAA<br>ATGGCTGGCCTGTCCCTGCTCCCTGGGCTTTTGCCTTCGGCCTCCTTG<br>GTATATCATCATCACCTACCACAACTAAGACATCCCTTCATATTGTAG<br>ATTGNGTGAAACTTGACACAGAGATCTCCTGAAAAAAACCTCAANAAG<br>TATATTATTTGATTTAANTTCACTATTTAAAAAGAAGTTCTTTGTTCC<br>TTTTTTATAAAGAAAA
**Note**	Confirmation of the Cas9-edited site in the homozygous *sweet13-2 knock out* mutant
**Sample name**	sweet14-1 k.o
>sweet14-1ko;WF120	ANCNATNCAGCTCTTCTCTCTTCTCATTGAGAAGAGGGAATTAAGTTT<br>TGATCTCTGCTTTATTGCCTGATCATCCTCTTGTTACTTGCAAGCAAG<br>AACAGTAGTGTACTGTGCCTCATTGATCTCCTCCCACCAAACTCTCTC<br>TCTCTCTCTCATATTCCGAGCTAGCTAGTTAATCAAGATCTTGCTGCA<br>ATGGCTGGCATGTCTCTTCAGCTCCCTGGGCCTTCGCCTTTGGTCTCC<br>TAGGTGTGTTGCCTTTGATCTGATCCAAGGAATTCTCTTGAGAATTAA<br>TCTTGCATGGTTATTTACTTTTGTTGTTATTATTCTCTACATTTTTAA<br>TCATGTACTTTTCCATGTTCCACTTTTGTTGCCAAA
**Note**	Confirmation of the Cas9 edited site in the homozygous *sweet14-1 knock out* mutant

Supplementary Note 1. Standard Operating Procedure HC0002 Screening Genome-edited rice lines for absence of TDNA

STANDARD OPERATING PROCEDURE
HC-0002 Screening Genome-edited rice lines for absence of TDNA

PROJECT: Healthy Crops
DATE: 28-01-2019
ID: SOP No. HC-0002

Project Leader Approval:

PURPOSE:
To ensure that every rice line that was edited using CRISPR-Cas9 and guide RNAs targeting *SWEET11, SWEET13* and *SWEET14* promoters is free of TDNA before it is being sent out.

DEFINITIONS:
rice: *Oryza sativa*
PI: principal investigator
PCR: polymerase chain reaction
Tm: melting temperature
MU: University of Missouri
HHU: Heinrich Heine University
IRRI: International Institute for Rice Research

OPERATIONAL STANDARDS

1. Donor and recipient lab order the primers listed in this table and isolate genomic DNA from the edited rice lines, a non-edited rice line (WT) and a Cas9-positive rice line.

Primer	Sequence	length (bp)	Tm	No. cycles	Ref	Targets
OsCas9-F	GGGTAATGAACTCGCTCTGC		60	27	MU	Cas9
OsCas9-R	TGGCGTCAAGAACTTCCTTTG					
18S_F	CCTAGTAAGCGCGAGTCATC	207	60	27	IRRI	plant DNA
18S_R	GGTTCACCTACGGAAACCTT					
OsCas9-F1	TGACAGGCACTCTATAAAGAAGA	448	56		HHU	Cas9
OsCas9-R1	ATAAAAAGCTTGTCAACATCTGA					
OsCas9-F2	ATGACTAGAAAGTCCGAAGAAAC	464	56		HHU	Cas9
OsCas9-R2	ATGTCCTCCAGTATATCTTCGTT					
TDNA-g11N3_F	GAATGGTCACGATGGATGAT	250	62	30	IRRI	gRNA for SWEET14
TDNA-g11N3_R	GGGGTTATAAGGTGGGTTCT					
g8N3_F	TTGGTGGTGTACAGTAGG	~400	61	30	MU	gRNA for SWEET11 & SWEET13
g12N3_R	CAACTCCCTTCGTTCCTCT					

2. Donor lab isolates genomic DNA (CTAB method) from edited rice lines and from a Cas9-negative (wild-type) rice plant (negative control) and a Cas9-positive rice plant (positive control).

3. Donor lab performs multiplex PCR using primers OsCAs9-F, OsCas9R, 18S_F and 18S_R with the following PCR program (**PCR 1**) and genomic DNA from edited lines and control lines as well as water (additional negative control) as templates.

3.1. 96°C 5:00
3.2. 96°C 0:20
3.3. 60°C 0:20
3.4. 72°C 0:30
3.5. Go to step 3.2, 26x
3.6. 72°C 5:00
3.7. 12°C ∞

4. For samples without a band for Cas9 with primer pairs OsCas9F and OsCas9R, you need to confirm that these plants are Cas9 negative by performing another multiplex PCR with primer pairs OsCas9-F1/OsCas9R1 and OsCas9-F2/OsCas9-R2 and the below PCR program (**PCR2**).

4.1. 96°C 5:00
4.2. 96°C 0:20
4.3. 56°C 0:20
4.4. 72°C 0:30
4.5. Go to step 4.2, 34x
4.6. 72°C 5:00
4.7. 12°C ∞

5. Donor lab performs multiplex PCR using primers TDNA-g11N3-F, TDNA-g11N3_R, g8N3_F and g12N3_R with PCR program below (**PCR 3**). Genomic DNA from a Cas9-negative rice plant and water will be included as negative controls. Genomic DNA from a Cas9-positive rice plant is included as positive control.
5.1. 96°C 5:00
5.2. 96°C 0:20
5.3. 61°C 0:20
5.4. 72°C 0:30
5.5. Go to step 5.2, 29x
5.6. 72°C 5:00
5.7. 12°C ∞

6. Donor lab separates PCR products on 1% agarose gel and adds picture of PCR results from PCR1, PCR2 and PCR3 on page 4 of this SOP and signs the results with date, name of the person who performed the experiments, signature and email address of the responsible PI.

7. Donor lab adds the SOP to the package with the edited rice lines and sends them to recipient.

8. Recipient lab performs steps 1-7.

9. Recipient lab adds picture of PCR results from PCR1 and PCR2 on page 5 of this SOP and signs the results with date, name of the person who performed the experiments, signature and email address of the responsible PI.

10. Recipient lab sends the SOP with PCR results and signatures of both recipient and donor lab to frommew@hhu.de.

11. (**optional**) To verify the edited sites in the *SWEET* promoters, amplify the target promoter regions with these primers and sequence the PCR amplicons.

**Primer**	**Sequence**	**size (bp)**	**Tm**	**No. of cycles**	**targets**
8NKpI-F5	TGAGTGGTCATACGTGTCATATTG	422	60C	34x	SWEET11
8N3-R	CATTGCTACTGGTGATGAAGGT				
12N3-F1	CCGTATCAGGATTCAGGAATA	642	60C	34x	SWEET13
12N3-R1	CCAGCCATTTTTGTGTGCTA				
11N3-F2	GCTCTCACCATTCATTCCACT	663	58C	32x	SWEET14
11N3-R	GGGATGCTGAAGAGACATGC				

DONOR LAB

Name of PI: ________________

Date

Name of Person of performed PCR: _________________

Email PI: ________________

PCR1 results

PCR2 results

PCR3 results

Date

Email PI: ________________

PCR1 results

PCR2 results

PCR3 results

Supplementary Note 2. Material Transfer Agreement for accessibility of kit components.

BIOLOGICAL MATERIAL TRANSFER AGREEMENT

THIS MATERIAL TRANSFER AGREEMENT ("AGREEMENT") is entered into as of the date of last signature of this AGREEMENT (the "EFFECTIVE DATE") between _________________ **("PROVIDER"),** whose address ___________________; and

_________________ (**"RECIPIENT")**, whose address is ___________________.

PROVIDER SCIENTIST:<br><br>Name: _______________<br><br>Address: _______________	RECIPIENT SCIENTIST:<br><br>Name: ____________________<br><br>Address: __________________

MATERIAL [Description of Biological Material to be transferred by PROVIDER to RECIPIENT; pls state in detail]:

____

The "MATERIAL" includes (a) the original MATERIAL, (b) any MATERIAL contained or incorporated in MODIFICATIONS, (c) PROGENY and (d) UNMODIFIED DERIVATIVES. The MATERIAL shall not include (a) MODIFICATIONS (except MATERIAL included therein), or (b) other substances created by the RECIPIENT through the use of the MATERIAL, which are not MODIFICATIONS, PROGENY or UNMODIFIED DERIVATIVES. As used in this AGREEMENT, "PROGENY" means an unmodified descendant from the MATERIAL, such as virus from virus, cell from cell or organism from organism; "UNMODIFIED DERIVATIVES" means substances created by the RECIPIENT which constitute an unmodified functional subunit or product expressed by the MATERIAL (e.g. subclones of unmodified cell lines, purified or fractionated subsets of the MATERIAL, proteins expressed by DNA/RNA supplied by the PROVIDER, or monoclonal antibodies secreted by a hybridoma cell line); and "MODIFICATIONS" means substances created by the RECIPIENT which contain/incorporate MATERIAL.

RESEARCH: The research to be conducted by the RECIPIENT SCIENTIST using the MATERIAL is described in Appendix A attached hereto (the "RESEARCH").

TERMS AND CONDITIONS

1. **Ownership/Use**. The MATERIAL is the property of PROVIDER. Nothing contained in this AGREEMENT shall restrict, modify or limit any ownership rights of the PROVIDER. The MATERIAL is to be used by RECIPIENT solely for the RESEARCH to be conducted by the

RECIPIENT SCIENTIST. The RECIPIENT agrees that the MATERIAL will not be used in human subjects, in clinical trials, or for diagnostic purposes involving human subjects without the written consent of the PROVIDER; is to be used only at the RECIPIENT organisation and only in the RECIPIENT SCIENTIST'S laboratory under the direction of the RECIPIENT SCIENTIST or others working under RECIPIENT SCIENTIST'S direct supervision; will not be used for any commercial purpose or in connection with any commercially-sponsored research and will not be transferred to anyone else (including anyone else within the RECIPIENT'S organisation) without the prior written consent of the PROVIDER; will not be sequenced or otherwise analyzed in order to determine its structure or composition. RECIPIENT agrees to use the MATERIAL in compliance with all applicable statutes and regulations.

2. **Confidentiality**. The MATERIAL as well as all related information provided to RECIPIENT or RECIPIENT SCIENTIST shall constitute the confidential and/or proprietary information of PROVIDER (the "CONFIDENTIAL INFORMATION"). RECIPIENT and RECIPIENT SCIENTIST agree to maintain the confidentiality of the CONFIDENTIAL INFORMATION and to possess and use the CONFIDENTIAL INFORMATION solely for the purposes set forth in this AGREEMENT. As used in this AGREEMENT, CONFIDENTIAL INFORMATION shall not mean or include any information which (a) was known to RECIPIENT or RECIPIENT SCIENTIST prior to the receipt of the MATERIAL or the information from PROVIDER; (b) becomes known to the public without any breach of this AGREEMENT by RECIPIENT or RECIPIENT SCIENTIST; (c) is acquired from a third party without breach of any obligation of confidentiality; (d) is developed independently by RECIPIENT or RECIPIENT SCIENTIST without reference to or reliance on any CONFIDENTIAL INFORMATION; or (e) is required to be disclosed pursuant to subpoena or other judicial or administrative order, or pursuant to applicable law. The obligations of confidentiality contained in this section shall continue, as to any item of CONFIDENTIAL INFORMATION for a period of five (5) years from the date of receipt of such CONFIDENTIAL INFORMATION and shall survive the termination or expiration of this AGREEMENT for any reason.

3. **Availability.** RECIPIENT agrees to refer to PROVIDER any requests for the MATERIAL from other scientists. At its sole discretion, PROVIDER will make the MATERIAL available under a separate AGREEMENT to other scientists for teaching or not-for-profit research purposes only.

4. **Report**. RECIPIENT and RECIPIENT SCIENTIST agree to submit to PROVIDER a written report describing the data generated in the course of the RESEARCH and setting out the results of the RESEARCH (together with the data, the "RESULTS") within six (6) months after the expiration or the termination of this AGREEMENT, whichever is the first to occur. PROVIDER is hereby granted a non-exclusive, royalty-free, perpetual right and license to use the RESULTS for internal academic and research purposes only.

5. **Publication**. In case of joint research, research results shall be published jointly and in mutual consent. If the RESEARCH conducted solely by the RECIPIENT SCIENTIST results in publication, the RECIPIENT SCIENTIST agrees to provide appropriate acknowledgment of the source of the MATERIAL and/or to give credit to PROVIDER or PROVIDER SCIENTIST, as scientifically appropriate, for any contribution which PROVIDER or PROVIDER SCIENTIST may make to the subject matter of the publication. The RECIPIENT

SCIENTIST shall give due consideration to the concerns of the PROVIDER when making scientific publications that relate to the subject matter of the AGREEMENT and, as such, will send the text of the intended publication to PROVIDER before it is published to give the PROVIDER an opportunity to review and comment on it in order to avoid having any applications for IP rights jeopardized by any prior publications that would compromise the novelty thereof or reveal any trade secrets. No later than four weeks after submitting the intended publication to the PROVIDER, and to the extent the PROVIDER has not expressed any objection thereto, the RECIPIENT may publish the findings in accordance with sentence 2. Should the PROVIDER raise any objections, the Parties shall endeavour to arrive at an amicable solution to enable publication.

6. **No Warranty**. The MATERIAL is understood to be experimental in nature and may have hazardous properties. The PROVIDER MAKES NO REPRESENTATIONS AND EXTENDS NO WARRANTIES OF ANY KIND, EITHER EXPRESSED OR IMPLIED, WITH RESPECT TO THE MATERIAL. THERE ARE NO EXPRESS OR IMPLIED WARRANTIES OF MERCHANTABILITY OR FITNESS FOR A PARTICULAR PURPOSE, OR THAT THE USE OF THE MATERIAL WILL NOT INFRINGE ANY PATENT, COPYRIGHT, TRADEMARK, OR OTHER PROPRIETARY RIGHTS.

7. **Liability**. Except to the extent prohibited by law, the RECIPIENT assumes all liability for damages which may arise from its use, storage or disposal of the MATERIAL. The PROVIDER will not be liable to the RECIPIENT for any loss, claim or demand made by the RECIPIENT, or made against the RECIPIENT by any other party, due to or arising from the use of the MATERIAL by the RECIPIENT.

8. **Inventions**. Any patentable invention which relates to new uses of the MATERIAL or which could not have been made but for the contribution of the MATERIAL (an "INVENTION"), will be jointly owned by PROVIDER and RECIPIENT. Any revenues arising from any use or implementation of such INVENTION will be shared by the PROVIDER and the RECIPIENT, with their respective shares to be negotiated in good faith and based on the relative contribution made by the MATERIAL to the INVENTION.

9. **Rights and Licenses**. The RECIPIENT acknowledges that the MATERIAL is or may be the subject of a patent application. Except as provided in this AGREEMENT, no express or implied licenses or other rights are provided to the RECIPIENT under any patents, patent applications, trade secrets or other proprietary rights of the PROVIDER, including any altered forms of the MATERIAL made by the PROVIDER. In particular, no express or implied licenses or other rights are provided to use the MATERIAL or any related patents of the PROVIDER for sale, lease, license, contract research or any other activities directly or indirectly resulting in commercial exploitation (“COMMERCIAL PURPOSES”). If the RECIPIENT desires to use or license the MATERIAL for COMMERCIAL PURPOSES, the RECIPIENT agrees, in advance of such use, to negotiate in good faith with the PROVIDER to establish the terms of a commercial license. It is understood by the RECIPIENT that the PROVIDER shall have no obligation to grant such a license to the RECIPIENT, and may grant exclusive or non-exclusive commercial licenses to others, or sell or assign all or part of the rights in the MATERIAL to any third parties, subject to any pre-existing rights held by others.

10. **Costs**. The MATERIAL is provided at no cost (if no amount is stated below) or for the optional transmittal fee stated below solely to reimburse the PROVIDER for preparation and distribution costs. Costs: _____; Transmittal Fee: _____.

11. **Termination**. Either PROVIDER or RECIPIENT may terminate this AGREEMENT upon the giving of at least thirty (30) days advance written notice. If not sooner terminated, this AGREEMENT shall expire one (1) year after the EFFECTIVE DATE. Upon expiration or termination of this AGREEMENT, RECIPIENT shall, if so directed by PROVIDER, return any unused portions of the MATERIAL or destroy the MATERIAL. Sections 2, 4, 5, 6, 7, 8 and 9 shall survive termination or expiration.

12. **No Assignment**. RECIPIENT shall have no right to assign or otherwise transfer any of its rights in this AGREEMENT or delegate any of its duties under this AGREEMENT without the written consent of PROVIDER.

13. **Governing Law**. General principles of international law shall apply including the UNIDROIT Principles of International Commercial Contracts of 2016. All disputes, controversy or claims arising in connection with this Agreement or its breach, termination or invalidity shall upon mutual agreement, be finally settled in accordance with the Arbitration Rules of UNCITRAL as at present in force, without recourse to the ordinary courts of law.

14. **Miscellaneous**. Any amendments to this AGREEMENT must be executed in writing. The foregoing shall also apply to any waiver of the writing requirement. Should any provision of this AGREEMENT be or become invalid, this shall not affect the validity of the remaining provisions hereof; instead PROVIDER and RECIPIENT agree to replace the invalid provision with a valid provision that most closely reflects the economic intent of the invalid provision.

IN WITNESS THEREOF, the PROVIDER and RECIPIENT have entered into this AGREEMENT as of the EFFECTIVE DATE.

PROVIDER	RECIPIENT
________________<br><br>By: ______________________________<br>[Signature]<br><br>Name: ___________________________<br>[Printed name]<br><br>Title: _____________________________<br><br>Date: _____________________________	____________________<br><br>By: ______________________________<br>[Signature]<br><br>Name: ___________________________<br>[Printed name]<br><br>Title: _____________________________<br><br>Date: _____________________________

As PROVIDER SCIENTIST, I acknowledge and accept the terms and provisions of this AGREEMENT:<br><br>___________________________________<br>[Signature]	As RECIPIENT SCIENTIST, I acknowledge and accept the terms and provisions of this AGREEMENT:<br><br>___________________________________<br>[Signature]

APPENDIX A

The RESEARCH

[Please state in detail]

Supplementary Note 3. Standard Material Transfer Agreement with Additional Terms and Conditions for Transgenic or Biotechnology Engineered (BE) Materials for biological resources of kit components according to the International Treaty on Plant Genetic Resources for Food and Agriculture.

Additional Terms and Conditions to the
Standard Material Transfer Agreement (SMTA) for
Transgenic or Biotechnology Engineered (BE) Materials

SMTA ID No. ______________ **SMTA Date: ____________**

Article 1. Legal basis

The Provider breeds and develops crop materials such as beans, cassava, forages and rice as part of its breeding and biotechnology programs. These materials, including identified or characterized genes or sequences, inserted genes or sequences, DNA or RNA sequences, and plant parts together with associated information constitute **Plant Genetic Resources for Food and Agriculture under Development** (hereinafter referred to as "**PUD materials**") as per articles 5 c) and 6.5 of the SMTA.

The Provider provides access to **PUD materials**, including those *modified or engineered* through the use of *biotechnological* tools and methods resulting in the insertion or not of foreign genes in the plant materials (hereinafter referred to as "**PUD BE materials**") under these **Additional Terms and Conditions** to the SMTA, in accordance with the discretionary right granted to the developer set forth in article 5 c) of the SMTA and article 12 (e) of the International Treaty on Plant Genetic Resources for Food and Agriculture.

Article 2. PUD BE materials

The **PUD BE materials,** governed and provided under the present **Additional Terms and Conditions** and the associated SMTA are listed in *Annex 1* of the SMTA.

Article 3. Definitions

In addition to the definitions contained in Article 2 of the SMTA, the expressions set out below shall have the following meaning:

"***Commercial Purposes***" means the sale, lease, license or transfer of the **PUD BE materials** to any third party. Commercial Purposes shall also include, without any limitation, any use of the **PUD BE materials** aimed at generating an income; producing or manufacturing products for sale; carrying out research or development activities that result in any sale, license, lease or transfer of the **PUD BE materials** to a third party; or to provide a service for third parties incorporating, derived from or using the **PUD BE materials**.

"***Confidential Information***" means all non-public data, knowledge, information and materials of whatever nature and in whatever form, shape or media (including, but not limited to, written, oral and digital form) designated as "Confidential", "Restricted", "Proprietary", "Proprietary and Confidential", "Secret", or with any other similar term, provided by the disclosing party to the receiving party, or declared as such by the parties during the term of this agreement.

"***Derived BE materials***" means any tangible or physical biological material and/or genetic materials and information other than the **PUD BE materials**, which contain or incorporate the **PUD BE materials**, or are derived from the **PUD BE materials**.

"***Intellectual Property Rights***" means any legal right that protects, among other things, inventions, discoveries, ideas, improvements, works of authorship, data, materials, methods, know-how and processes, and includes patents, designs, trademarks, plant variety protection certificates, semiconductor layouts and corresponding applications, as well as copyright, databases, know how, trade secrets, and any similar right including all applications (or rights to apply) for, and renewals or extensions of, such rights recognized from time to time in any jurisdiction of the world, together with all rights of action in relation to the infringement of the above.

"***PUD BE materials***" means **Plant Genetic Resources for Food and Agriculture under Development** that have been *genetically modified through biotechnology tools, transgenic or biotechnology engineered*, including using genome editing techniques, or through technologies that could be subject of regulation in different jurisdictions and contain a trait or traits, or exhibit characteristics or properties due to such processes.

"***Resulting Information***" means any data, information, know how, techniques, practices, methods, processes, testing procedures, software, and any other non-biological materials regardless of the form or media in which they are generated, developed, disclosed, whether or not patentable or susceptible of any other form of legal protection, that are created, discovered, generated or developed as a result of activities carried out with the **PUD BE materials**.

Article 4. Additional Terms and Conditions

The present Additional Terms and Conditions to the SMTA as well as the associated SMTA are used for the transfer of **PUD BE materials** as listed in *Annex 1* in the context and under the project "*Transformative strategy for controlling rice disease in developing countries Phase II*" (the "Project") (Global Development Grant # OPP1155704) lead by the Heinrich Heine Universität (HHU) (Düsseldorf) in collaboration with Centro Internacional de Agricultura Tropical (CIAT), the International Rice Research Institute (IRRI), University of Florida (UF), University of Missouri (UM), and the Institut de Recherche pour le Développement (IRD) (collectively referred to as "Collaborators"). The collaborator's work under the Project is regulated by the Subaward No. F-2016/1166 and the Agreement on a Global Access Strategy for the Project under the grant # OPP1155704.

The **Recipient** accepts to be bound and to respect the following **Additional Terms and Conditions**:

1. The **Recipient** is authorized to use the **PUD BE materials** under a free of charge, non-exclusive, non-transferable, non-commercial, non-sublicensable license for research, test and evaluation, and educational purposes under and for the purposes of the Project. For any other purposes the **Recipient** shall previously consult with the **Provider** and seek a separate agreement.
2. The **Recipient** shall ensure that the **PUD BE materials** are only used for the authorized purposes and under its own premises.
3. Transfer of **PUD BE materials** to any third party is not authorized. Any third party requesting a sample of **PUD BE materials** shall be referred to the **Provider**. For the avoidance of doubt, in case the **Recipient** wishes to transfer the **PUD BE materials** to any of the Collaborators under and for the purposes of the Project, the **Recipient** is entitled to do it by issuing a new Additional Terms and Conditions and associated SMTA and the subsequent recipient shall be bound by the same terms as the original Recipient. The **Recipient** shall notify the **Provider** about such subsequent transfer.
4. Use of the **PUD BE materials** for **Commercial Purposes** or for post-Project development is not authorized. For **Commercial Purposes** or for post-Project development, the **Recipient** must first contact the **Provider** and seek a separate agreement.
5. The **Recipient** shall not claim ownership, neither seek **Intellectual Property Rights** over the **PUD BE materials**, its genetic parts or components, and/or its associated provided information, in the form received from the **Provider**. Further, as applicable to the PUD BE materials, its parts and components and associated provided information, IP protection and the management of Funded Developments under the Project shall be carried out according to the terms agreed upon under the Global Access Strategy Agreement.

6. Any **Resulting Information** and **Derived BE materials** generated or developed by the **Recipient** alone as Funded Developments under the Project shall be property of the **Recipient**, except to the extent of the property on the **PUD BE materials** that lies with the **Provider**. In case the **Provider** is involved in the generation or development of **Resulting Information** and/or **Derived BE materials**, including any invention, ownership shall be joint between the parties and the parties shall proceed as per terms agreed upon under the Global Access Strategy Agreement.
7. The **Recipient** is requested to share all **Resulting Information** with the **Provider**, regardless of ownership, under a non-exclusive, worldwide, free of charge, irrevocable license, for research, evaluation, development, educational and breeding purposes. Following institutional Open Access policies, the **Provider** shall make accessible and available **Resulting Information** through its institutional repositories. If there were **Confidential Information** of the **Provider** or of the **Recipient** linked to or contained in the **Resulting Information**, the **Provider** shall respect this and take it into account for information availability. Further, the parties agree that management of data and publication rights shall be governed by the terms agreed upon under the Global Access Strategy Agreement
8. Regardless of ownership, the **Recipient** shall grant the **Provider** a license to access and use any **Derived BE materials** under free of charge, non-exclusive, non-transferable, non-commercial, non-sublicensable terms for further research, test and evaluation purposes, and for any other purposes contemplated under and for the purposes of the Project. In case of dissemination, post-Project use or **Commercial Purposes**, the parties agree to enter into a separate agreement negotiated under fair and reasonable terms agreed upon by the parties.
9. In addition to the above terms, the **Recipient** shall:
    a. Use the **PUD BE materials** and **Derived BE materials** only in confined environments (e.g., laboratory, growth chamber, and green/glasshouse).
    b. Understand that the term "under its own premises" mentioned in numeral 2 rules out export of **PUD BE materials**.
    c. Use and dispose of the **PUD BE materials** and **Derived BE materials** in compliance with government issued regulations applicable in the geographical area of the **Recipient's** operations and according to institutional regulations/norms applicable to the handling and use of such materials, to transport of such materials and for protection of the environment.
    d. Not use **PUD BE materials** and **Derived BE materials** for any testing in humans or animals, including feeding to or consumption by humans or animals. The **Recipient** shall take all reasonable precautions to prevent **PUD BE materials** and **Derived BE materials** or components thereof from entering food, food products, feed or feed products.
    e. Notify and provide a copy of any proposed disclosure or publication of **Resulting Information** on the **PUD BE materials** and/or **Derived BE materials**, and apply publication rights according to the terms agreed upon under the Global Access Strategy Agreement.
    f. The **Recipient** shall acknowledge the **Provider** as the source of the **PUD BE materials** in any authorized disclosure or publication citing the said materials.
    g. In case of a peer-reviewed publication of **Resulting Information** based on the use of the **PUD BE materials**, the **Recipient** shall give to the **Provider** a copy of a final accepted authors' manuscript or the final printed publication, in print or in electronic form for its inclusion in the **Provider**'s institutional repository to make it available as soon as possible at the time of publication or according to the Open Access policy of the Funding organization of the Project.
    h. Provide an annual written report to the **Provider** on the activities carried out under this agreement or at any other frequency and reporting mode as agreed upon the Global Access Strategy Agreement.
10. The **Recipient** accepts that the **PUD BE materials** are experimental in nature and provided "as is". In no event shall the **Provider** be liable for any use by the **Recipient** of the **PUD BE**

materials or any loss, claim, damage, or liability, of whatsoever kind of nature, which may arise in connection to this agreement or the use, handling or storage of the **PUD BE material**.

11. The **Recipient** will hold the **Provider** harmless and indemnify the **Provider** for any claim made against the **Provider** by a third party arising from or connected with (i) the **Recipient**'s use, handling, storage, or any other activity connected to the **PUD BE materials**; (ii) the **Derived BE materials**; (iii) any breach of this agreement by the **Recipient**; and (iv) any gross negligence or willful misconduct by the **Recipient**.
12. Neither of the parties shall disclose **Confidential Information** to any third party nor use such **Confidential Information** for any other purpose than the expressly authorized by the disclosing party. The receiving party shall use the same degree of care to protect **Confidential Information** as it uses to protect its own information of similar nature, and in any event not less than reasonable care under the circumstances. Confidentiality shall no longer apply to **Confidential Information** if the recipient party can demonstrate that: (i) it had possession of the information prior to disclosure; or (ii) the information is available to the public at the time of disclosure, or becomes available after disclosure through no fault of the receiving party; or (iii) the receiving party receives the information from a third party having the right to such information and who does not impose confidentiality.
13. The **Recipient** shall not assign this agreement to any third party without the prior written consent of the **Provider**. Any purported assignment in violation of the foregoing shall be null and void.
14. All other conditions as outlined in the associated SMTA and agreed to by the **Recipient**, apply except Article 5(a), as provided for in Article 6.5(a) of the SMTA.
15. The present **Additional Terms and Conditions** and the associated SMTA, including all its annexes, contain the whole agreement between the parties in respect of the subject matter and constitute this agreement. Nevertheless, as the parties have entered into a Subaward agreement and associated Global Access Strategy Agreement as above mentioned, the parties agree that if a conflict of terms exists between these Additional Terms and Conditions and the previous Subaward and Global Access Strategy Agreements, the latter shall prevail only to the extent of such conflicting term and only to the extent applicable to the PUD BE materials transferred under the present Additional Terms and Conditions.
16. The present **Additional Terms and Conditions** shall be in force and be binding upon the **Recipient** for the period of validity of the associated SMTA, unless the **Provider** agrees otherwise in writing.

I, the undersigned, hereby represent that I am legally authorized to sign these Additional Terms and Conditions on behalf of the Recipient legal entity

Name of Recipient legal entity: ______________________________________

Name of Recipient's authorized representative: _________________________

Position of authorized representative: _________________________________

Signature: ____

Date: ____
